# Cannabis sativa extracts inhibit LDL oxidation and the formation of foam cells *in vitro*, acting as potential multi-step inhibitors of atherosclerosis development

**DOI:** 10.1371/journal.pone.0310777

**Published:** 2024-12-20

**Authors:** Bruno Musetti, Alejandra Kun, David Menchaca, Alejandra Rodríguez-Haralambides, Javier Varela, Leonor Thomson, Edward M. Bahnson

**Affiliations:** 1 Facultad de Ciencias, Instituto de Química Biológica, Laboratorio de Enzimología, Universidad de la República, Montevideo, Uruguay; 2 Department of Cell Biology & Physiology, University of North Carolina at Chapel Hill, Chapel Hill, NC, United States of America; 3 Facultad de Ciencias, Biología Celular del Sistema Nervioso Periférico-DPAN-IIBCE, Instituto de Investigaciones Biológicas Clemente Estable, Sección Bioquímica, Montevideo, Uruguay; 4 CIBERNED-España, Madrid, Spain; 5 Laboratorio Química Bioanalítica, Instituto Polo Tecnológico de Pando, Facultad de Química, Universidad de la República, Uruguay; 6 Facultad de Ciencias, Laboratorio de Química Orgánica y Medicinal, de la República, Uruguay; University of Brescia: Universita degli Studi di Brescia, ITALY

## Abstract

Atherosclerotic disease is the leading cause of death world-wide. Our goal was to explore the effect of phytocannabinoids on the molecular mechanisms triggering the development of the atheromatous lesion. Three cannabis sativa extracts of different chemotypes were chemically characterized by UPLC-DAD. The capacity of the extracts to prevent the oxidation of LDL, the formation of foam cells and the activation of an inflammatory response by J774 cells, were monitored by UV-Vis spectrometry, confocal-microscopy and western blot. Three varieties of cannabis sativa, with high (**E1**), intermediate (**E2**) and low (**E3**) THC/CBD ratios were selected. The three cannabis extracts inhibited the oxidation of LDL by copper ions and the formation of foam cells by J774.1 cells challenged with oxLDL (ED_50_ 5–12 μg mL^-1^). The effect of the cannabinoid extracts on the endocytic process was independent of the canonical cannabinoid receptors, CB1 and CB2, but related to the action of non-canonical receptors (TRPV1, TRPV4 and GPR55), involved in calcium signaling. Decreased levels of CD36 and OLR1 scavenger receptors were, at least partially, responsible for the diminished uptake of oxLDL induced by phytocannabinoids. The downregulation of CD36 and OLR1 could be explained by the observed inhibitory effect of the cannabis extracts on the activation of the NFκB pathway by oxLDL. Phytocannabinoids interfere with the main events leading to the development of the atheromatous plaque, opening new venues on atherosclerosis therapy.

## Introduction

Atherosclerosis is one of the main causes of human disease and the first cause of death worldwide [1]. The combined role of the low-density lipoprotein (LDL) oxidation and inflammation in atherosclerosis have been largely demonstrated [2]. Macrophages orchestrate atherosclerosis by the engulfment of oxidized or aggregated LDL through scavenger receptors (SR). The association of SR to oxidized lipoproteins leads to intracellular Ca^2+^ rise and the production of superoxide and hydrogen peroxide by the inducible NADPH-oxidase (Nox2) [2,3]. Calcium and oxidants promote the nuclear translocation of the nuclear factor κB (NFκB) [4], activating the production of cytokines and highly oxidizing species from Nox2 and inducible nitric oxide synthase (NOS2) [5,6].

The activation of the NFκB pathway stimulates the synthesis of scavenger receptors which are responsible for the internalization of oxLDL and consequent foam cell formation [7]. The oxidized LDL receptor 1 (OLR1) interacts through a positive feedback loop with the canonical NFκB pathway [8], whereas the scavenger receptor A1 (SRA1) is regulated indirectly by the canonical NFκB pathway [9]. In the case of the cluster of differentiation 36 (CD36), it is reported that the alternative NFκB pathway regulates the expression of CD36 [10]. Cannabinoids are involved in cardiovascular physiology [11,12]. For example, the expression of the cannabinoid receptor type 1 (CB1) was increased in coronary atherosclerotic plaques from cardiovascular disease (CVD) patients and the concentration of the main endocannabinoid mediators, N-arachidonoylethanolamine (AEA, anandamide) and 2-arachidonoylglycerol (2-AG), were also enhanced in human coronary atherectomy samples and in plasma from atherosclerotic patients [13]. Vasodilation, bradycardia, and reduced blood pressure were observed after intravascular infusion or topical skin application of anandamide [14]. Previous studies found that CB1 activation is proatherogenic, promoting vascular inflammation, oxidative stress, and endothelial dysfunction [15]. Inversely CB2 activation is anti-atherogenic [15–17] and CB2 agonists are being developed for vascular disorders [18].

Cannabinoids also interact with the transient receptor potential (TRP) family of membrane channels and other non-CB1/CB2 G-protein coupled receptors (GPR) such as GPR55 [19,20]. These ion channels have a relatively non-selective permeability to cations, including sodium, calcium, and magnesium, participating in the regulation of the function of intracellular organelles, such as endosomes and lysosomes [21]. In particular, the vanilloid receptor 1 (TRPV1), which is involved in the regulation of calcium signaling [22], modulates several pathways related to the development of the atherosclerotic lesion. For example, in a mice model of atherosclerosis, a specific agonist of TRPV1 significantly reduced the aortic lesions and decreased the accumulation of lipids in vascular smooth muscle cells (VSMC) in vitro [23,24]. On the other hand, TRPV4 activation is associated with phenotypic switch by macrophages, and calcium influx via TRPV4 also activates NFκB expression [25,26].

We have recently shown that extracts obtained from cannabis sativa are able to inhibit the oxidation of LDL [27]. In the present work the influence of extracts obtained from three chemotypes of cannabis sativa on the main steps leading to the development of atheromatous plaque, i.e.: the oxidation of LDL and the uptake of modified LDL by J774.1 cells leading to the formation of foam cell were tested. The studies support the potential beneficial effects of cannabinoids in atherosclerosis through the interaction with non-canonical cannabinoid receptors, and the therapeutic potential of medicinal cannabis.

## Results

### Sample characterization

**Fig 1:**
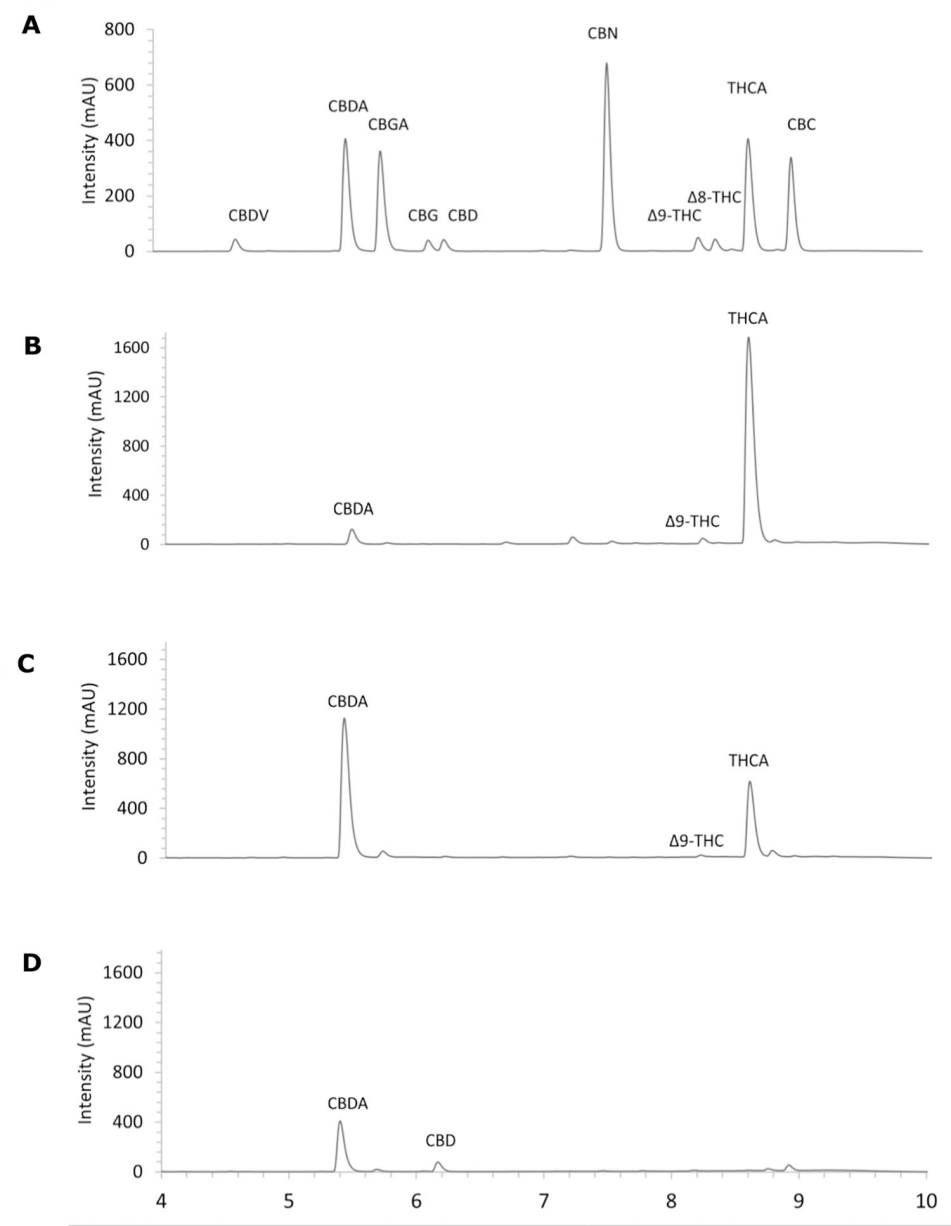
UPLC-DAD profile (270 nm) of phytocannabinoids in the extracts separated in C18 column. Representative elution profile of the phytocannabinoid standards (50 μg mL^-1^ each) (A), as well as the three extracts (500 μg mL^-1^) with different chemotypes: E1 (B), E2 (C) and E3 (D). temporal scale in minutes.

The phytocannabinoid composition of the extracts was determined by ultra-high-performance liquid chromatography coupled to a diode array detector (UPLC-DAD), using ten of the most abundant phytochemical species as standards (**Fig 1**). The chemical profiles of the three cannabis chemotypes are shown in **Table 1**. The chemical profiles of the three cannabis chemotypes are depicted in Fig 1 and the quantification of cannabinoids are shown in Table 1. The predominant cannabinoids in E1 were THC and its direct precursor THCA, contributing together to more than 85% of the total cannabinoid content in this variety, confirming its psychotropic or intoxicant characteristics. The content of THC(A) in **E2** was lower than 50% of the total cannabinoids, with a significant increase of CBDA and a THC(A)/CBD(A) ratio ~1, consistent with an intermediate chemotype. The extract derived from hemp (**E3**) showed a chemical profile of a typical “fiber”, rich in CBDA and CBD and with a low content of the psychotropiccompounds. The solvent used for the extract preparation (methanol:chloroform 9:1) was selected to maximize the extraction of phytocannabionids, as recommended by the American Herbal Pharmacopeia for the characterization and quantification of phytocannabinoids [28]. The presence of flavonoids, terpenoids, carotenoids and other organic acids seems to be null, as we showed before by NMR and UV-visible spectrometry [27], and in the UPLC-DAD analysis (Fig 1).

**Table 1:** Chemical characterization of phytocannabinoids in the extracts.

Cannabinoid	RT (m)	E1		E2		E3	
		(μg.mg^-1^)	(%)^a^	(μg.mg^-1^)	(%)	(μg.mg^-1^)	(%)
**CBDV**	4.65	3.43 ± 0.01	0.6	ND	-	3.0 ± 0.1	1
**CBDA**	5.52	25 ± 5	4.3	301 ± 9	60.1	99.6 ± 0.7	32.4
**CBGA**	5.79	3.0 ± 0.1	0.5	12.6 ± 0.1	2.5	3.7 ± 0.1	1.2
**CBG**	6.05	4.8 ± 0.2	0.8	2.5 ± 0.5	0.5	5.9 ± 0.2	1.9
**CBD**	6.16	6.2 ± 0.1	1.1	16.7 ± 0.8	3.3	175 ± 1	57.1
**CBN**	7.56	2.51 ± 0.05	0.4	0.35 ± 0.02	0.1	0.56 ± 0.01	0.2
**Δ** ^**9**^ **THC**	8.27	73 ± 5	12.8	30 ± 1	5.9	11.8 ± 0.1	3.9
**Δ** ^**8**^ **THC**	8.4	29 ± 3	5.1	3.0 ± 0.9	0.6	ND	-
**Δ** ^**9**^ **THCA**	8.67	422 ± 6	74.0	133 ± 5	26.6	0.39 ± 0.01	0.1
**CBC**	8.99	1.20 ± 0.04	0.2	1.7 ± 0.6	0.3	6.9 ± 0.2	2.3
**Total cannabinoids**		570	100	500	100	307	100
**THC(A):CBD(A) ratios** ^**b**^		16		0.5		0.03	

^a^Percentage (%) of cannabinoids relative to the total detected cannabinoids determined by UPLC-DAD analysis.

^b^THC(A):CBD(A) ratios are the ratios between the concentrations of THC plus THCA over the concentration of CBD plus CBDA.

ND, non-detected.

### Inhibition of LDL oxidation

**Fig 2:**
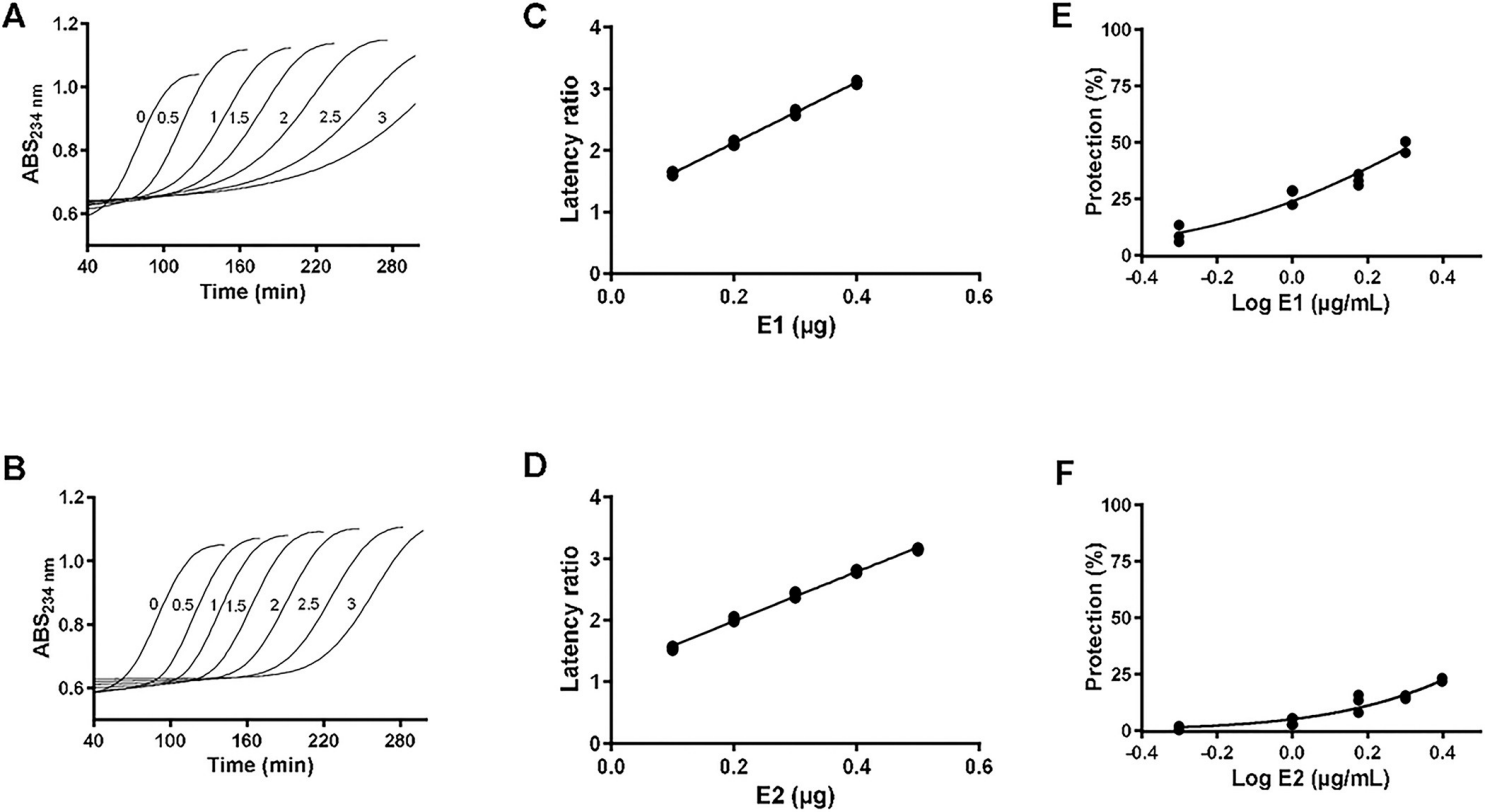
Inhibition of Cu^2+^-mediated of LDL oxidation by cannabis extracts. The oxidation of LDL (0.1 mg mL^-1^) was initiated by the addition of CuSO_4_ (50 μM) and monitored at 234 nm. The assays were performed in the absence or presence of increasing concentrations of extract **E1** (**A**) and **E2** (**B**)) (0.5–3 μg mL^-1^). The correlation between the ratio of latencies (latency/control latency) and the amount of extract (μg) are shown for **E1** (**C**) **and E2** (**D**). Correlation between the protection and the concentration of extract are shown for **E1** (**E**) **and E2** (**F**).

The oxidative modification of LDL makes it a target for scavenger receptors initiating the formation of the atheromatous plaque. The capacity of the new extracts with different relative composition of phytocannabinoids to inhibit the oxidation of LDL was tested. The extracts obtained from **E1** and **E2** acted as effective antioxidants, prolonging the latency phase of LDL oxidation and as effective radical scavenger slowing down the rate of the propagation phase (**Fig 2**). The antioxidant capacity (AC μg^-1^), showing how many times the latency phase of the lipid oxidation was increased by each μg of extract added, was 4.9 ± 0.1 and 4.0 ± 0.1 μg^-1^ for **E1** and **E2,** respectively. Previously, we reported that **E3** (hemp) showed an antioxidant capacity of 3.7 ± 0.1 μg^-1^, [27] which is similar to the other chemotypes. The AC is proportional to the total cannabinoid concentration of the extracts (**S1A Fig**). However, the main driver of the AC appears to be the Δ^9^THC content of the extracts as it accounts for >99% of AC variability between the extracts (R^2^ = 0.9984) (**S1B Fig**). Additionally, the linear fit includes the AC value of pure Δ^9^THC (**S1 Fig** and **S1 Table**). Moreover, the concentration necessary to achieve a 50% (IC_50_) decrease of the propagation phase slope was also proportional to the Δ^9^THC content of the extracts (**S1C Fig** and **S1 Table**) in agreement with the already described protection by THC and lack of protection by THCA, CBDA and CBD [27].

### Inhibition of foam cell formation by cannabis extracts

**Fig 3:**
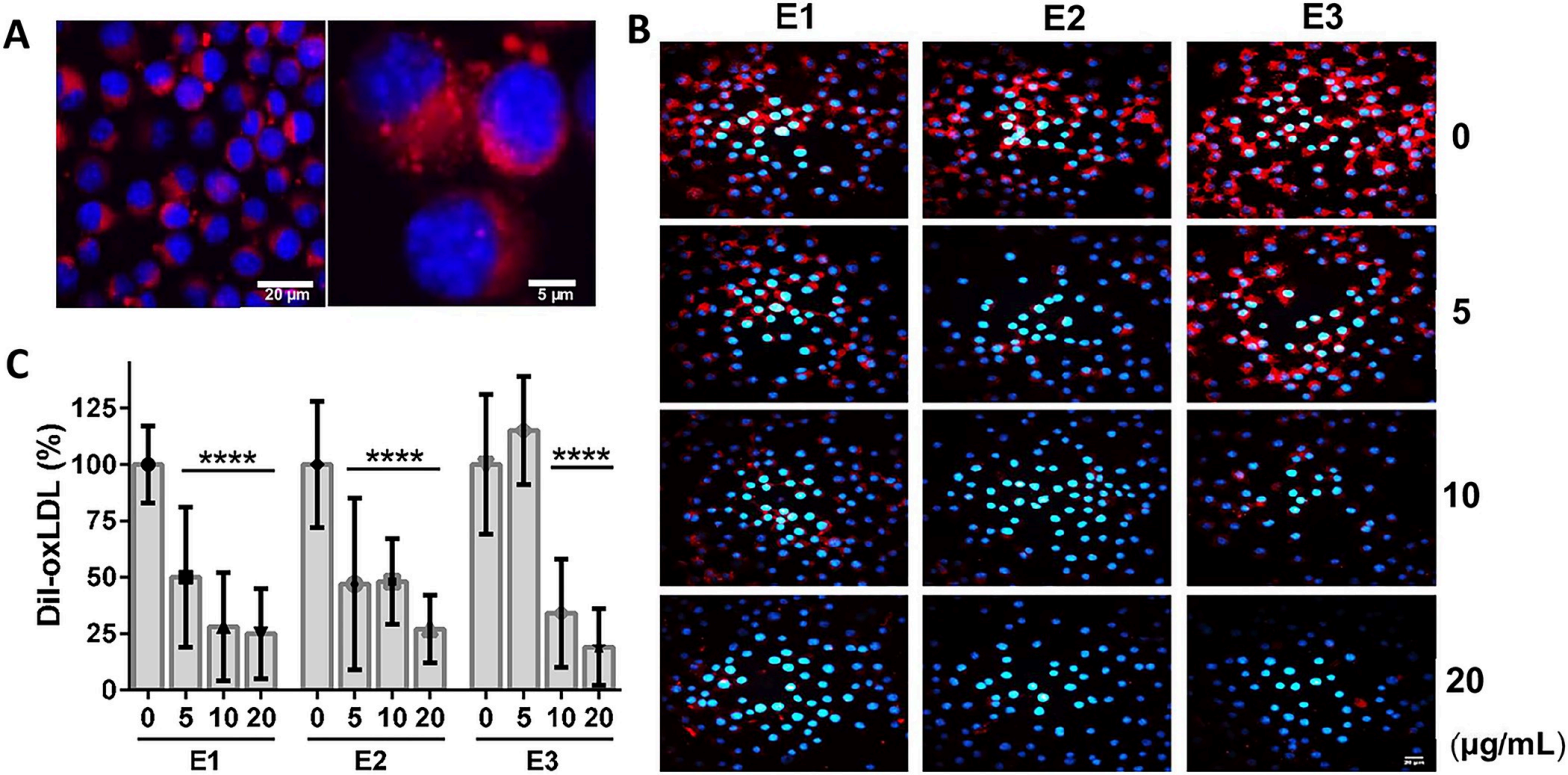
Inhibition of foam cell formation. **A**. J774.1 cells were incubated with DiI-oxLDL (10 μg mL^-1^) for 4 hours at 37°C. Nuclei were stained with DAPI and cells were visualized using confocal microscopy. A 3.5x zoom is shown on the right to visualize the fluorescent lipoprotein distribution. **B**. The cells were incubated with vehicle (0.2% DMSO) or with increasing concentrations (5–20 μg mL^-1^) of the three cannabis extracts (**E1**, **E2** and **E3**) for 1 hour before being exposed to DiI-oxLDL (10 μg mL^-1^) for 4 hours. **C**. Images from three independent experiments were evaluated using ImageJ. The results were expressed as a percentage of the fluorescent signal compared to the condition with DiI-oxLDL without extract. Statistically significant differences between incubations without and with the extracts in three independent experiments were found using One-way ANOVA followed by Dunnett’s multiple comparisons test (****, p < 0.0001). All images have where taken at 400x magnification. Scale bar = 20 μm.

Murine macrophages (J774.1 cells) were able to internalize oxLDL associated with the fluorescent lipophilic tag DiI. As shown by SLMC, accumulation of oxLDL displayed a vesicular pattern in the cytosolic area of the cells (**Fig 3**). To assess the ability of the extracts to inhibit the formation of foam cells, J774.1 murine macrophages were incubated in the absence and the presence of increasing concentrations (5–20 μg mL^-1^) of the three extracts (**E1**, **E2** and **E3**) for 1 hour before being exposed to DiI-oxLDL (10 μg mL^-1^) for 4 h. The signal corresponding to the intracellular Dil-oxLDL complex decreased as the amount of the cannabis extracts in the incubation mixture increased (**Fig 3**).

**Fig 4:**
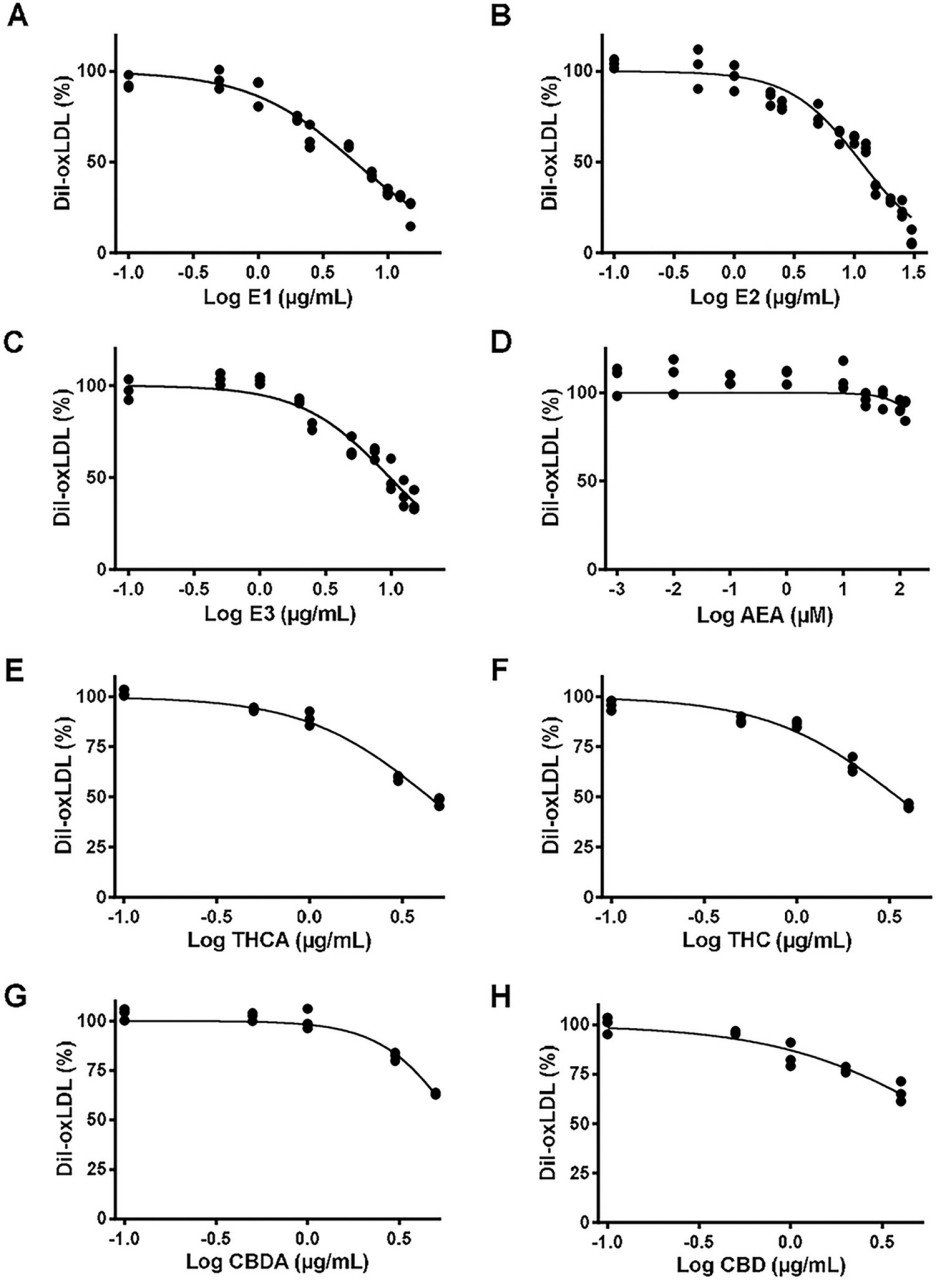
Dose response curves of the phytocannabinoids effect on oxLDL uptake by J774.1 cells. The cells were incubated in the presence of DiI-oxLDL (10 μg mL^-1^) for 24 h and increasing concentrations of **E1** (0.5–30 μg mL^-1^) (**A**), **E2** (0.5–30 μg mL^-1^) (**B**), **E3** (0.5–30 μg mL^-1^) (**C**), AEA (0.001–125 μM) (**D**), THCA (0.1–4 μM) (**E**), THC (0.1–4 μM) (**F**), CBDA (0.1–4 μM) (**G**) and CBD (0.1–4 μM) (**H**). Internalized DiI-oxLDL fluorescence in each condition was evaluated at λex = 540 nm λem = 564 nm. Results are expressed as percentage of a vehicle-treated control condition (0.2% DMSO). Three independent experiments were performed, and dose-response curves were used to determine the ED_50_ shown in Table 2.

To quantitatively determine the inhibitory action of the extracts on oxLDL internalization, a spectrofluorometric technique to measure DiI-oxLDL in the cellular fraction was developed (**S2 Fig**). A sigmoidal decrease of the fluorescence from the internalized DiI-oxLDL complex was observed with increasing concentrations of the extracts (**Fig 4**). The THCA richer extract (**E1**) was the most effective one decreasing oxLDL internalization by the cells, with an effective dose 50 (ED_50_) of 5 μg mL^-1^. The extract from the intermediate variety (**E2**) and the hemp derived product (**E3**) showed a lower effectiveness with ED_50_ of 12 and 10 μg mL^-1^, respectively (**Table 2**). Conversely, anandamide (AEA), an endogenous agonist with preference for CB1 receptors that also binds TRPV1 with lower affinity [29,30], was unable to prevent the uptake of oxLDL by the cells (**Fig 4**). To identify the ligands responsible for the effect of the cannabis extracts on oxLDL internalization the isolated phytocannabinoids were used. THCA, THC, CBDA and CBD inhibited oxLDL endocytosis by the cells (**Fig 4**), with ED_50_ values in the micromolar range (**Table 2**).

**Table 2:** Effective range on J774.1 cells.

	ED_50_	LD_50_	TI
	(μg mL^-1^L)	
**E1**	5 ± 1	33 ± 2	7
**E2**	12 ± 1	> 40	NT
**E3**	10 ± 1	28 ± 3	3
	(μM)	
**AEA**	NE	105 ± 1	NE
**THCA**	12 ± 1	> 40	NT
**THC**	11 ± 1	20 ± 1	1.8
**CBDA**	18 ± 3	> 40	NT
**CBD**	25 ± 5	23 ± 1	0.9

ED_50_, determined from the dose-response graph of oxLDL internalization.

LD_50_, determined from the viability curves (MTT).

TI, therapeutic index (LD_50_/ED_50_).

NT, non-toxic in the explored range.

NE, non-effective.

To assess if the effect of the extracts was explained by the additive effect of the cannabinoid composition, we plotted the theoretical additive effect of the main cannabinoid components of each extract and compared the oxLDL uptake inhibition with the experimental data for each extract (**S3 Fig**). The effect of each of the extracts is greater than the expected additive effects of the cannabinoid components, which suggest either a synergistic effect of the extracts or the presence of a low-abundance compound with a large inhibitory effect (**S3 Fig**).

### Cell survival and metabolic activity

To rule out the potential effect of cannabinoids on cell metabolism and survival underneath the inhibitory action on the endocytosis of ox-LDL, the capacity of the J774.1 cells to reduce the metabolic probe MTT, a substrate of the mitochondrial succinate dehydrogenase [31], was analyzed (**S4 Fig**). MTT assayed in the same condition as oxLDL internalization showed values of lethal dose 50 (LD_50_) for the cannabis extracts several times higher than the values of ED_50_ for the endocytosis of oxLDL (**Fig 4**). In particular, the LD_50_ for **E1** was more than six times higher than the ED_50_ obtained for the endocytosis of oxLDL, while with **E2** the cells remained metabolically active even in the presence of concentrations as high as 40 μg mL^-1^ (**Table 2**), ruling out the cell death or an impairment of the energy metabolism as responsible for the inhibitory effect of the extracts on the uptake of oxLDL. The extract **E3** obtained from a hemp variety showed higher toxicity and had the lowest therapeutic index (**Table 2**). The endocannabinoid AEA showed a low toxicity, with an LD_50_ value higher than 40 μM (**Table 2**).

The individual phytocannabinoids showed different toxicity depending on the presence of the carboxylic group (**S4 Fig**). In fact, the acidic forms, THCA and CBDA, caused no effect on the cell viability and metabolism in the explored concentrations range (3–30 μM). On the other hand, the LD_50_ values determined by the MTT technique were close to the ED_50_ values in the case of the decarboxylated phytocannabinoids, THC and CBD with TI values close to one (**Table 2**).

### Effect of receptor antagonists

**Fig 5:**
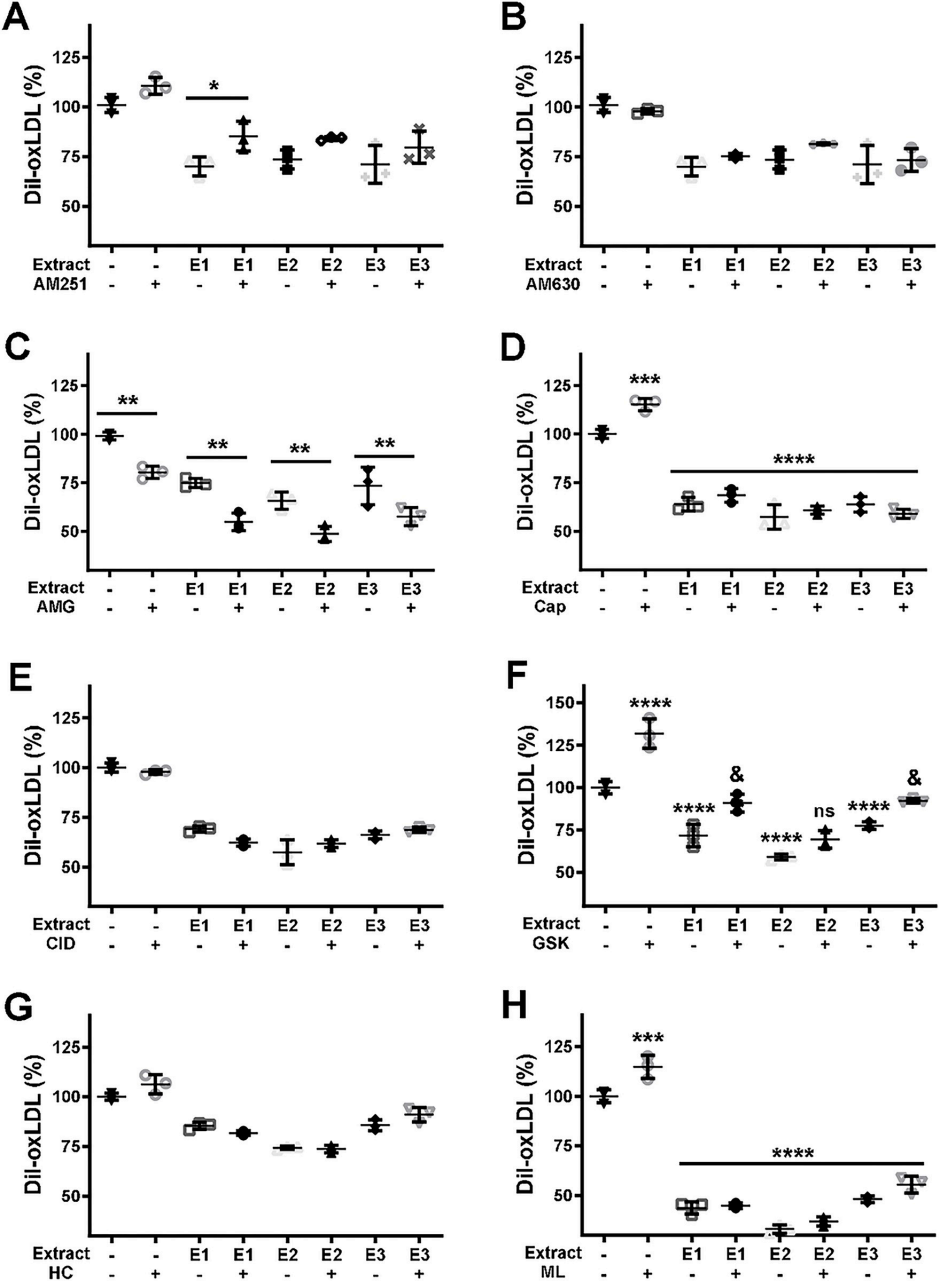
Participation of canonical and non-canonical receptors in the inhibitory action of the extracts on oxLDL internalization. The cells were incubated for 24 h with oxLDL (10 μg mL^-1^) without (-) or with **E1** (2 μg mL^-1^), **E2** (5 μg mL^-1^) and **E3** (5 μg mL^-1^), in the absence (-) and the presence (+) of the antagonists of the CB1 (AM251, 5 μM) (**A**), CB2 (AM630, 5 μM) (**B**), TRPV1 (AMG9810, AMG, 5 μM) (**C**), TRPV4 (HC067047, HC, 10 μM) (**E**) and GPR55 (CID160246, CID, 8 μM) (**G**). The effect of the agonists of the TRPV1 (capsaicin, Cap, 50 μM) (**D**), TRPV4 (GSK1016790A, GSK, 10 μM) (**F**) and GPR55 (ML184, ML, 10 μM) (**H**) were also tested but with incubations for 6 h. The results are presented as mean and SD from three independent experiments. One-way ANOVA followed by Dunnett’s multiple comparisons test was used to compare the condition with the extract alone and the same extract plus antagonist or agonist (*, p < 0.05; **, p < 0.01; ***, p < 0.001; ****p < 0.0001).

To identify the cannabinoid receptor involved in the inhibitory effect of the cannabis extracts on oxLDL internalization by J774.1 cells, the effect of specific antagonists of the receptors were used. The selection of agonists and antagonists and the concentrations used were selected to minimize cross-reactions and are based on the reported inhibition constants (Ki) or their IC_50_ (**S2 Table**). The extract concentrations were chosen to inhibit about 30% of oxLDL internalization and the concentrations of the agonists and antagonists were a thousand times higher than the reported Ki values for their respective receptors, to assure an optimal competition. The cells were incubated with DiI-oxLDL in the presence of antagonists of the canonical receptors, CB1 (AM251) and CB2 (AM630). Inhibition of CB1 or CB2 for 6 h did not influence the inhibition of ox LDL uptake caused by any of the extracts (**S5 Fig**). While the effect of AM251 (5 μM, Ki_CB1_ = 8 nM [32]), a CB1 ligand with affinity for this receptor ~20 times higher than for CB2 and more than a thousand times higher than for other GPR and TRP receptors (**S2 Table**), on oxLDL uptake was statistically significant at 24 h, it has a modest effect size (ω_p_ = 0.44). Post-hoc analysis at 24 h inhibition, revealed a significant difference between the effect of **E1** on oxLDL uptake with or without AM251 (p<0.05). However, the interaction between AM251 and cannabinoid extract was not significant (F(3,16) = 0.3516, p = 0.7886), suggesting again that CB1 is not involved in the ability of the cannabinoid extracts to inhibit oxLDL uptake. Similarly, incubation with AM630 (5 μM) for 24h, a CB2 antagonist (Ki_CB2_ = 31 nM vs Ki_CB1_ = 5152 nM [30] was unable to counteract the inhibitory effect mediated by any of the three extracts (**Fig 5**). In line with the lack of effect of anandamide described above, these results suggest that the canonical cannabinoid receptors (CB1 and CB2) do not play a major role on the inhibitory action of the cannabis extracts on the oxLDL uptake by J774.1 cells.

The participation of TRPV1, TRPV4 and GPR55 receptors, also responsive to phytocannabinoids [20,33,34], was investigated. The TRPV1 antagonist AMG9810 (5 μM, IC50s = 24.5 and 85.6 nM for human and rat TRPV1, respectively [22]), showed significant inhibition of DiI-oxLDL uptake with a large effect size (F(1,16) = 81.37, p<0.0001, ω^2^_p_ = 0.77). This effect was independent of the cannabinoid extracts (**Fig 5**) (F(3,16) = 0.2195, p = 0.8814 for the interaction of extracts and AMG9810). The antagonist of GPR55 (CID160246, 8 μM, Ki = 63 nM [35]) and TRPV4 (HC067047, 10 μM, Ki = 17–133 nM [36,37]) did not affect DiI-oxLDL uptake either in the absence or the presence of the extracts (**Fig 5**). Likewise, the effect of the agonists on these receptors was scarce with the incubations for 24 h (**S6 Fig**), but with incubations for 6 h the three agonists induced a clear increase (15–30%) of the DiI-oxLDL internalization in the absence of extracts. Capsaicin (50 μM, Ki = 94.8 nM [22]), an agonist of the TRPV1 receptors, increased the DiI-oxLDL uptake by the cells by ~15%. The effect of capsaicin was nulled by all extracts (**Fig 5**) with a clear interaction between the agonist and extracts (F(3,16) = 7.549, p = 0.0023, ω^2^_p_ = 0.45). This suggests that TRPV1 participates in the regulation of oxLDL uptake and that the phytocannabinoids present in the extracts can counteract the stimulatory effect of TRPV1 activation. Similarly, the agonist of TRPV4 (GSK101, 10 μM, Ki = 5 nM [36]) had a significant and large effect on oxLDL uptake by the cells (F(1,16) = 89.05, p<0.0001, ω^2^_p_ = 0.79). In the absence of the extracts, GSK101 increased the uptake by ~30%. The effect of the extracts was partially reversed by GSK101 with a clear interaction between the agonist and the extracts (F(3,16) = 5.311, p = 0.0099, ω^2^_p_ = 0.35). Finally, the GPR55 agonist (ML184, 10 μM, Ki = 260 nM [38]) also increased DiI-oxLDL uptake ~15%. As in the case of capsaicin, the effect of ML184 was nulled by all extracts with a clear interaction between agonist and extracts (F(3,16) = 4.9, p = 0.0133, ω^2^_p_ = 0.33). These results highlight the participation of non-canonical receptors in the formation of foam cells and in the inhibitory effect of the phytocannabinoids. Importantly, cell viability under the assayed conditions remained unchanged in the presence of the antagonists and agonists (**S7 Fig**), excluding the possibility that lower cell viability could account for the observed effects. These results reinforce previous reports on the participation of transient receptor potential cation channels and GPR55 on oxLDL internalization [24,39–42], linking the inhibitory effects of cannabinoids on foam cell formation to calcium signaling. In fact, we used our model to confirm the previously reported effect of Ca^2+^ on oxLDL internalization [43,44]. The chelation of intracellular Ca^2+^ by BAPTA-2AM had a very considerable impact on the amount of DiI-oxLDL internalized by the J774.1 cells (**S8 Fig**). Likewise sequestering the extracellular Ca^2+^ by the addition of EGTA, the transport of DiI-oxLDL into the cells becomes negligible (**S8 Fig**).

### Effect of cannabinoids on the expression of scavenger receptors

**Fig 6:**
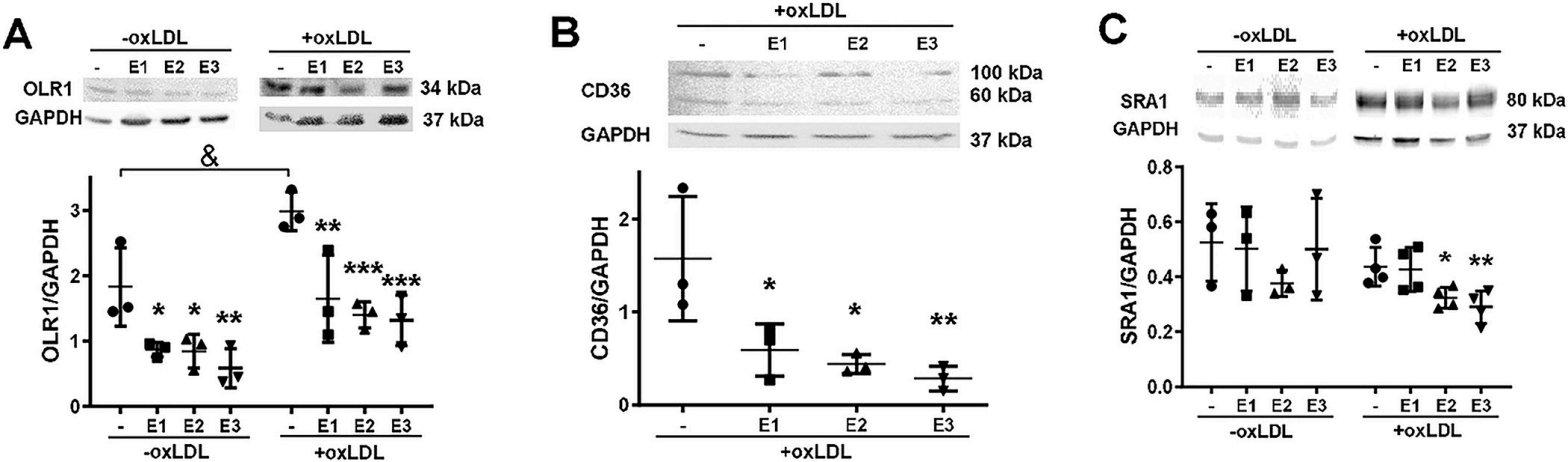
Effect of the cannabis extracts on the accumulation of scavenger receptors. **A**. J774.1 cells were incubated for 24 h with 15 μg mL^-1^ of the extracts or vehicle (-) (0.2% DMSO), in the absence (left) and presence of 10 μg mL^-1^ oxLDL (right bands). Cell lysate proteins were separated by 12% SDS-PAGE and the presence of OLR1 and GAPDH immunodetected. Representative images of the signals corresponding to the OLR1 and GAPDH proteins obtained by western blot are shown. The mean ± SD of the densitometric analysis, expressed as the ratio of OLR1/GAPDH, from three independent experiments is shown. Using one-way ANOVA followed by Dunnett’s multiple comparisons test, significant differences were found in the amount of OLR1 present in the unstimulated condition and the oxLDL stimulated one (&, p < 0.05), and between the cells incubated in the absence and the presence of the extracts (*, p < 0.05, **, p < 0.01; ***, p<0.001). **B**. J774.1 cells were incubated for 24 h with oxLDL (10 μg mL^-1^) and the cannabinoid extracts (15 μg mL^-1^) or vehicle (0.2% DMSO). Cell lysate proteins were separated by 12% SDS-PAGE and the presence of CD36 and GAPDH immunodetected. Representative images of the signals corresponding to the CD36 and GAPDH proteins obtained by western blot are shown. The mean ± SD of the densitometric analysis, expressed as the ratio of CD36/GAPDH, from three independent experiments is shown. One-way ANOVA followed by Dunnett’s multiple comparisons test were used to compare the conditions without and with the extracts (*, p < 0.05; **, p < 0.01). **C**. J774.1 cells were incubated for 24 h with oxLDL (10 μg mL^-1^) and the cannabinoid extracts (15 μg mL^-1^) or vehicle (0.2% DMSO). Cell lysates proteins were separated by 12% SDS-PAGE and the presence of SRA1 and GAPDH immunodetected. Representative images of the signals corresponding to the SRA1 and GAPDH proteins obtained by western blot are shown. The mean ± SD of the densitometric analysis, expressed as the ratio of SRA1/GAPDH, from three independent experiments is shown. One-way ANOVA followed by Dunnett’s multiple comparisons test were used to compare the conditions without and with the extracts (*, p < 0.05; **, p < 0.01).

Since the expression of the scavenger receptors induced by oxLDL depends on calcium-mediated signaling processes [45,46], the effect of the cannabinoid extracts on the relative amount of CD36, SR-A1 and OLR1 in the cells was explored. The expression of the OLR1 which increased > 50% in the presence of oxLDL (p < 0.05), decreased after exposing the cells for 24 h to the cannabis extracts (15 μg mL^-1^) (F(3,16) = 16.76, p<0.0001, ω^2^_p_ = 0.66), both in unstimulated cells and in cells stimulated with oxLDL with no significant interaction (F(3,16) = 0.6093, p < 0.6186) (**Fig 6**). In the case of the CD36 receptor, the basal expression in these cells was undetectable in agreement with the published literature [47,48] and with publicly available gene expression datasets (**S9 Fig**). Incubation of the cells with 10 μg mL^-1^ oxLDL for 24 h led to the appearance of two immunoreactive bands, one with an apparent molecular mass of ~60 kDa, consistent with the unmodified receptor, and the other at ~100 kDa, compatible with the highly post translationally modified receptor [49] (**Fig 6**). The three cannabis extracts significantly decreased the amount of this receptor under oxLDL challenge (**Fig 6**) (F(3,8) = 7.272, p = 0.0113, ω^2^_p_ = 0.61). The expression of the SR-A1 receptor, which remained unaltered by the presence of oxLDL, moderately decreased with **E2** and **E3** (**Fig 6**). Our results point out the expression of the scavenger receptors as an important target of phytocannabinoid action. The nuclear factor NFκB has been pointed as a major player linking atherosclerosis and inflammation [4,6,50–52]. NFκB represents a family of transcription factors normally kept inactive in the cytoplasm through interaction with inhibitory molecules of the IκB family, the canonical pathway of NFκB activation involves the release of the heterodimer p65/p52 associated with IκB. In response to multiple stimuli, including calcium and oxidants, the kinase of IκB (IKK) is activated [53–55]. The IKK-mediated phosphorylation leads to polyubiquitination and destruction of IκB by the proteasome. Released NFκB enters the nucleus and activates the transcription of a diversity of immune and inflammatory genes [56–58]. Activation of scavenger receptors, oxidants as well as calcium can trigger NFκB activation, and the activation of this pathway may induce the synthesis of scavenger receptors [59,60].

**Fig 7:**
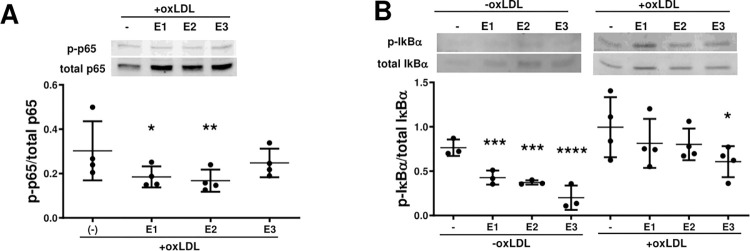
Effect of the cannabis extracts on the activation of the NFκB pathway. **A.** Semiconfluent J774.1 cells were incubated in a 6 well plate for 4 hours in the absence and presence of oxLDL (10 μg mL^-1^), without (-) and with the cannabis extracts **E1**, **E2** and **E3** (15 μg mL^-1^). Wester blots for p-p65 and total p65 and densitometric analysis normalized to total p65 are shown **B.** The cells were incubated for 4 hours in the absence and presence of oxLDL (10 μg mL^-1^), without and with the cannabis extracts (15 μg mL^-1^). Representative wester blots for p-IκBα and total IκBα, and densitometric analysis of p-IκBα normalized to total IκBα are shown. The results are presented as mean and SD from at least three independent experiments. One-way ANOVA followed by Dunnett’s multiple comparisons test were used to compare the conditions without and with the extracts (*, p < 0.05; **, p < 0.01; ***, p < 0.001; ****p < 0.0001).

Since phytocannabinoids were able to interfere with each one of these signaling processes, the capacity of the cannabis extracts to interfere with the activation of this pro-inflammatory transcription factor was explored. The phosphorylation of NFκB subunit p65 at ser536 was evidenced after oxLDL incubation of the cells, and although the signal decreased in the presence of the three extracts, it was significant only in the presence of **E1** and **E2** (**Fig 7A**). For its part, the phosphorylation of IκBα was evident even in unstimulated cells, being higher in the oxLDL challenged cells. The three extracts were able to significantly decrease the phosphorylation level of IκBα in the absence of oxLDL, but only **E3** was able to decrease the signal in the presence of oxLDL (**Fig 7B**).

## Discussion

The oxidative modification of LDL, mediated directly by oxygen and nitrogen derived oxidants, enzymes, and metal ions, is one of the initial steps leading to atherosclerosis [61]. Spectrophotometric as well as electrochemical methods demonstrated the antioxidant capacity of phytocannabinoids [62,63]. In these reactions, the phenolic hydroxyl(s) in phytocannabinoids gives up a hydrogen atom turning into a quinone radical, stabilized by resonance [64–66]. In a previous paper we reported the capacity of cannabis extracts and isolated phytocannabinoids to protect LDL from Cu^2+^-mediated oxidation [27]. In those samples, the antioxidant capacity of the extracts increased with the content of THCA in the cannabis extracts because of the maturation of the inflorescences. In the present study, we investigate the capacity of cannabis sativa extracts with different relative content of phytocannabinoids to prevent the oxidation of LDL. The extracts were able to interfere in both phases of lipid oxidation, retarding the beginning and decreasing the rate of the oxidative chain of radical mediated reactions induced by Cu^2+^. These results reinforce previous reports on the antioxidant properties of phytocannabinoids [62,63,67,68].

The next step in the development of atheromatous plaque is the recognition and endocytosis of modified LDL by scavenger receptors, located on the surface of different cell types involved in the vascular lesion [69]. The extracts from three chemotypes of cannabis were able to inhibit the internalization of oxLDL by a macrophage-like cell line. The inhibitory action of the cannabis extracts on the uptake of oxLDL appears to be mediated by the phytocannabinoids present in the extract mixture since THC, THCA, CBD and CBDA effectively inhibited the endocytosis of oxLDL, being the non-psychotropic phytocannabinoids carrying the carboxylic group (THCA and CBDA), the molecules with the highest therapeutic indexes. However, the effect of the extracts was greater than the addition of the inhibitory effect of the individual cannabinoid components. This synergy or more than additive effect could be explained by the entourage effect [70]. The term was first coined by Ben-Shabat et al. to explain that non-active metabolites potentiated the effect of the endocannabinoid 2-arachidonoylglycerol [71]. Individual components could exhibit additive effects, their combined impact is simply the sum of their individual effects; antagonistic interactions, or synergistic interactions when compounds produce an effect surpassing the sum of their individual contributions. Cannabinoid-cannabinoid interactions, cannabinoid-terpene, and terpene-terpene interactions could account for intra or inter entourage effects [72]. Our observation that the extracts as a whole exhibit stronger inhibition than the sum of the effect of the component cannabinoids supports either an entourage effect or the additive effect of a low-abundance component with potent bioactivity.

About the target receptors interacting with the phytocannabinoids, an antagonistic interplay between phytocannabinoids and TRPV1, TRPV4 and GPR55 receptors appeared as relevant to oxLDL accumulation into the cells and the inhibitory effect of the extracts. Although the expression of CB1 and CB2 receptors in J774.1 has been demonstrated [73,74], the effect of phytocannabinoids on oxLDL internalization in this murine macrophage-like cell line was independent of those receptors. Several transient potential and G protein-coupled receptors have been also described in J774.1 cells [75–77], and TRPV1 TRPV4 and GPR55 appeared as the receptors involved in the effect of phytocannabinoids on the cellular accumulation of oxLDL. However, using cells that overexpress recombinant human TRPV1, it was reported that, although some phytocannabinoids can stimulate TRPV1 receptors inducing an increase in cytosolic Ca^2+^, most of the compounds were little (CBD and CBDA) or not effective (THC and THCA). Additionally, several compounds, including CBD, CBDA, and THCA, desensitized the cells to the stimulatory effect of capsaicin on Ca^2+^ uptake [20]. This decrease in the influx of calcium could explain the decrease in the internalization of oxLDL induced by the extracts, desensitizing the cells to the stimulatory effect of oxLDL, and acting in an analogous way as the antagonists of the vanilloid receptor. Additionally, the relevance of TRPV4 receptors in foam cell formation by oxLDL-exposed macrophages has been shown using specific inhibitors and knockout mice, where ablation of these receptors prevented foam cell formation [39]. Likewise, the increase of intracellular Ca^2+^ induced by phytocannabinoids was low compared to that triggered by the typical TRPV4 agonist 4-α-phorbol-12,13-didecanoate (4α-PDD) [33]. However, phytocannabinoids were more effective in desensitizing this channel after activation by 4α-PDD [33]. In our cell model, the activation of the channels involving calcium signaling showed a significant and opposite effect than the extracts, increasing the internalization of the modified lipoprotein. These results point to calcium and non-canonical receptors, including vanilloid receptors, as negatively related to the action of phytocannabinoids on foam cell formation, acting in the same antagonistic direction already shown in other inflammatory pathologies [33]. However, the antioxidant properties of phytocannabinoids can also contribute to ameliorate the signaling events involving the production of reactive oxygen and nitrogen species triggered by the cellular insult (oxLDL), acting in a similar way as described before in a cellular model of glutamatergic neurotoxicity [67].

Related to the signaling of non-canonical cannabinoid receptors and calcium, there is a clear dependence of the internalization of oxLDL and the mobilization of Ca^2+^ [44,78,79]. In contrast to classical phagocytosis mechanisms and LDL receptor-mediated internalization, the endocytosis of oxLDL by CD36 is carried out through clathrin- and caveolin-independent pathways [80]. Importantly, oxLDL uptake depend on influx of extracellular Ca^2+^ [81] and assembly of F-actin [43]. Inhibition of macrophage Ca^2+^ channel or chelation of intracellular Ca^2+^, both inhibit oxLDL uptake [82,83]. Moreover, the activity and expression of scavenger receptors also depends on Ca^2+^ either directly or indirectly [82,84,85]. The expression of these receptors is induced by pattern recognition receptor (PRR) ligands, such as modified LDL [86], through signaling pathways involving NFκB, PKC, and Ca^2+^ [87,88]. The formation of foam cells in our model clearly depends on Ca^2+^ and the treatment with cannabis extracts caused a decrease in the relative amount of OLR1 and CD36 receptors in J774.1 cells, consolidating our hypothesis on the antagonistic effects of phytocannabinoids on Ca^2+^-mediated signaling mechanisms. However, a direct antioxidant effect of the extracts inside the cells, analogous to the action on LDL oxidation, cannot be discarded, especially since it could lead to the inhibition of redox-sensitive signaling pathways. Interestingly, our results show that the basal protein level of CD36 in unstimulated J774.1 macrophages is undetectable by immunoblot. This agrees with several publications supporting the low expression levels of CD36 protein under basal conditions is low in J774 cells [47], and its expression increases after oxLDL challenge [47,48]. The same behavior is seen in other cell types and stimulus [89–91].

Activation of scavenger receptors elicit pro-inflammatory responses in immune and vascular cells through NFκB activation [9,51]. Moreover, scavenger receptor synthesis depends on active NFκB [56,59,60], resulting in a positive feedback loop. The OLR1 gene has an NFκB cis element and hence, its expression is regulated by canonical activation of NFκB [8,92]. On the other hand, oxLDL increases both SRA1 and OLR1 expression in an NFκB -dependent manner [9,59,60]. However, the SRA1 gene is not directly controlled by p65/p50 binding [8]. Finally, NFκB is not necessary for the LPS-induced CD36 increase in macrophages [60]. Hence, the literature supports that NFκB–induced oxLDL uptake depends on the increased expression of OLR1 and SRA1. On the contrary, phytocannabinoids decreased IκB phosphorylation and reduced ser536 phosphorylation (potentially indicative of IKK activity [93,94] leading to lower NFκB activation, substantiating the decreased expression of scavenger receptors and in consequence a minor oxLDL capture by the cells.

Our work is not without limitations. Firstly, we used a murine macrophage-like model with very low expression of CD36 [47]. The role of CD36 in human atherosclerosis has been well studied and its relevance to human disease is not to be overlooked [95,96]. However, it is also true that in humans SRA1 and OLR1 are important drivers for disease [97–101]. However, it is important to acknowledge that because CD36 expression is very low in J774 macrophages, our study is not designed to address the potential role of cannabinoids on CD36-dependent disease mechanisms. Secondly, we focused our work on macrophages. Atherosclerotic plaques are complex and multicellular. Moreover, it has been shown by lineage tracing studies [102,103], that VSMCs are the main cellular component of the atherosclerotic plaque. Macrophages are the second most prominent cell type in the atherosclerotic plaque [104]. A thorough recent study by Mocci et al. found 8 macrophage clusters in human plaques [104] and integrated their data with a comprehensive human cardiometabolic database comprising 600 patients (Stockholm-Tartu Atherosclerosis Reverse Network Engineering Task Study) [105]. They found three gene regulatory networks with robust association with clinical scores of coronary disease severity. Of these three groups, one was VSMCs, and two were macrophages. Especially one macrophages group was associated with proinflammatory macrophages in symptomatic plaques via lipid accumulation and increased inflammation signaling pathways [104]. These recent data support our decision to focus on the mechanisms by which cannabinoids could inhibit both lipid accumulation, and pro-inflammatory signaling. Finally, we recognize that we do not have *in vivo* data supporting our findings. Whereas the protective effect of cannabinoids on murine atherosclerosis progression has been studied [17], it is not known whether our findings decreased NFκB signaling, and decreased scavenger receptor levels, causally explains the previously reported *in vivo* findings.

Our results highlight the capacity of phytocannabinoids to ameliorate the processes leading to the development and progression of atherosclerotic lesions through inhibiting LDL oxidation, decreasing the formation of foam cells after oxLDL challenge and reducing scavenger receptor synthesis by interfering with NFκB activation, supporting the therapeutic potential of medicinal cannabis in atherosclerosis and the need to unravel the molecular mechanisms of phytocannabinoids on the cardiovascular system.

## Materials and methods

### Materials

Cannabinoid reference standards (≥ 97% purity) Δ^8^-tetrahydrocannabinol (Δ^8^-THC), cannabidivarin (CBDV), cannabigerolic acid (CBGA), cannabigerol (CBG), cannabichromene (CBC), and cannabinol (CBN) were purchased from Merck KGaA™. Δ^9^-tetrahydrocannabinolic acid (THCA), cannabidiolic acid (CBDA) (≥92% purity), Δ^9^-tetrahydrocannabinol (THC), and cannabidiol (CBD) (≥98% purity) were purchased from Echo Pharmaceuticals™. The following cannabinoid receptor ligands were used: the CB1 antagonist 1-(2,4-Dichlorophenyl)-5-(4-iodophenyl)-4-methyl-N-1-piperidinyl-1H-pyrazole-3-carboxamide (AM251) and the CB2 antagonist 6-Iodo-2-methyl-1-[2-(4-morpholinyl)ethyl]-1H-indol-3-yl](4-methoxyphenyl)methanone (AM630) (Santa Cruz Biotechnology), the TRPV-1 antagonist 2E-N-(2,3-dihydro-1,4-benzodioxin-6-yl)-3-[4-(1,1-dimethylethyl)phenyl]-2-propenamide (AMG9810) (ABCAM), the TRPV-1 agonist 8-methyl-N-vanillyl-6-nonenamide or capsaicin (ABCAM), the GPR55 agonist 3-[[4-(2,3-dimethylphenyl)-1-piperazinyl]carbonyl]-N,N-dimethyl-4-(1-pyrrolidinyl) benzenesulfonamide (ML184, Sigma-Aldrich), the GPR55 antagonist 4-[4,6-dihydro-4-(3-hydroxyphenyl)-3-(4-methylphenyl)-6-oxopyrrolo[3,4-c]pyrazol-5(1H)-yl]benzoic acid (CID16020046), the TRPV4 antagonist 2-methyl-1-(3-morpholin-4-ylpropyl)-5-phenyl-N -[3-(trifluoromethyl)phenyl]pyrrole-3-carboxamide (HC067047) (Santa Cruz Biotech.), the TRPV4 agonist N-[(1S)-1-[[4-[(2S)-2-[[(2,4-dichlorophenyl) sulfonyl]amine]-3-hydroxy-1-oxopropyl]-1-piperazinyl]carbonyl]-3-methylbutyl]benzo [b]thiophene-2-carboxamide (GSK1016790A) (Santa Cruz Biotech.), the endocannabinoid and CB1 and CB2 agonist N-arachidonoylethanolamine or anandamide (AEA) (Santa Cruz Biotechnology). These ligands were selected in base to their affinity and relative specificity for the receptors, in base to already reported affinities (see **S2 Table**).

### Inflorescence sampling and preparation of extracts

Fresh samples of the female inflorescences were collected at the end of the flowering period from three stable Cannabis sativa cultivars. The samples were obtained from a registered cannabis club (#42) based in Montevideo, Uruguay. The varieties analyzed (**E1**, **E2** and **E3** (hemp)) were selected based on marked differences in cannabinoid composition. Extracts of quintuplet samples (1 g each) were obtained by dynamic maceration for 20 minutes in methanol: chloroform (9:1). The resulting extracts were filtered and then brought to dryness using a rotary evaporator and stored at -20° C until use. Immediately before use the samples were solubilized in DMSO (cellular assays) or in acetonitrile (quantitative analysis) at the desired concentration.

### Quantitation of cannabinoids

Each extract (~ 100 mg) was dissolved in acetonitrile, sonicated, passed through 0.2 μm filters and stored in sealed vials. The content of the main cannabinoids in the samples was evaluated by ultra-performance liquid chromatography (Nexera, Shimadzu, Kyoto, Japan) equipped with diode-array detector (SPD-M30A), column oven (CTO-20A) and auto sampler (SIL-30A). Chromatograms were acquired using the *LabSolutions* software (version 5.52 SP2, Shimadzu, Kyoto, Japan). The absorbance was monitored at 270 nm and photodiode-array signals were acquired in the 190–800 nm range. The samples (3 μL) were injected in a C18 reversed-phase column (1.6 μm, 2.1 mm x 100 mm, CORTECS® UPLC Waters, Milford, USA) with a C18 Security-Guard Ultra (AJ0-8782, Phenomenex, California, USA), using a controlled column temperature of 30 ± 0.2°C. The elution was performed at 0.3 mL min^-1^, with a mobile phase consistent in (A) 50 mM ammonium formate with 10% acetonitrile (v/v) pH = 3,75 and (B) acetonitrile with 0.1% formic acid (w/v). The gradient started with 55% B, increasing to 90% B in 8 minutes, and returning to initial conditions at 8.01 minutes to re-equilibrate for 2 minutes. The concentration of the different phytocannabinoids was calculated from standard curves performed in parallel using known concentrations of the standards.

### Isolation of LDL from human plasma

The LDL fraction was isolated from human plasma using a density gradient as described before [27,106]. Plasma samples were obtained after signed consent, from blood donors voluntarily assisting to the Hemotherapy Department at Hospital de Clínicas in Montevideo, Uruguay. To assure consistency LDL was obtained from plasma of male donors 18 to 45 years old. The protocol was approved by the Ethics Committee of the hospital.

### Oxidation of LDL

The isolated LDL fraction was oxidized as described before, with minor modifications [27,106]. Briefly, the isolated LDL fraction was dialyzed against phosphate buffered saline (PBS, 137 mM NaCl, 2.7 mM KCl, 10 mM Na_2_HPO_4_, 1.8 mM KH_2_PO_4_, pH 7.4) with 20 μM CuSO_4_ at 25° C for 4 h, protected from light. Oxidation was terminated by changing the dialysis buffer to PBS with 1 mM DTPA for 16 h at 4° C, with two changes of buffer. Soon after, the oxLDL was bubbled with argon to remove oxygen. The level of lipid (TBARS and conjugated dienes) and protein (increased negative charges) oxidation were evaluated by spectrometry and electrophoresis, respectively (**S10 Fig**).

**Fluorescent labeling of LDL.** LDL and oxLDL, in the absence of oxygen, were labeled with the lipophilic fluorescent probe (2Z)-2-[(E)-3-(3,3-dimethyl-1-octadecylindol-1-yl-2-yl) prop-2-enylidene]-3,3-dimethyl-1-octadecylindole (DiI, AnaSpec, Fermont, CA), by mixing 50 μL of DiI (3 mg mL^-1^ in DMSO) per mg of LDL protein for 18 h in the dark at 37° C. After the incubation, the lipoprotein was centrifuged at 22,000 g for 1 h at 4° C to remove the excess of unbound probe and dialyzed against PBS with 1 mM DTPA. Protein concentrations were evaluated at 280 nm (ε = 1 (cm mg mL^-1^)^-1^). Samples were sterilized by filtration and stored at 4° C, in the dark for a maximum of 3 weeks in sealed vials bubbled with argon.

## Conjugated dienes

The experimental procedure and data processing were as described previously [27], with some modifications. Briefly, human LDL (0.1 mg mL^-1^) were challenged with 50 μM CuSO_4_ in the absence and the presence of increasing concentrations of the cannabis extracts (0.5–3.5 μg mL^-1^). The formation of conjugated dienes was followed at 234 nm using a Varioskan Flash plate reader (Thermo, Finland). The initial part of the latency period was employed to estimate the antioxidant capacity (AC μg^-1^). The protection, reflexing the capacity of the extracts to interrupt the lipoperoxidation chain of radical reactions, was calculated as the ratio between the slopes of the steep growing part of the graph obtained in the absence and the presence of the different agonists, as described previously in detail [27].

## Cell culture

The murine macrophage-like cell line J774.1 (ATCC-TIB-67, American Type Culture Collection) was maintained by passage in Dulbecco’s Modified Eagle Medium (DMEM) high glucose (Gibco, Invitrogen) containing glutamine (4 mM), sodium pyruvate (110 mg L^-1^), glucose (4.5 g L^-1^), penicillin (100 U mL^-1^), streptomycin (100 mg L^-1^), and heat-inactivated fetal bovine serum (10%). The cells were plated and incubated at 37° C in 5% CO_2_, as described [107].

## Microscopy analysis of foam cells

J774.1 cells (5x10^3^ cell mL^-1^) were seeded and grown on Teflon-printed glass slides for 48 hours. After reaching semiconfluency, the cells were incubated in the absence and the presence of increasing concentrations (5–20 μg mL^-1^) of the extracts, after one hour of incubation DiI-oxLDL (10 μg mL^-1^) was added and incubated for 4 hours in the presence of 5% CO_2_ at 37° C. Finally, the cells were washed twice with PBS-BSA 5% and once with PBS to remove the unbound ligand, then fixed with 2.5% paraformaldehyde in PHEM buffer (60 mM PIPES, 25 mM HEPES, 10 mM EGTA, and 2 mM MgCl_2_, pH 7.2), at 4°C for 10 min, followed by a permeabilization step with PHEM containing 0.1% Triton for 10 min. Consecutively the nuclei were counter-stained with DAPI and the cells were washed with PHEM two more times. A LSM ZEISS 800 scanning laser confocal microscope (SLMC, Zeiss Microscopy, Germany) was used (TRIC 700 nm, DAPI 535 nm) and the analysis of the signal was performed with the software ZEN Blue 2.3 (Zeiss Microscopy, Germany). The images were generated from stacks of ten focal planes of 0.25 μm thick and one hundred cells from eight different fields were evaluated in each condition. The fluorescence intensity was normalized with the cytoplasmic area (I/A). The results were expressed as a percentage of the I/A obtained with the treatments with respect to the control incubated without extract.

## Quantitation of oxLDL endocytosis

A quantitative method to evaluate the intracellular capture of DiI-oxLDL was developed by a modification of the already published technique [108]. J774.1 cells (2.5x10^5^ cell mL^-1^) were incubated in 6 well plates with increasing concentrations of the cannabis extracts and DiI-oxLDL (10 μg mL^-1^) for 24 h at 37°C, 5% CO_2_. Immediately after, the cells were washed with PBS, then PBS pH 2 and PBS again, to remove the uninternalized DiI-oxLDL complex. The cells were scrapped and collected in PBS-1% triton, centrifuged two times at 22.000 g for 1h at 4°C to obtain a clear supernatant. Protein concentration was determined with the bicinchoninic acid kit (Merck KGaA), using the Varioskan Flash plate reader at 562 nm. Fluorescence was measured in a FP-8200 spectrofluorometer (JASCO). The excitation and emission wavelength were selected from the UV-Vis absorption and fluorescent emission spectra, respectively (**S2 Fig**). The selected wavelengths (λ_exc_ 540 nm, λ_em_ 564 nm) gave a good correlation between the fluorescent signal and the concentration of DiI-oxLDL (**S2 Fig**). The fluorescent signal from the cells challenged with oxLDL and the agonists or extracts was corrected for protein content and normalized to the condition with vehicle alone (0.2% DMSO). The log (inhibitor) vs. normalized response (Eq 1) was used to determine the effective dose of the inhibitor to achieve 50% of inhibition (ED_50_).


$$DiIoxLDL\;uptake(\%)=\frac{100}{1+10^{\left((log{ED}_{50}-x)Hill\;slope\right)}}$$ Eq 1

## Evaluation of cellular toxicity of cannabinoid extracts

Cell viability was determined by the mitochondrial-dependent reduction of 3-[4,5-dimethylthiazole-2-yl]-2,5-diphenyltetrazolium bromide (MTT, Merck KGaA) to formazan [31]. Semiconfluent macrophages were incubated in DMEM with increasing concentrations of the cannabinoid extracts (1–40 μg mL^-1^), phytocannabinoids (3–30 μM), AEA (0.001–125 μM), or fixed concentration of cannabinoid receptor agonists and antagonists at the higher concentrations explored for 24 h at 37° C, 5% CO_2_. A control condition with vehicle alone (0.2% DMSO) was also added to each experiment. The medium was replaced with DMEM containing MTT (0.1 mg mL^-1^) and the cells were incubated at 37° C for 4 h. After removing the media, formazan crystals were dissolved in DMSO, and the absorbance was registered using the Varioskan plate reader at 560 nm. The results were normalized with the vehicle-treated condition and fitted to log dose-normalized response as described before to determine the lethal dose 50% (LD_50_). Therapeutic indexes (TI) were calculated as the ratios between the ED_50_ and LD_50_.

## Western blots

The J774.1 cells were growth in 6 well plates until semi confluence. The plates with cells were placed on ice and washed 3 times with cold PBS. The cells were then lifted in PBS with cell rakes, centrifuged at 200 g, and resuspended in a lysis buffer, composed as follows: 50 mM Tris-HCl (pH = 7.2); 100 mM NaCl; 1 mM EDTA; TX-100 1% and the SIGMAFAST® protease inhibitor cocktail was added prior to lysing the cells. In the case of the analysis of phosphorylated proteins, 1 mM EGTA was used instead of EDTA and 0.5 mM Na_3_VO_4_ and 2.5 mM Na_4_P_2_O_7_ were added to the lysis solution. The protein content of the cell lysates was determined using the BCA protein assay kit (Merck). The proteins were separated in 12% SDS-PAGE and immobilized on PVDF membranes (Thermo Fisher) at 100 V for 1 h. After blocking with 5% skim milk for 1 h, the membranes were incubated with the corresponding primary antibody diluted 1:500–1,000 in the blocking buffer for one hour. The following primary antibodies were used: rabbit anti-CD204/SR-A1 (ABCAM), mouse anti-CD36 (Antibodies, UK), rabbit anti-OLR1 (Antibodies, UK), mouse anti-phospho-IκBα, rabbit anti-IκBα, rabbit anti-phospho(Ser536)-p65 (cat#3033) and rabbit anti-p65 (Cell Signaling Technology), mouse anti-β-actin (Sigma). After washing the membranes were incubated for 1 h with the following secondary antibodies (1:5,000): goat anti-rabbit IRDye 680 and goat anti-mouse IRDye 800 CW (Li-Cor Inc.). The fluorescent signal was recorded in a G-Box (Syngene, Synoptics Ltd), and the densitometric analysis of the signals were performed using ImageJ software (U.S. NIH). The densitometric values obtained were normalized by their corresponding actin values. In the case of phospho-IκBα and phospho-p65, the densitometric values obtained were normalized by their corresponding total IκBα or p65 values. The comparisons of normalized densitometric values between treatments were analyzed by one-way ANOVA, as described in the Statistical analysis section.

## Statistical analysis

Data were analyzed using GraphPad Prism 6.0 (Graph-Pad Software, La Jolla, CA). Correlations were analyzed by linear regression. Data are representative or were expressed as mean ± standard deviation from at least three independent experiments. Statistical analyses were performed by one-way analysis of variance (ANOVA), followed by Dunnett’s multiple comparison test. Differences with p < 0.05 were considered statistically significant.

## Supporting information

S1 Fig Relationship between the antioxidant effect of the extracts and their cannabinoid content. A. Antioxidant capacity is proportional to total cannabinoid content (R^2^ = 0.7315). B. Relationship between Antioxidant capacity and relative Δ^9^THC content (R^2^ = 0.9984). The red diamond corresponds to the value of 100% Δ^9^THC and its experimentally determined AC value. Note how it falls on the linear fit for the 3 extracts. The inset zooms in the extracts’ values. C. Linear relationship between the IC50 for the propagation phase slope and the relative Δ^9^THC content (R^2^ = 0.9233).
(TIFF)

S2 Fig Spectroscopic analysis of DiI-oxLDL. **A**. Absorbance spectra of DiI-oxLDL at 0.2 mg/mL (solid line) and oxLDL without the fluorescent label (dashed line). **B**. Fluorescence emission spectrum of DiI-oxLDL (8.5 μg/mL) with excitation wavelength at 540 nm. **C**. Linear fit (122 ± 4 URF/(μg/mL), R^2^ = 0.94) between fluorescence intensity (λex = 540 nm, λem = 564 nm) at increasing concentrations of DiI-oxLDL (1–10 μg/mL). Each point represents the average of three independent experiments.
(TIFF)

S3 Fig Synergistic effect of cannabis extracts compared to individual cannabinoid components. Experimental response and theoretical component cannabinoid additive response graphed for extracts E1 (A). E3 (B), E3 (C). The difference between the experimental and the additive response for each extract was plotted (D) and the maximal synergistic effect calculated for each extract as the top asymptote of each extract’s sigmoidal fit.
(TIFF)

S4 Fig Effect of cannabinoids on the viability of macrophages J774.1. Mitochondrial activity, represented as formazan accumulation (absorbance at 562 nm), was evaluated in J774.1 cells incubated for 24 hours with increasing concentrations of E1 (1–40 μg/mL) (**A**), E2 (1–40 μg/mL) (**B**), and E3 (1–40 μg/mL) (**C**), AEA (0.001–125 μM) (**D**), THCA (**E**), THC (**F**), CBDA (**G**) and CBD (**H**). The results of three independent experiments are expressed as a percentage of the signal respect to vehicle. Dose-response fits were used to determine LD_50_ shown in Table 2.
(TIFF)

S5 Fig Effect of CB1 and CB2 antagonists on LDLmox Internalization in J774.1 macrophages at 6h incubation. The cells were incubated for 6 h with oxLDL (10 μg mL-1) without (-) or with E1 (2 μg mL-1), E2 (5 μg mL-1) and E3 (5μg mL-1), in the absence (-) and the presence of the antagonists of the CB1 (AM251, 5 μM) and CB2 (AM630, 5 μM). The results are presented as mean and SD from three independent experiments. No differences were found by One-way ANOVA followed by Dunnett’s multiple comparisons test comparing the condition with the extract alone and the same extract plus antagonist.
(TIFF)

S6 Fig Effect of endocannabinoid system agonists at 24 h on LDLmox Internalization in J774.1 macrophages. The cells were incubated for 24 h with LDLmox-DiI (10 μg/mL), in addition to extracts E1 (2 μg/mL), E2 and E3 (5 μg/mL) and the SEC agonists: ML (10 μM) (**A**), Cap (50 μM) (**B**) and GSK (**C**) (10 μM). Results are expressed as a percentage of a vehicle alone. The averages of three independent experiments and their standard deviations are shown.
(TIFF)

S7 Fig Effect of endocannabinoid system agonists and antagonists on the viability of J774.1 macrophages. J774.1 cells were incubated for 24 hours with the maximum concentration used of each compound and the cell viability determined by the MTT method. The results of three independent experiments are expressed as a percentage of the vehicle for each respective treatment.
(TIFF)

S8 Fig Effect of Ca^2+^ on foam cell formation. **A**. Semi-confluent J774 cells were incubated for 6 h in the absence or presence of DiI-oxLDL (10 μg/mL) plus BAPTA-AM (5 μM) and/or EGTA (4 mM). Live cells were observed by bright field (upper images) and fluorescence using the red channel of the ZOE Fluorescent Cell Imager. **B**. The cells were incubated with DiI-oxLDL and BAPTA-AM (5–20 μM), EGTA (4 mM) or a combination of both chelators. Internalized DiI-oxLDL fluorescence was assessed at λex = 540 and λem = 564 nm. Results from three independent experiments were normalized for protein concentration and are expressed as percentage of a control condition without chelators.
(TIFF)

S9 Fig Differential gene expression between J774 and murine BMDMs. A. Raw CPM expression data for CD36 in murine BMDMs and J774 cells from a high throughput sequencing expression profiling experiment GEO accession: GSE88801. B. Expression data was analyzed using iDEP.96 [109] as follows: Pre-processed using rlog for PCA and clustering. DEG was performed with DESeq2 with the following model: Expression ~CellType + Condition + Time + CellType:Condition + CellType:Time. Min fold change was set at 2 and the FDR cutoff set at 0.05 CD36 expression in BMDMs was 160,450 times higher than in J774.
(TIFF)

S10 Fig Characterization of LDL oxidation. **A**. MDA content was determined by TBARS, and the values normalized by the concentration of ApoB100 in LDL. **B**. Native electrophoresis in 0.5% agarose of LDL and oxidized LDL (10 μg), run at 90 V for 150 min. R.E.M = 1.1. **C**. UV spectra (230–300 nm) of (0.25 mg/mL). LDL (black) and LDL oxidized with Cu^2+^ (20 μM) for 4 h, 25°C (red).
(TIFF)

S11 Fig All western blot membranes. (PDF)

S1 Table All western blot membranes. Effect of cannabis extracts and phytocannabinoids on LDL oxidation.
(PDF)

S2 Table All western blot membranes. Binding affinities of cannabinoid receptors ligands assayed.
(PDF)

S1 File (ZIP)

## Abbreviations

AEA: N-arachidonoylethanolamine or anandamide
2-AG: 2-arachidonoylglycerol
CB1: cannabinoid receptor type 1
CB2: cannabinoid receptor type 2
CBC: cannabichromene
CBD: cannabidiol
CBDA: cannabidiolic acid
CBDV: cannabidivarin
CBG: cannabigerol
CBGA: cannabigerolic acid
CBN: cannabinol
CVD: cardiovascular disease
DMSO: dimethyl sulfoxide
GPR55: G protein-coupled receptor 55
IκB: Inhibitor of κB subunit
IKK: kinase of IκB
LDL: low density lipoprotein
oxLDL: oxidized LDL
NFκB: nuclear factor kappa-light-chain-enhancer of activated B cells
PRR: pattern recognition receptor
PKC: protein kinase C
SLCM: scanning laser confocal microscope
SR: scavenger receptor
THC: Δ^9^-tetrahydrocannabinol
Δ^8^THC: Δ^8-^tetrahydrocannabinol
THCA: Δ^9^-tetrahydrocannabinolic acid
TRPV1: transient receptor potential cation channel subfamily V member 1 or vanilloid receptor
TRPV4: transient receptor potential cation channel subfamily V member 4
UPLC-DAD: ultra performance liquid chromatography with diode-array detection
