# Supplementary figures and images for: Cannabis sativa extracts inhibit LDL oxidation and the formation of foam cells in vitro, acting as potential multi-step inhibitors of atherosclerosis development
Source: PLoS One. 2024 Dec 20;19(12):e0310777. doi: 10.1371/journal.pone.0310777 (PMC11661628; doi:10.1371/journal.pone.0310777)

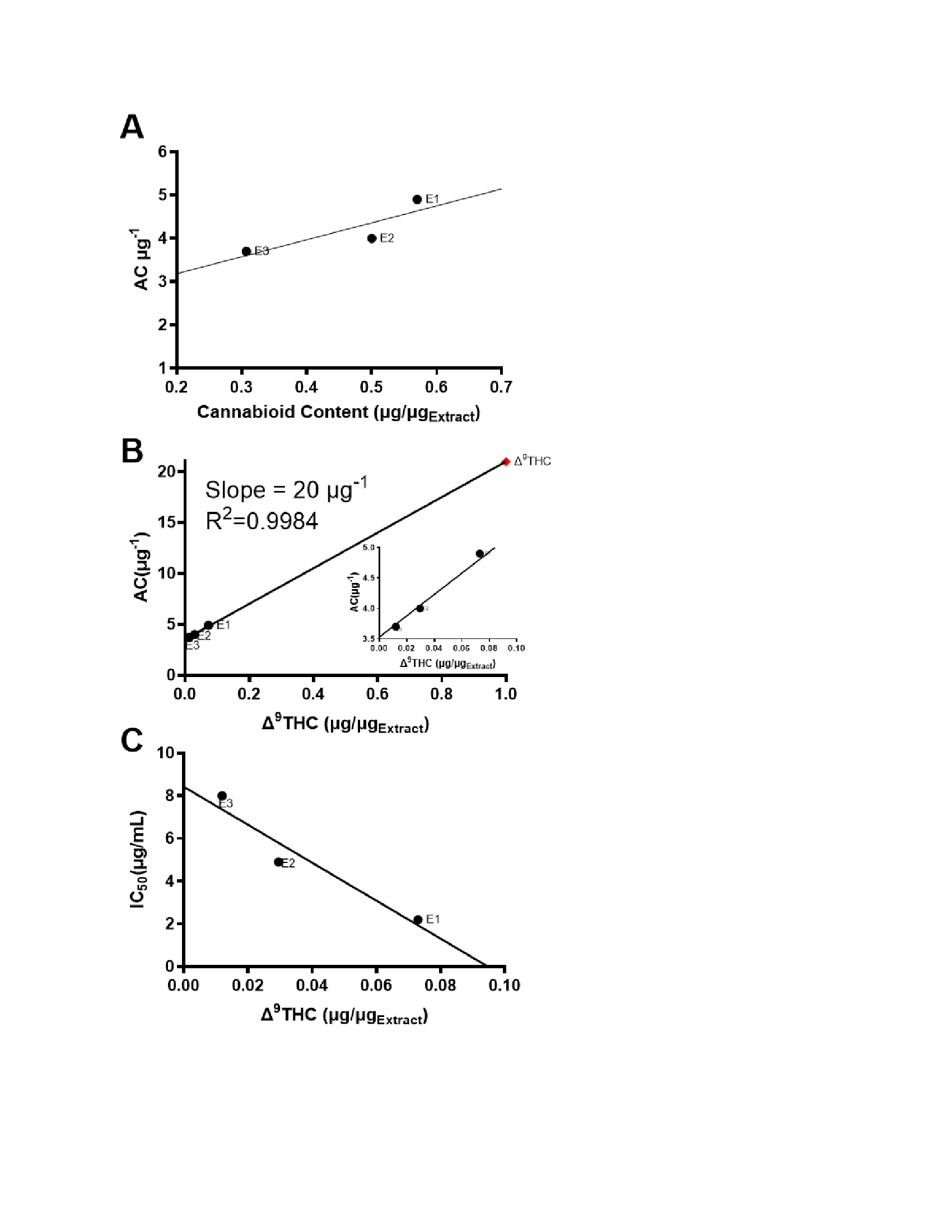

Supplement: S1 Fig — A. Antioxidant capacity is proportional to total cannabinoid content (R2 = 0.7315). B. Relationship between Antioxidant capacity and relative Δ9THC content (R2 = 0.9984). The red diamond corresponds to the value of 100% Δ9THC and its experimentally determined AC value. Note how it falls on the linear fit for the 3 extracts. The inset zooms in the extracts’ values. C. Linear relationship between the IC50 for the propagation phase slope and the relative Δ9THC content (R2 = 0.9233). (TIFF) [file pone.0310777.s001.tiff]

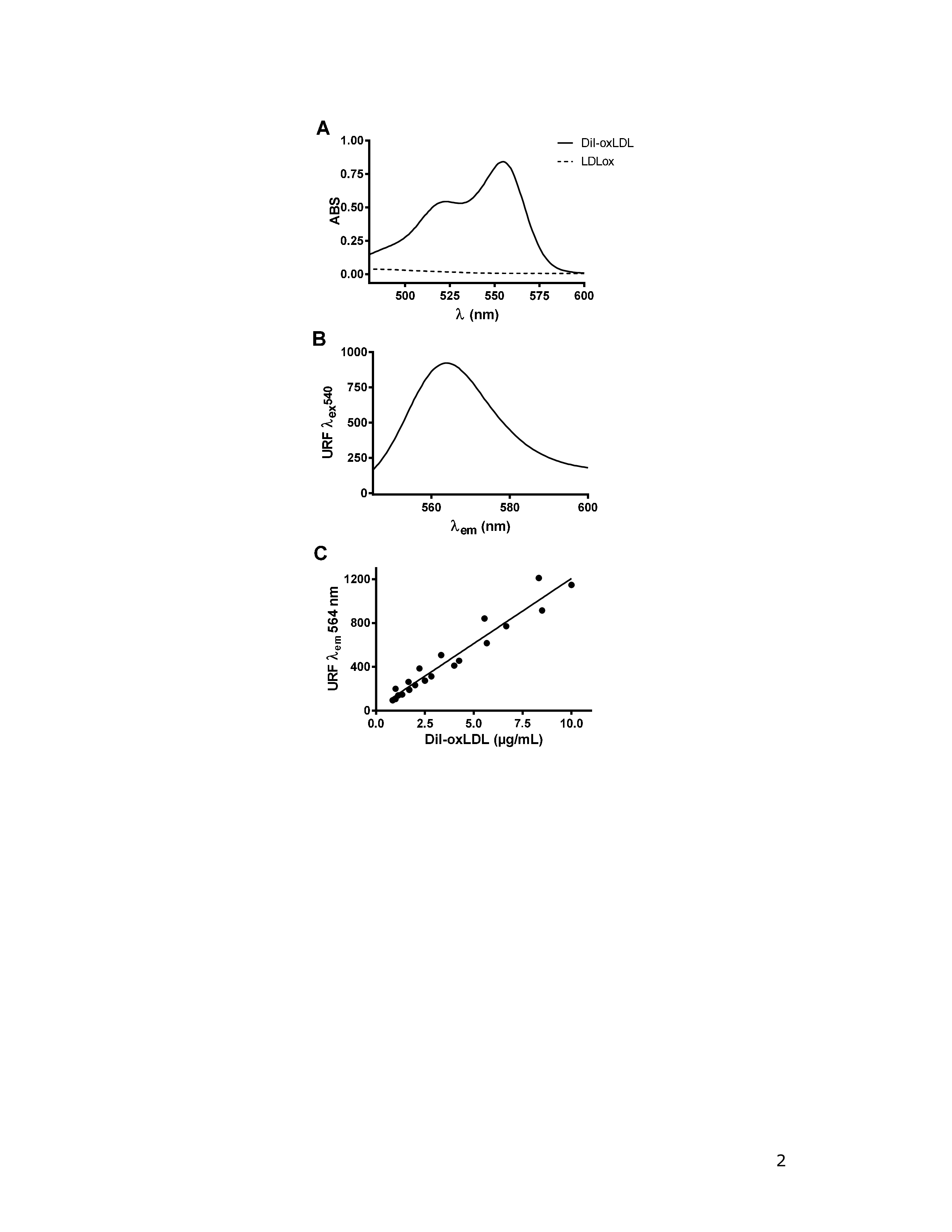

Supplement: S2 Fig — A. Absorbance spectra of DiI-oxLDL at 0.2 mg/mL (solid line) and oxLDL without the fluorescent label (dashed line). B. Fluorescence emission spectrum of DiI-oxLDL (8.5 μg/mL) with excitation wavelength at 540 nm. C. Linear fit (122 ± 4 URF/(μg/mL), R2 = 0.94) between fluorescence intensity (λex = 540 nm, λem = 564 nm) at increasing concentrations of DiI-oxLDL (1–10 μg/mL). Each point represents the average of three independent experiments. (TIFF) [file pone.0310777.s002.tiff]

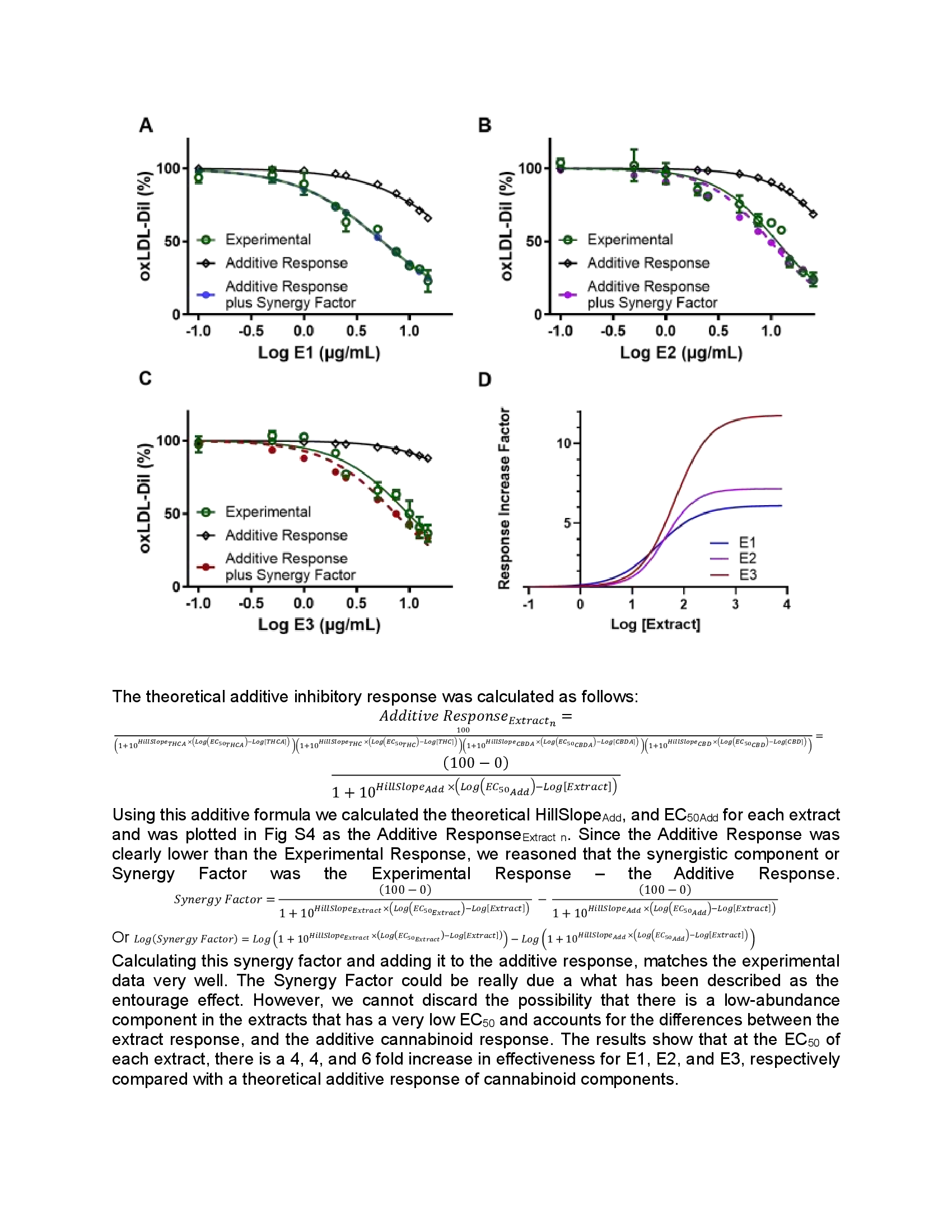

Supplement: S3 Fig — Experimental response and theoretical component cannabinoid additive response graphed for extracts E1 (A). E3 (B), E3 (C). The difference between the experimental and the additive response for each extract was plotted (D) and the maximal synergistic effect calculated for each extract as the top asymptote of each extract’s sigmoidal fit. (TIFF) [file pone.0310777.s003.tiff]

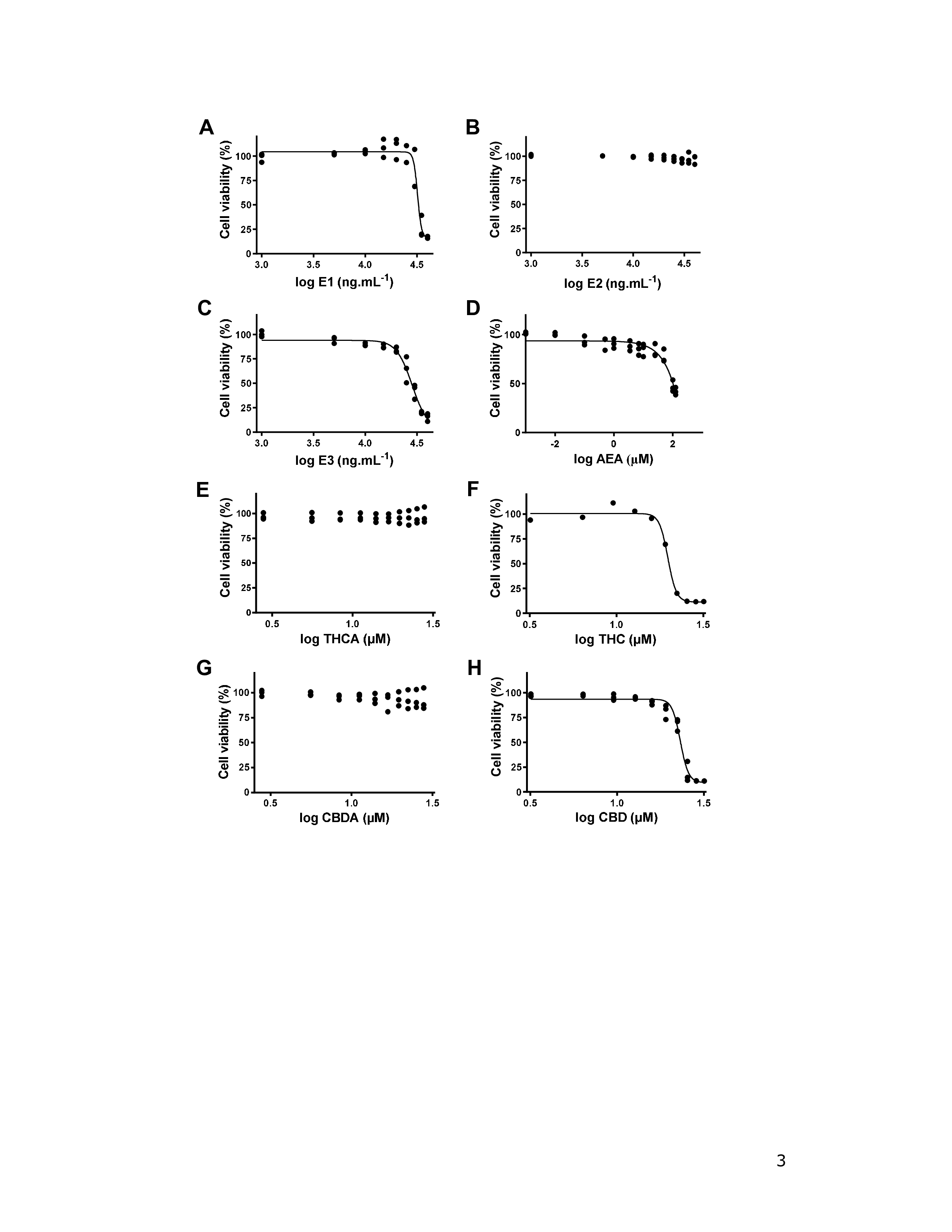

Supplement: S4 Fig — Mitochondrial activity, represented as formazan accumulation (absorbance at 562 nm), was evaluated in J774.1 cells incubated for 24 hours with increasing concentrations of E1 (1–40 μg/mL) (A), E2 (1–40 μg/mL) (B), and E3 (1–40 μg/mL) (C), AEA (0.001–125 μM) (D), THCA (E), THC (F), CBDA (G) and CBD (H). The results of three independent experiments are expressed as a percentage of the signal respect to vehicle. Dose-response fits were used to determine LD50 shown in Table 2. (TIFF) [file pone.0310777.s004.tiff]

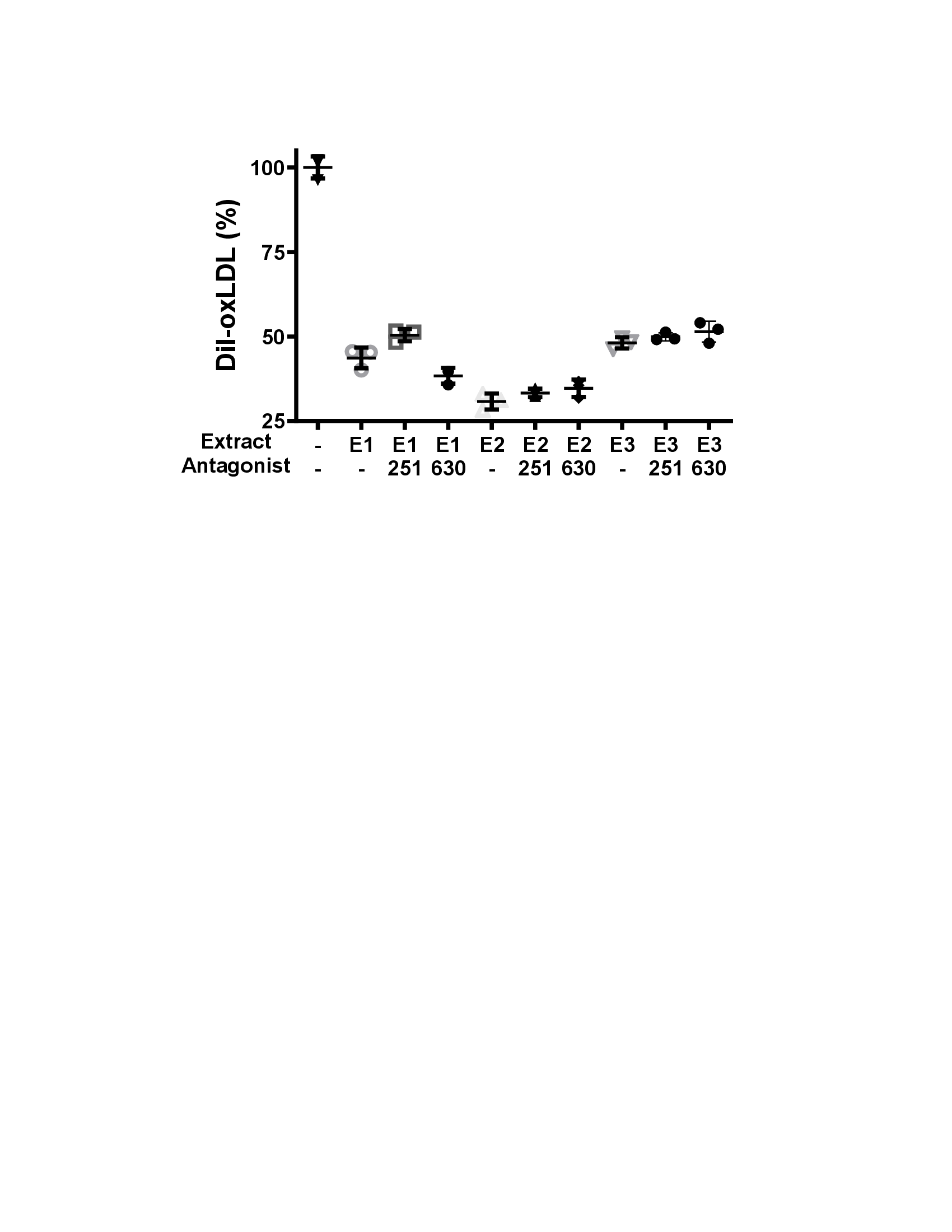

Supplement: S5 Fig — The cells were incubated for 6 h with oxLDL (10 μg mL-1) without (-) or with E1 (2 μg mL-1), E2 (5 μg mL-1) and E3 (5μg mL-1), in the absence (-) and the presence of the antagonists of the CB1 (AM251, 5 μM) and CB2 (AM630, 5 μM). The results are presented as mean and SD from three independent experiments. No differences were found by One-way ANOVA followed by Dunnett’s multiple comparisons test comparing the condition with the extract alone and the same extract plus antagonist. (TIFF) [file pone.0310777.s005.tiff]

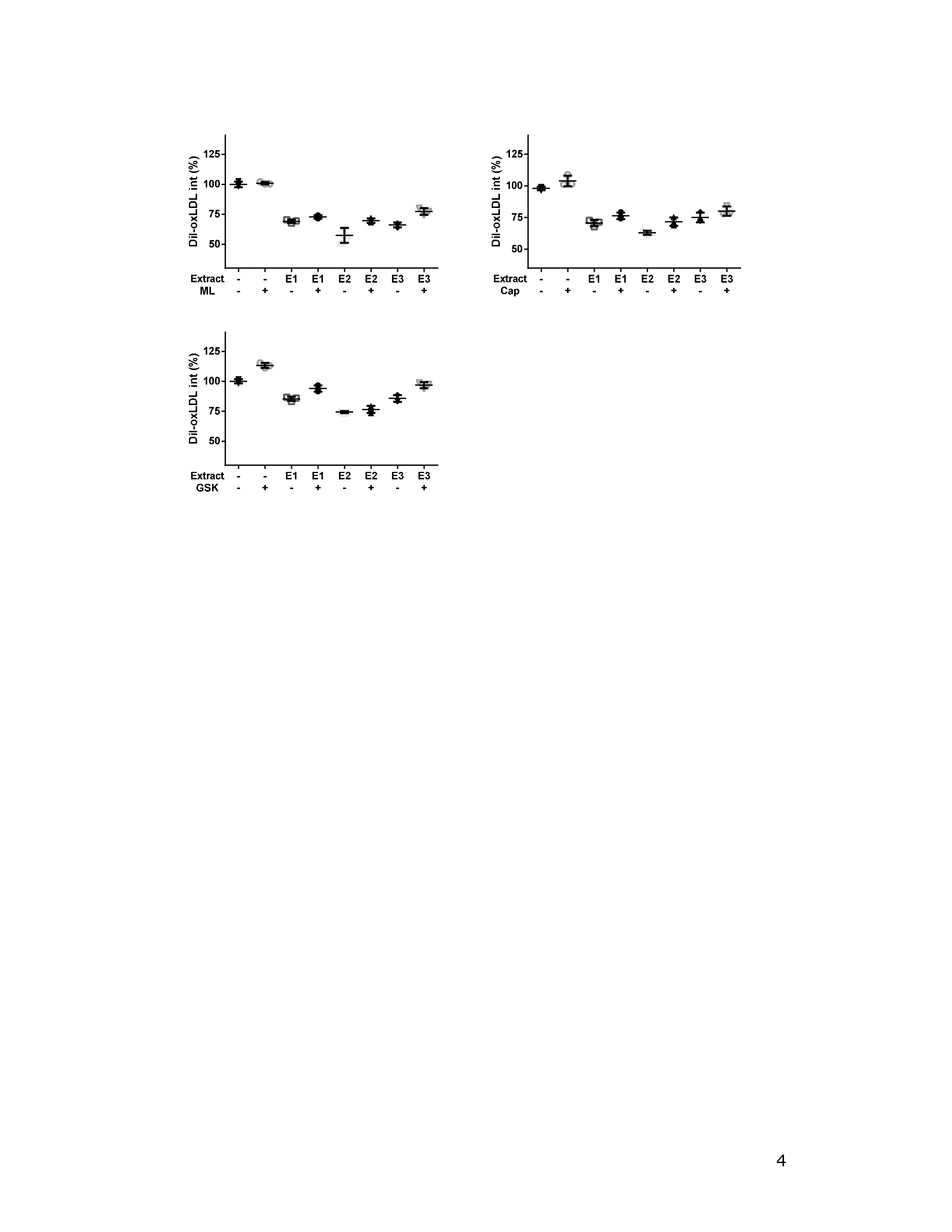

Supplement: S6 Fig — The cells were incubated for 24 h with LDLmox-DiI (10 μg/mL), in addition to extracts E1 (2 μg/mL), E2 and E3 (5 μg/mL) and the SEC agonists: ML (10 μM) (A), Cap (50 μM) (B) and GSK (C) (10 μM). Results are expressed as a percentage of a vehicle alone. The averages of three independent experiments and their standard deviations are shown. (TIFF) [file pone.0310777.s006.tiff]

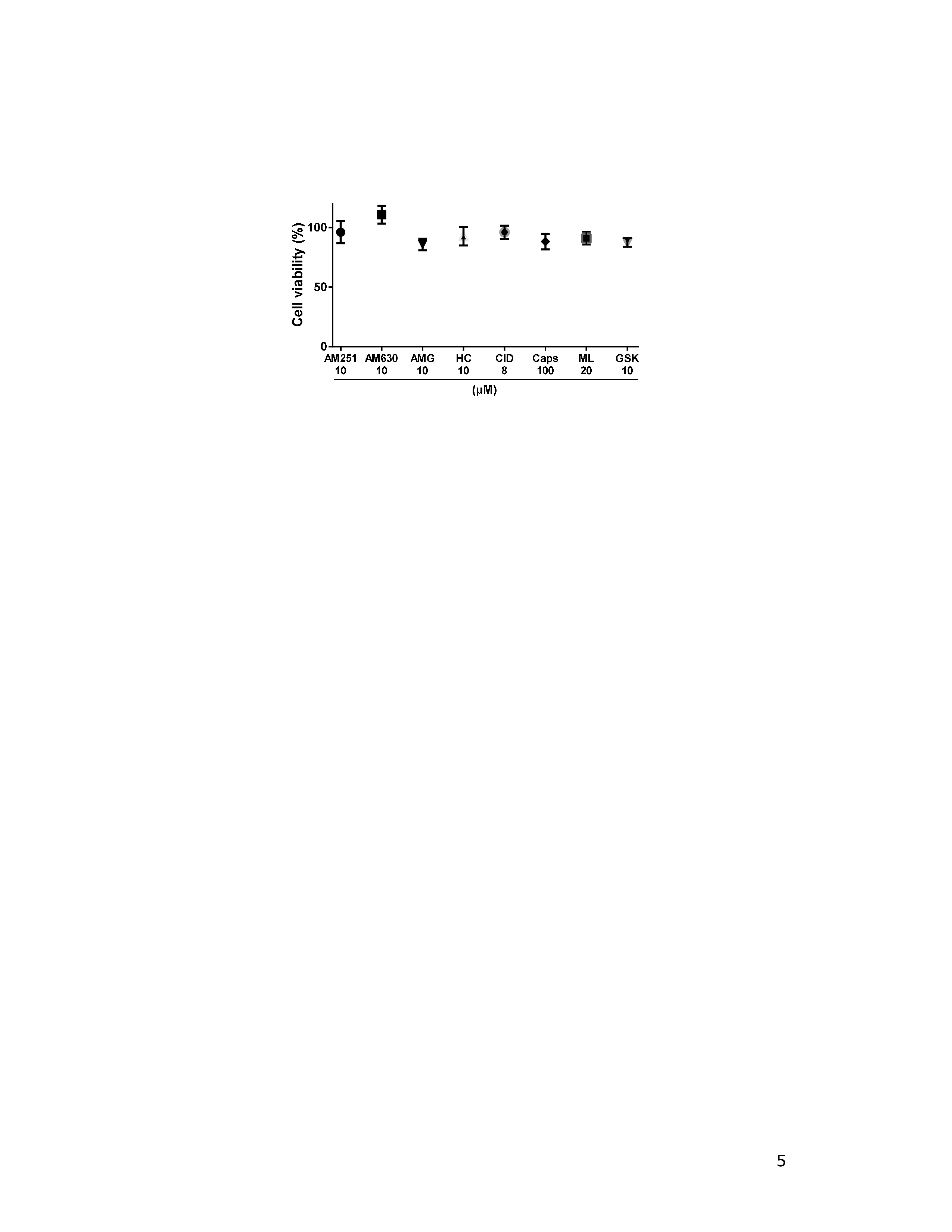

Supplement: S7 Fig — J774.1 cells were incubated for 24 hours with the maximum concentration used of each compound and the cell viability determined by the MTT method. The results of three independent experiments are expressed as a percentage of the vehicle for each respective treatment. (TIFF) [file pone.0310777.s007.tiff]

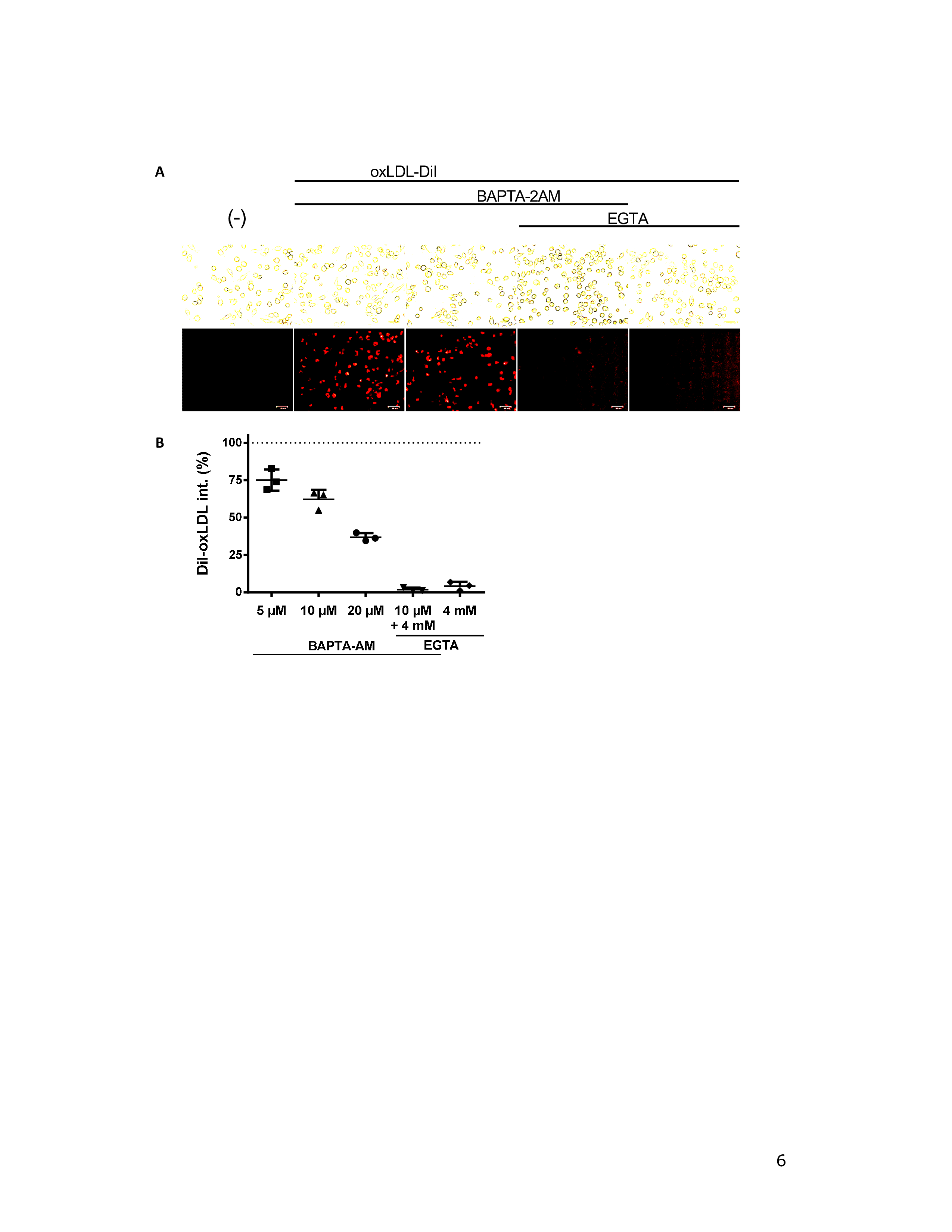

Supplement: S8 Fig — A. Semi-confluent J774 cells were incubated for 6 h in the absence or presence of DiI-oxLDL (10 μg/mL) plus BAPTA-AM (5 μM) and/or EGTA (4 mM). Live cells were observed by bright field (upper images) and fluorescence using the red channel of the ZOE Fluorescent Cell Imager. B. The cells were incubated with DiI-oxLDL and BAPTA-AM (5–20 μM), EGTA (4 mM) or a combination of both chelators. Internalized DiI-oxLDL fluorescence was assessed at λex = 540 and λem = 564 nm. Results from three independent experiments were normalized for protein concentration and are expressed as percentage of a control condition without chelators. (TIFF) [file pone.0310777.s008.tiff]

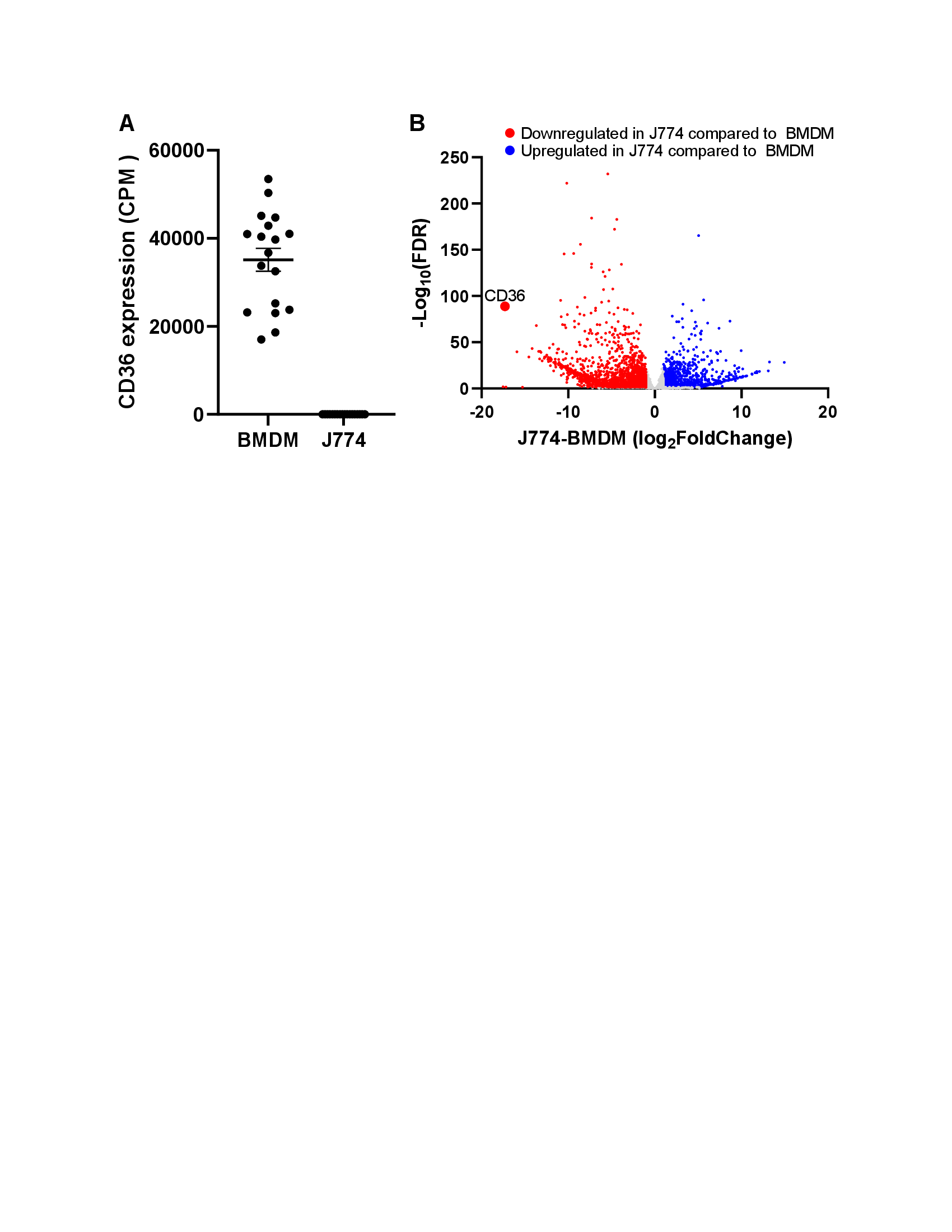

Supplement: S9 Fig — A. Raw CPM expression data for CD36 in murine BMDMs and J774 cells from a high throughput sequencing expression profiling experiment GEO accession: GSE88801. B. Expression data was analyzed using iDEP.96 [109] as follows: Pre-processed using rlog for PCA and clustering. DEG was performed with DESeq2 with the following model: Expression ~CellType + Condition + Time + CellType:Condition + CellType:Time. Min fold change was set at 2 and the FDR cutoff set at 0.05 CD36 expression in BMDMs was 160,450 times higher than in J774. (TIFF) [file pone.0310777.s009.tiff]

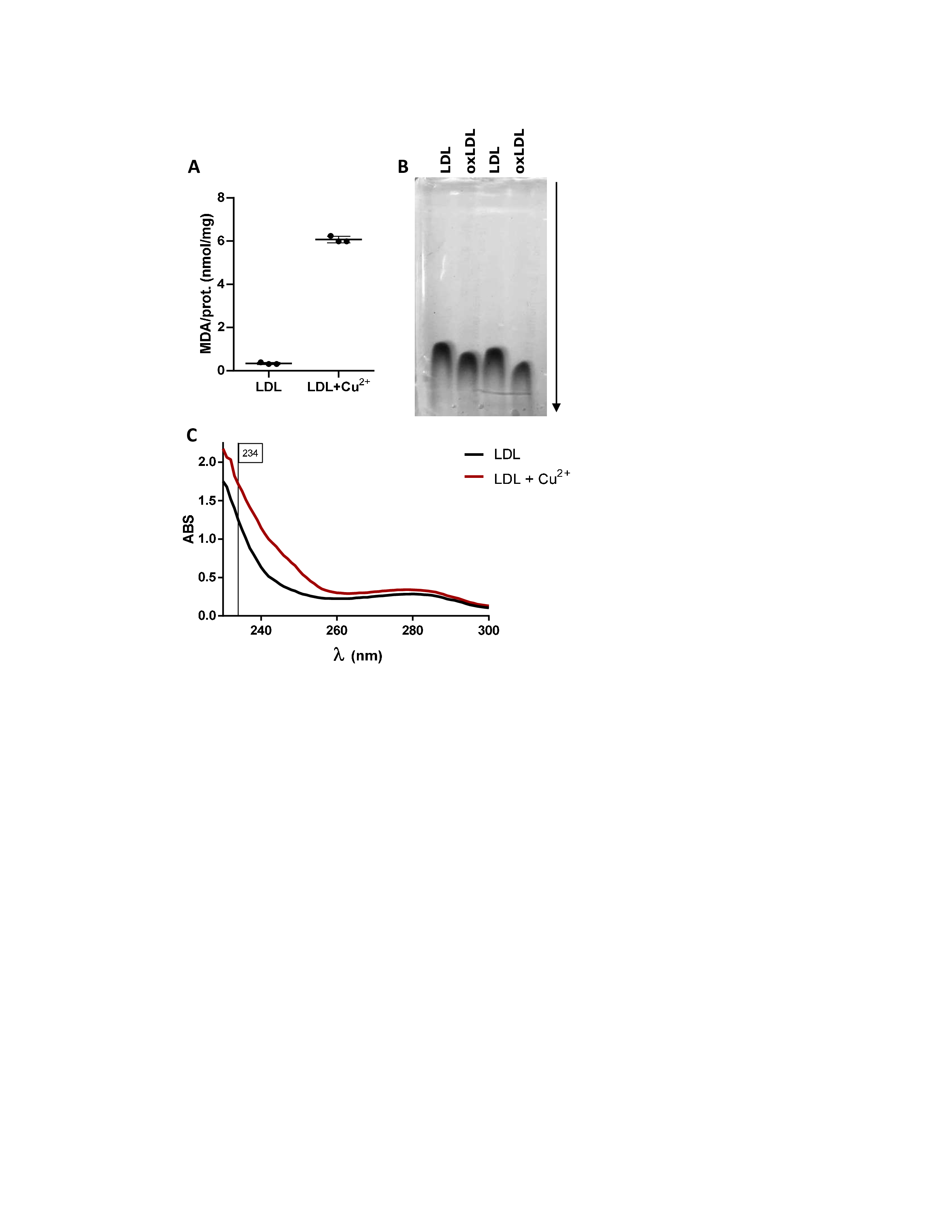

Supplement: S10 Fig — A. MDA content was determined by TBARS, and the values normalized by the concentration of ApoB100 in LDL. B. Native electrophoresis in 0.5% agarose of LDL and oxidized LDL (10 μg), run at 90 V for 150 min. R.E.M = 1.1. C. UV spectra (230–300 nm) of (0.25 mg/mL). LDL (black) and LDL oxidized with Cu2+ (20 μM) for 4 h, 25°C (red). (TIFF) [file pone.0310777.s010.tiff]
