# Supplementary material for: Cannabis sativa extracts inhibit LDL oxidation and the formation of foam cells in vitro, acting as potential multi-step inhibitors of atherosclerosis development
Source: PLoS One. 2024 Dec 20;19(12):e0310777. doi: 10.1371/journal.pone.0310777 (PMC11661628; doi:10.1371/journal.pone.0310777)
Supplement: S11 Fig — (PDF) [file pone.0310777.s011.pdf]

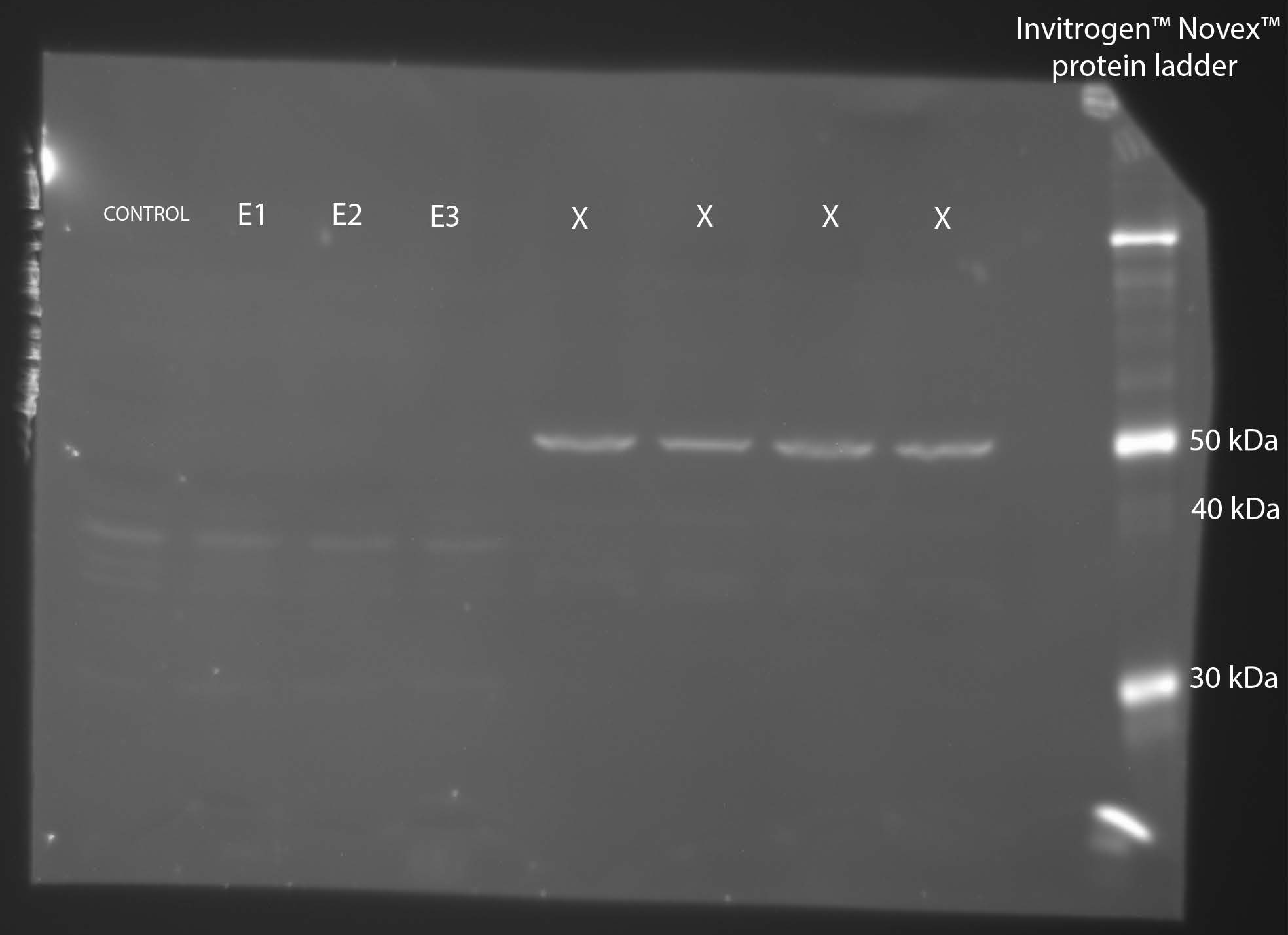
Invitrogen™ Novex™
protein ladder
CONTROL
E1
E2
E3
X
X
X
X
50 kDa
40 kDa
30 kDa

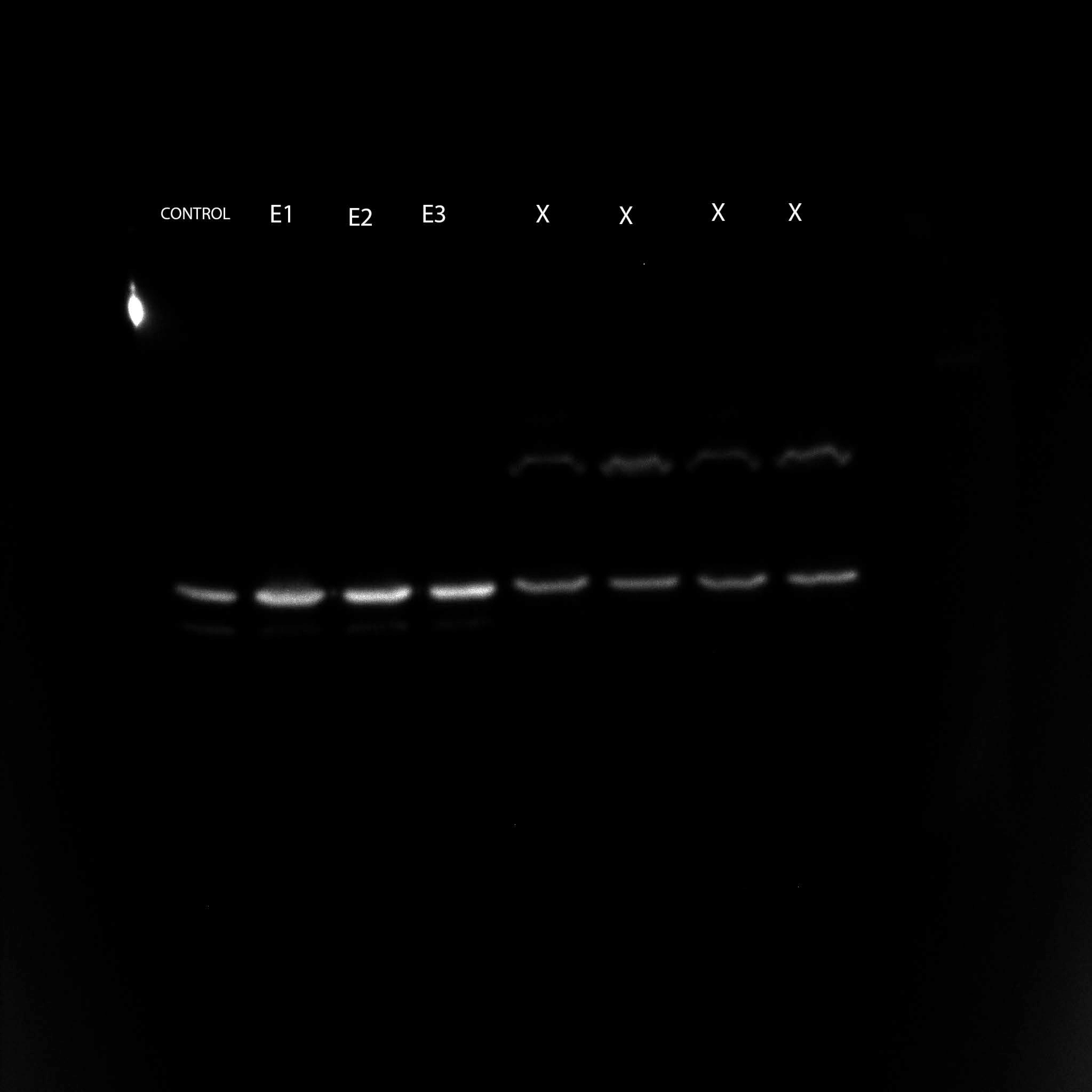
CONTROL
E1
E2
E3
X
X
X
X

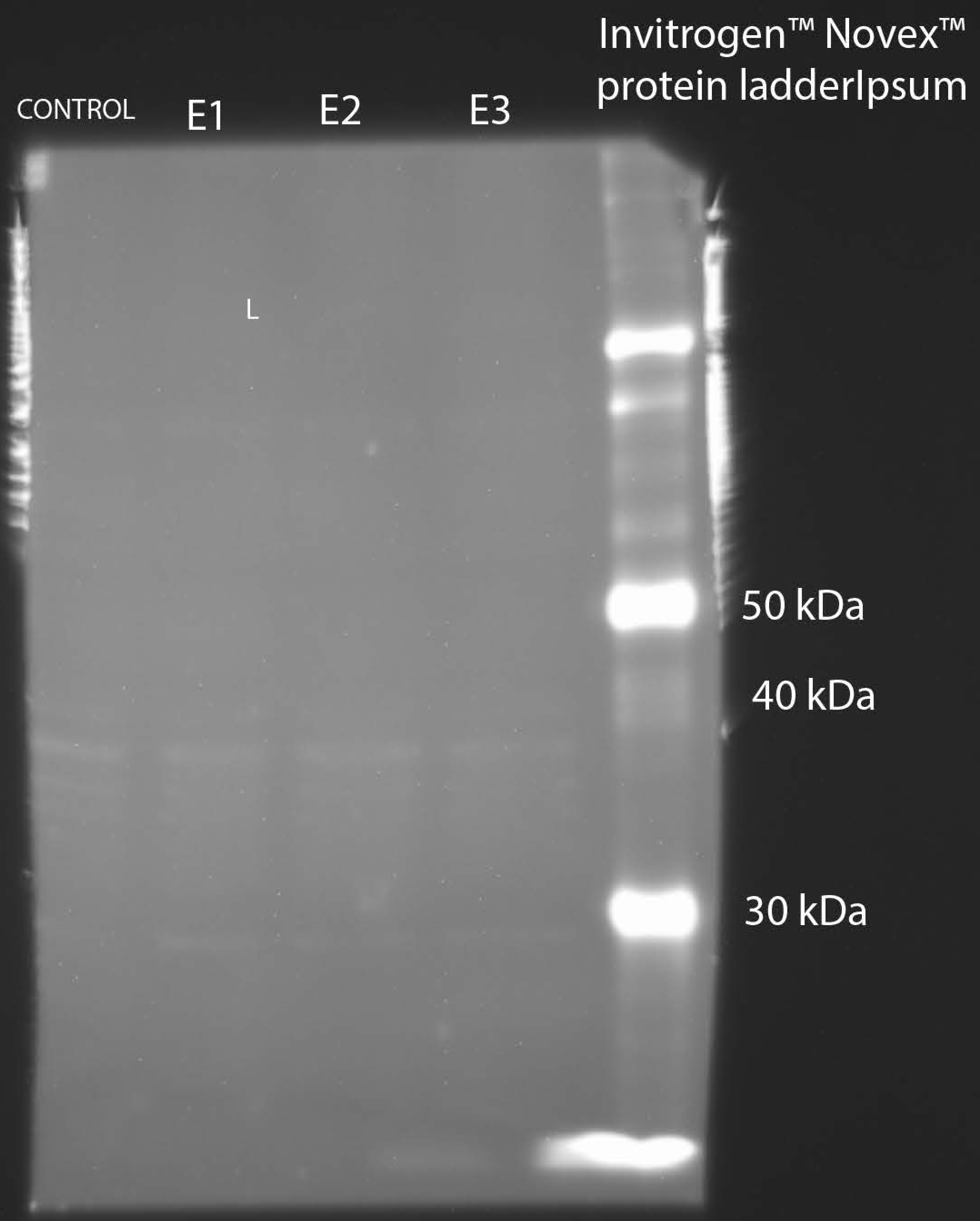
Invitrogen™ Novex™
protein ladderIpsum
CONTROL
E1
E2
E3
L
50 kDa
40 kDa
30 kDa

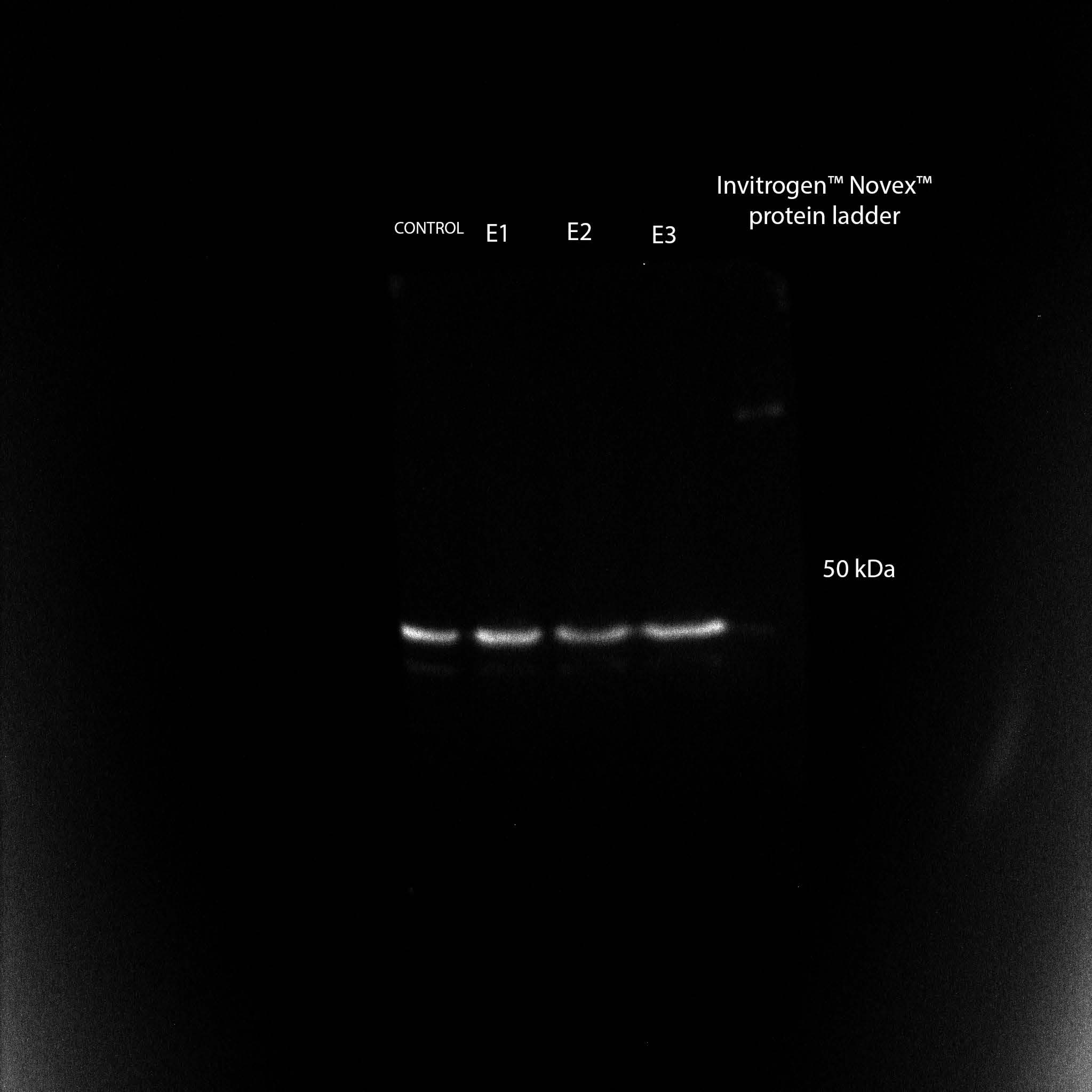

Invitrogen™ Novex™
protein ladder
CONTROL
E1
E2
E3
50 kDa

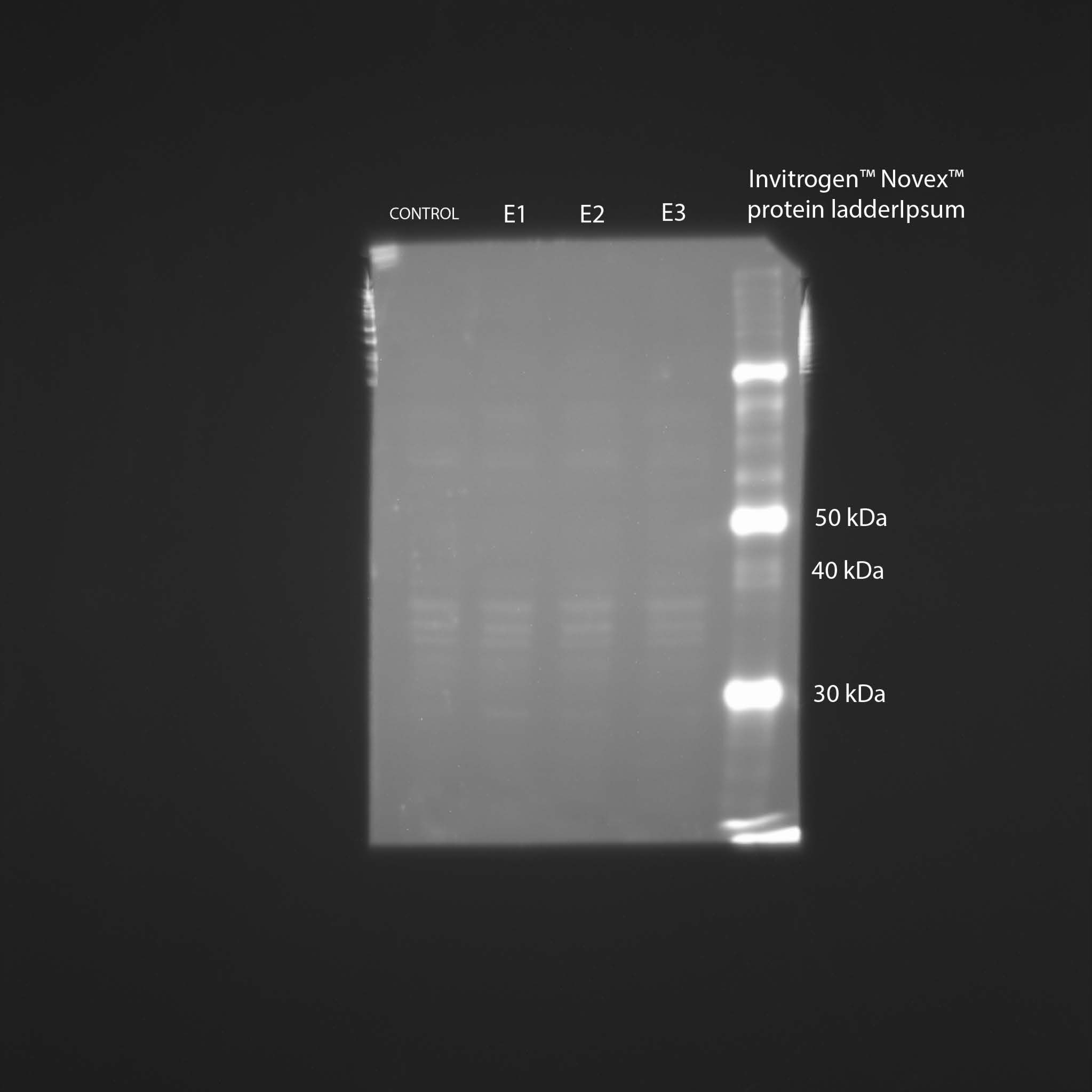
CONTROL
E1
E2
E3
Invitrogen™ Novex™
protein ladderIpsum
50 kDa
40 kDa
30 kDa

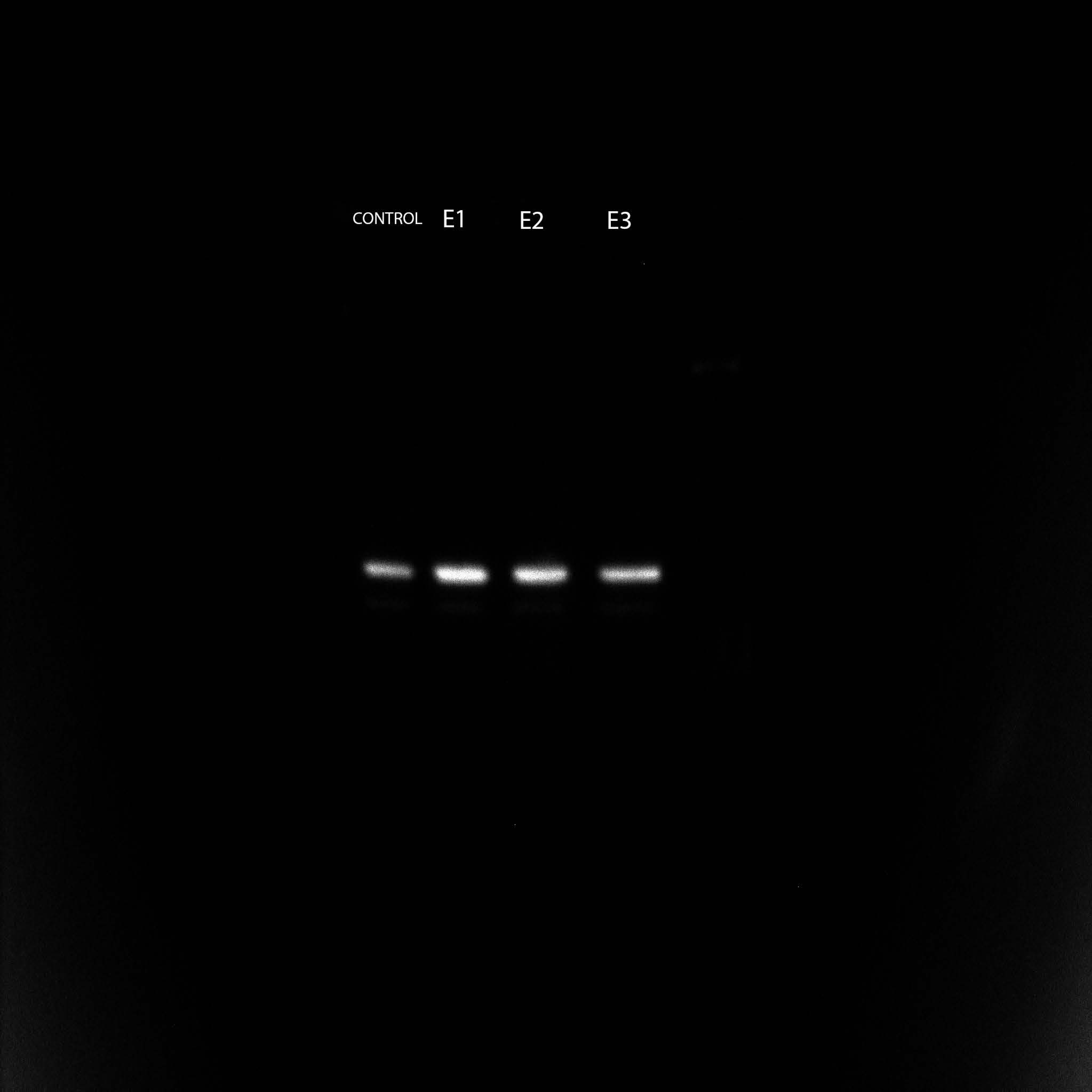
CONTROL
E1
E2
E3

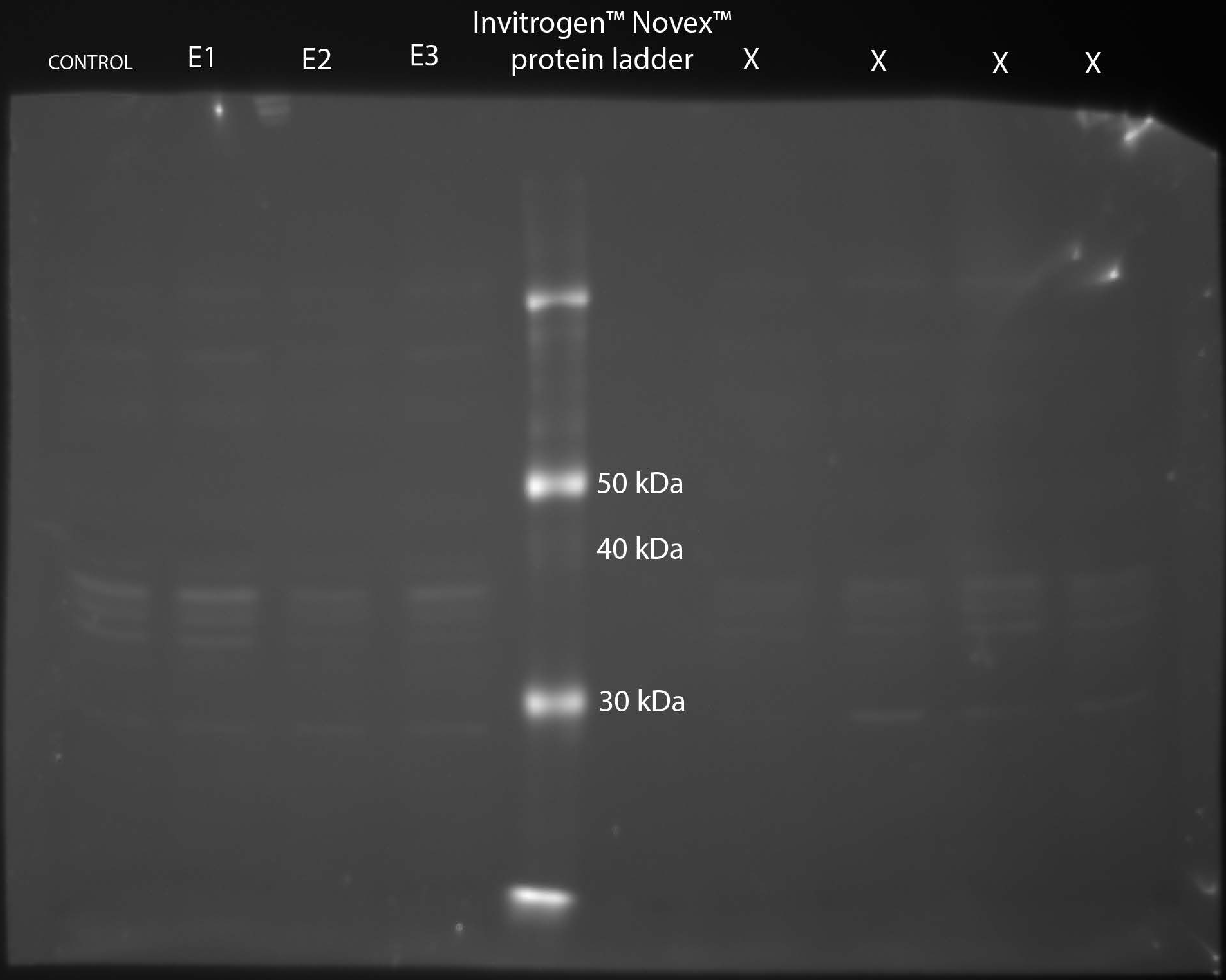
CONTROL
E1
E2
E3
Invitrogen™ Novex™ protein ladder
X
X
X
X
50 kDa
40 kDa
30 kDa

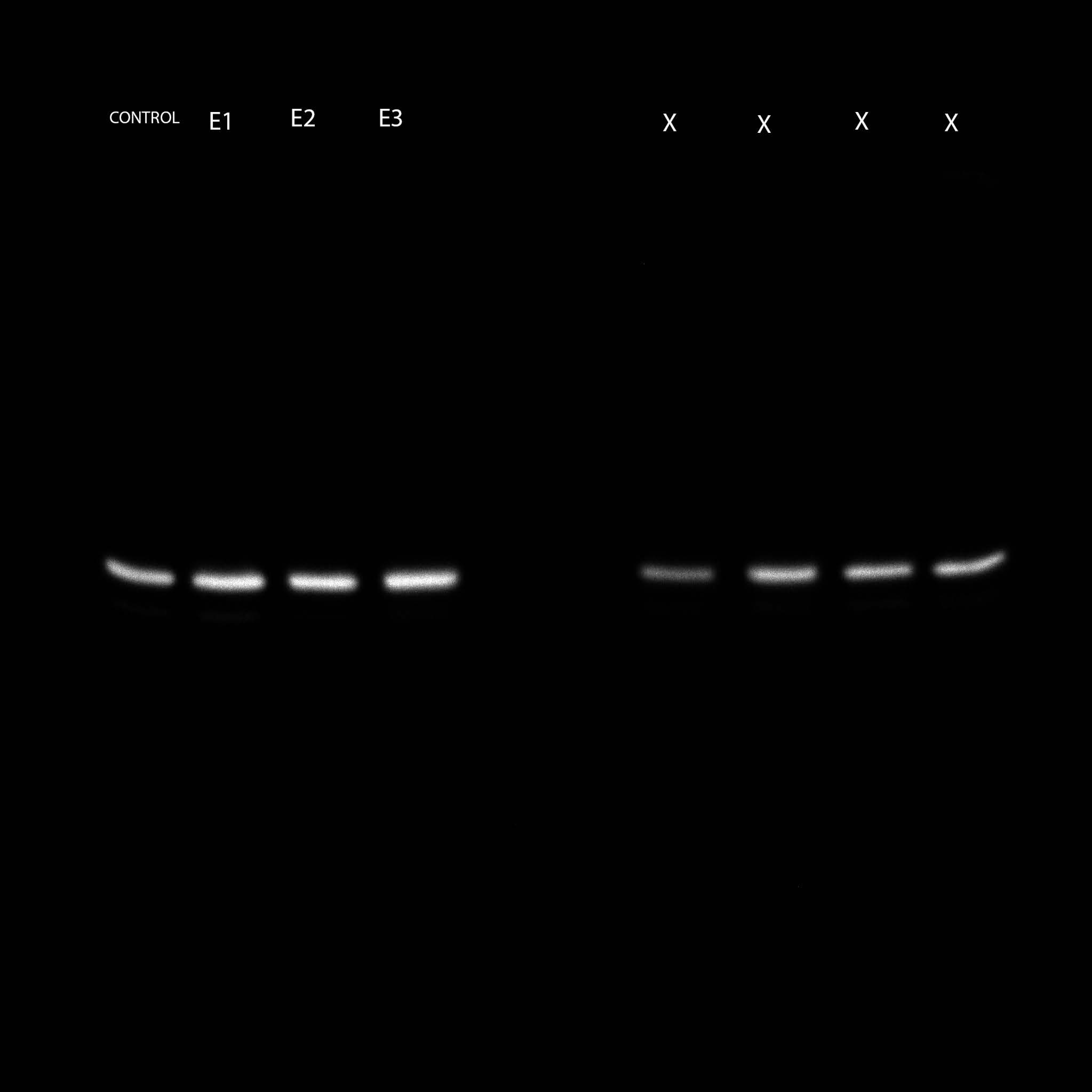
CONTROL
E1
E2
E3
X
X
X
X

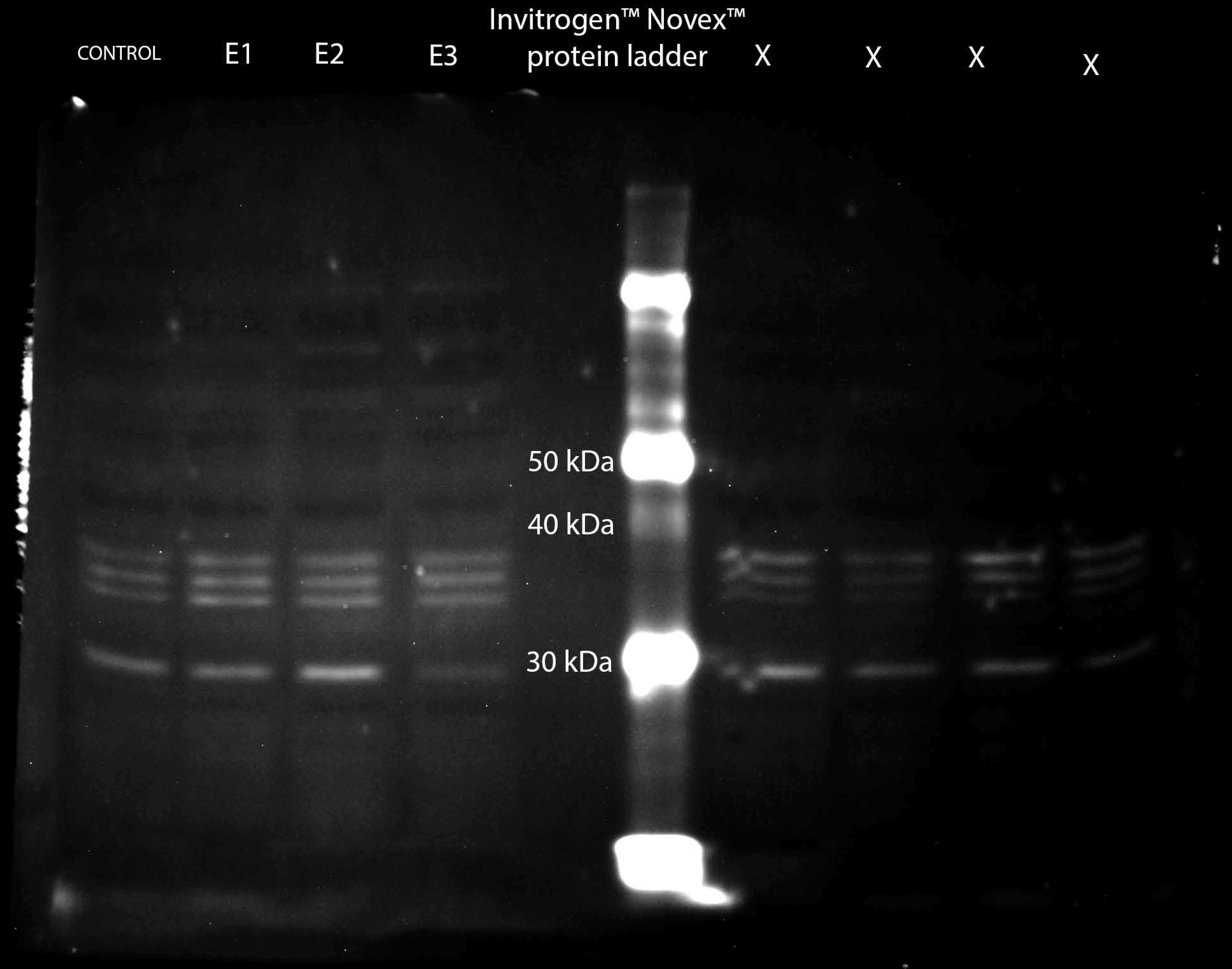
CONTROL
E1
E2
E3
Invitrogen™ Novex™ protein ladder
X
X
X
X
50 kDa
40 kDa
30 kDa

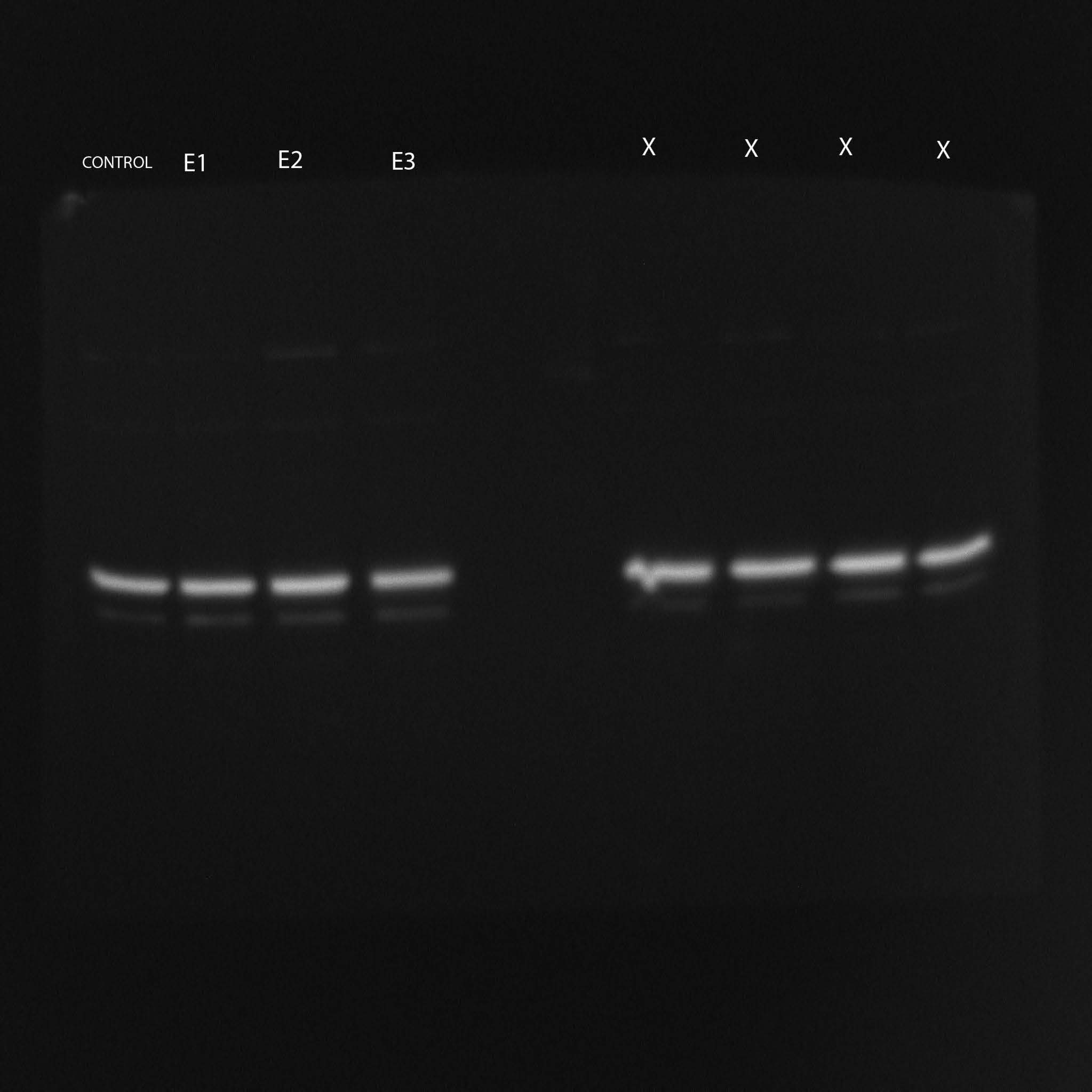
CONTROL
E1
E2
E3
X
X
X
X

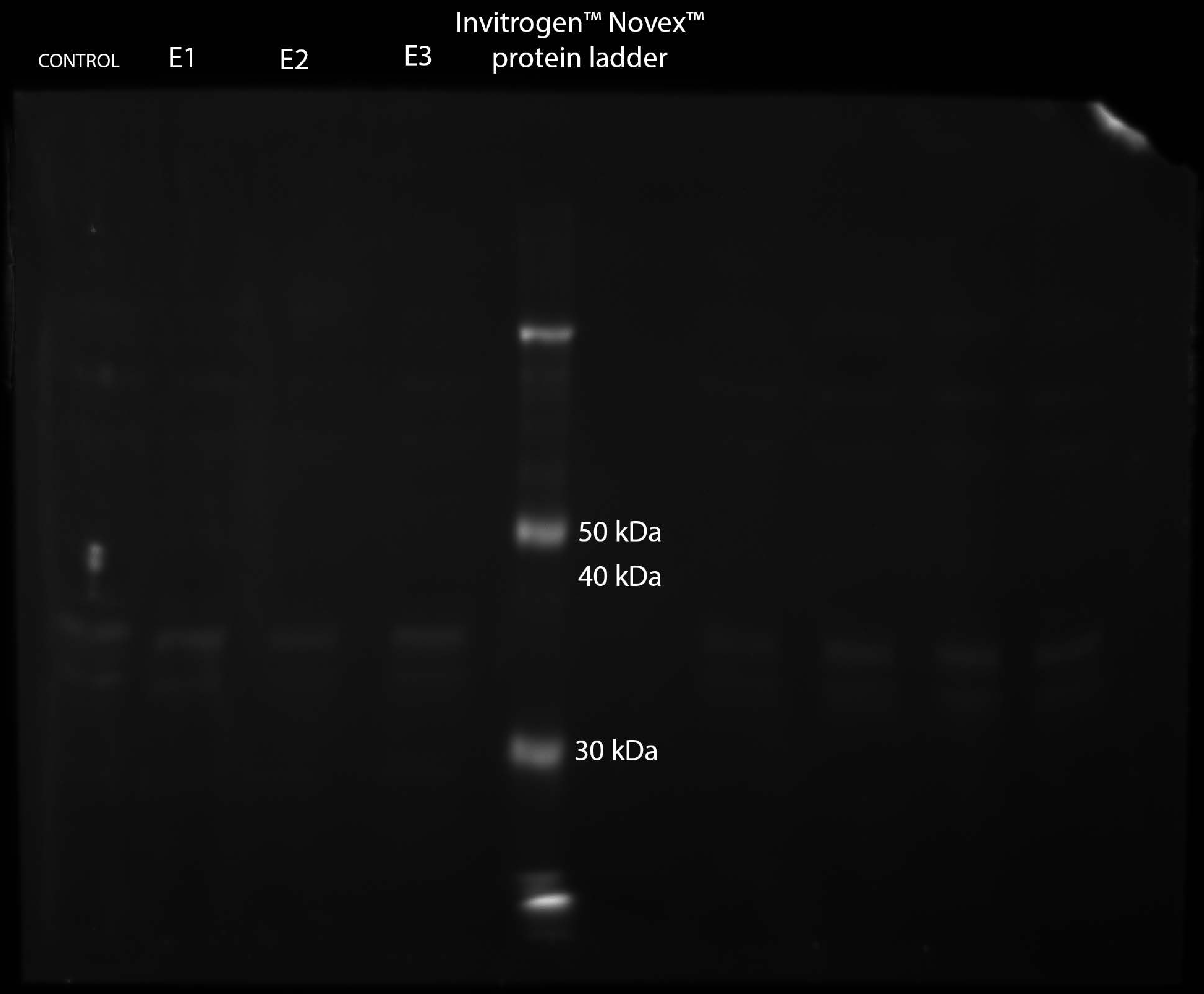
CONTROL
E1
E2
E3
Invitrogen™ Novex™ protein ladder
50 kDa
40 kDa
30 kDa

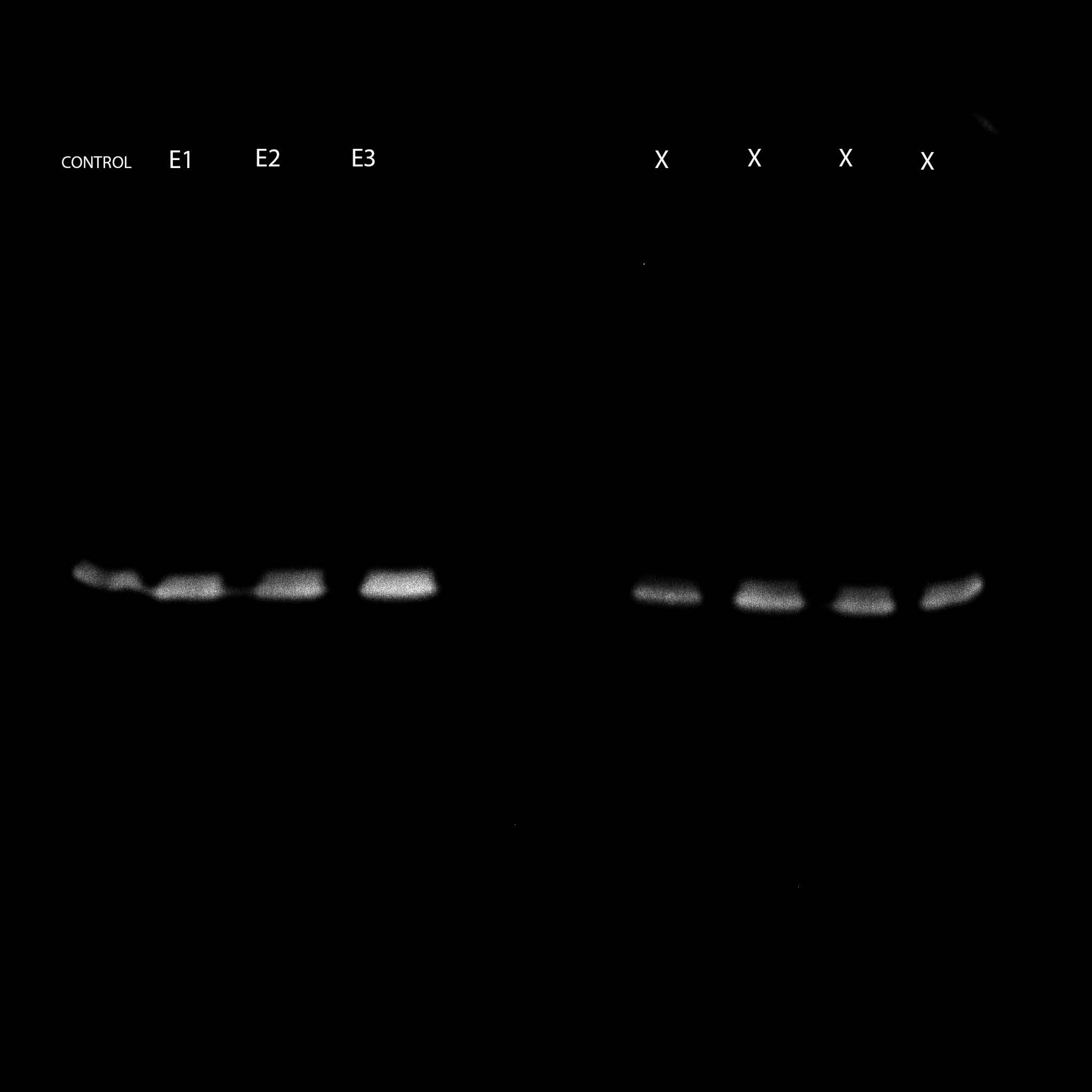
CONTROL
E1
E2
E3
X
X
X
X

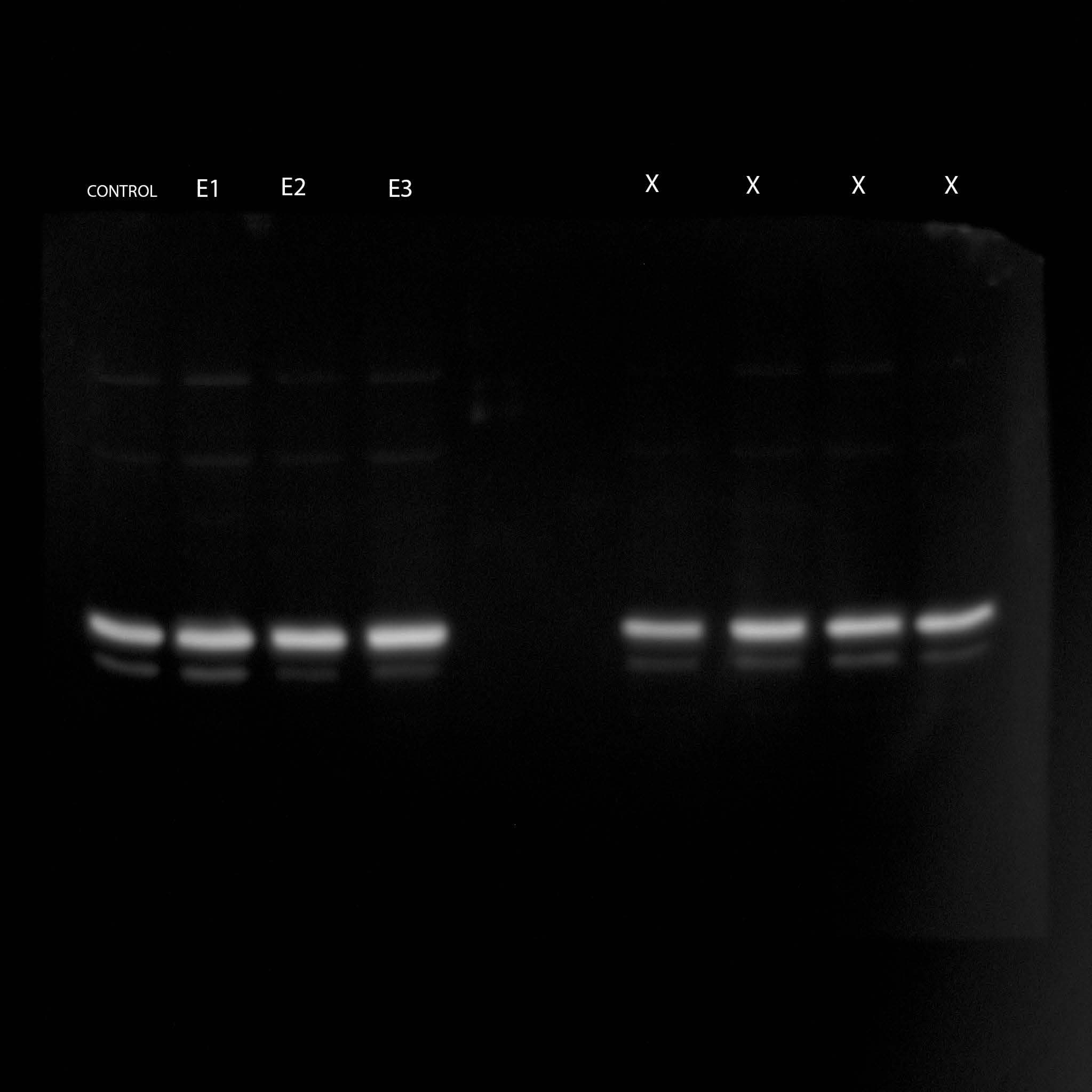
CONTROL
E1
E2
E3
X
X
X
X

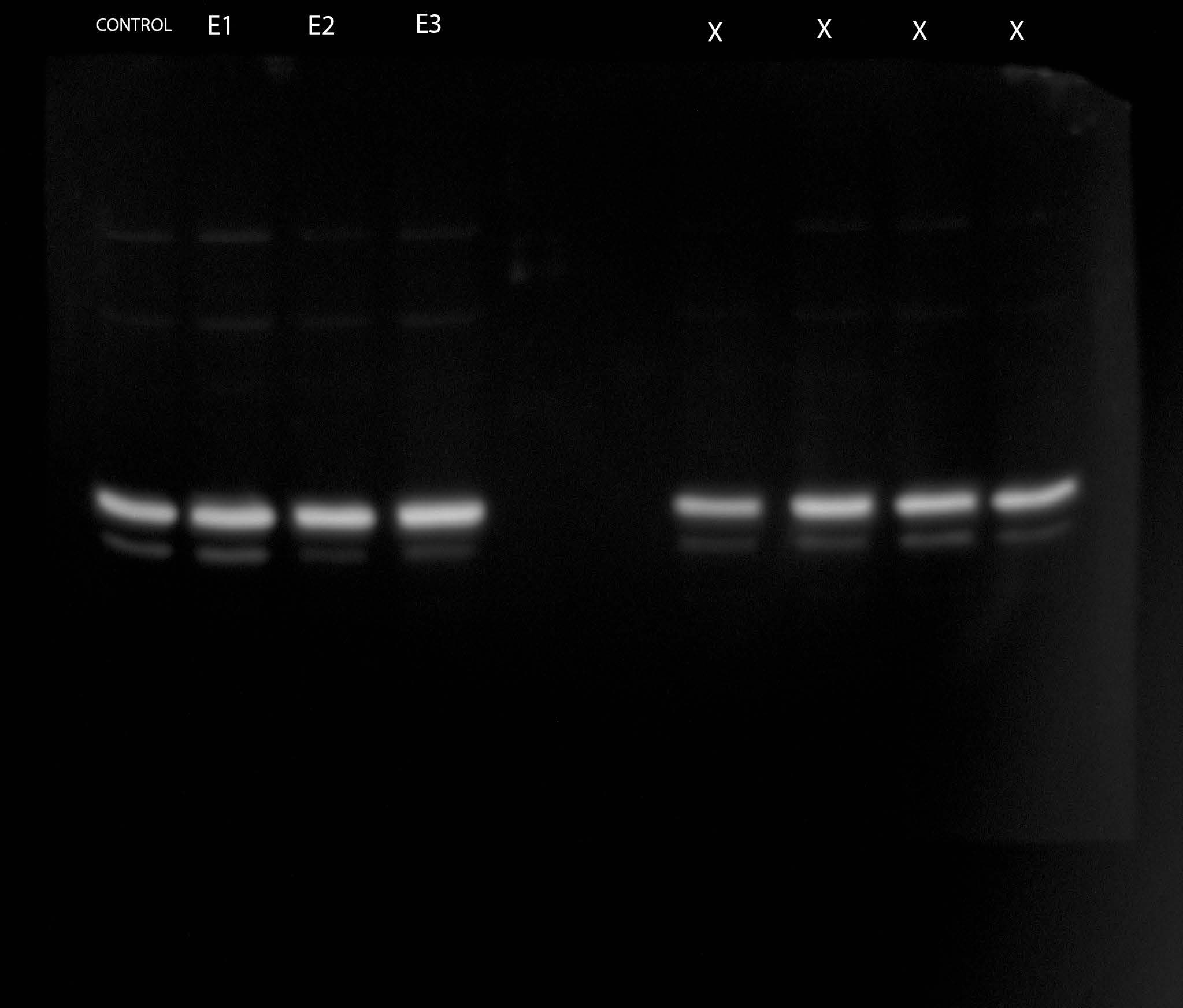
CONTROL
E1
E2
E3
X
X
X
X

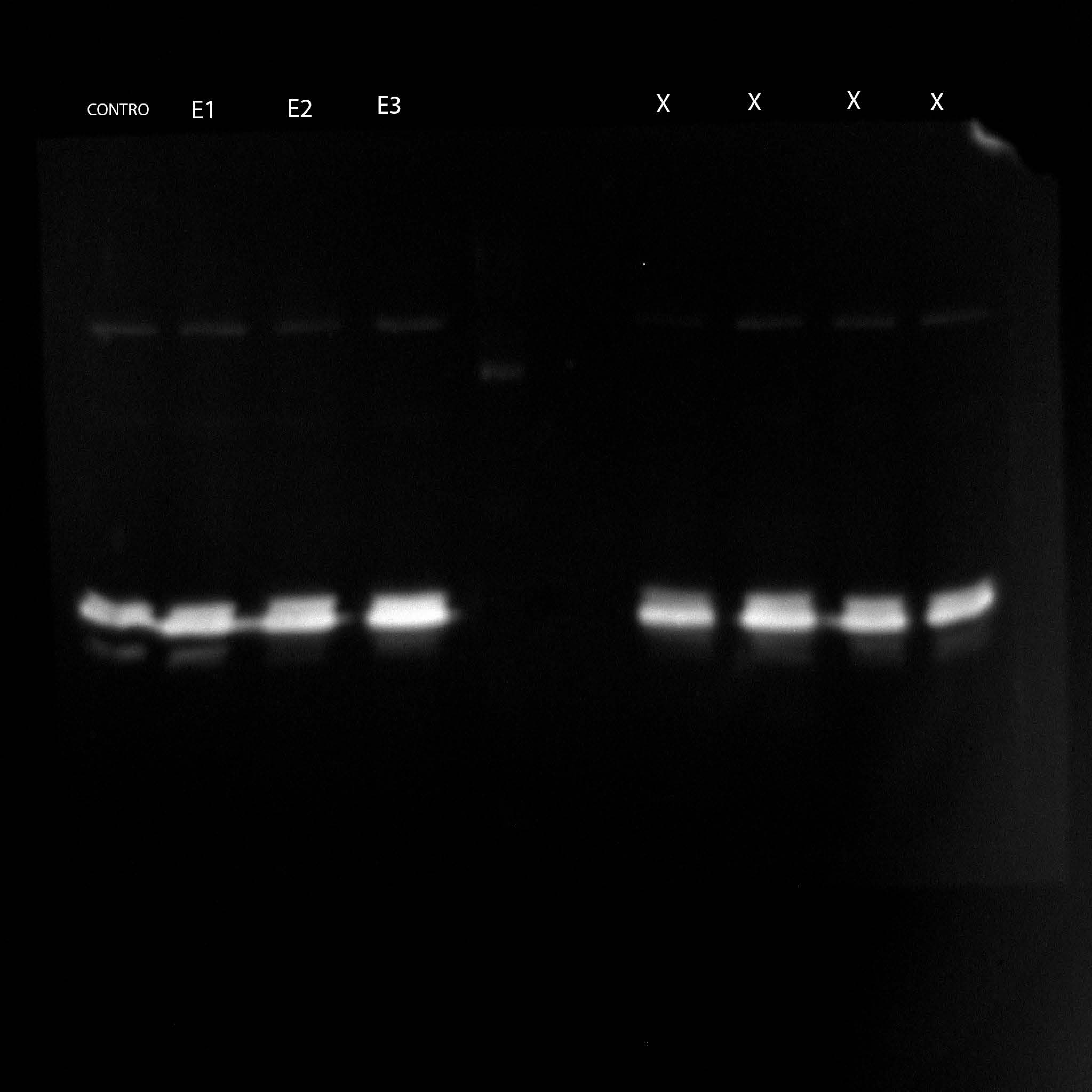
CONTRO
E1
E2
E3
X
X
X
X

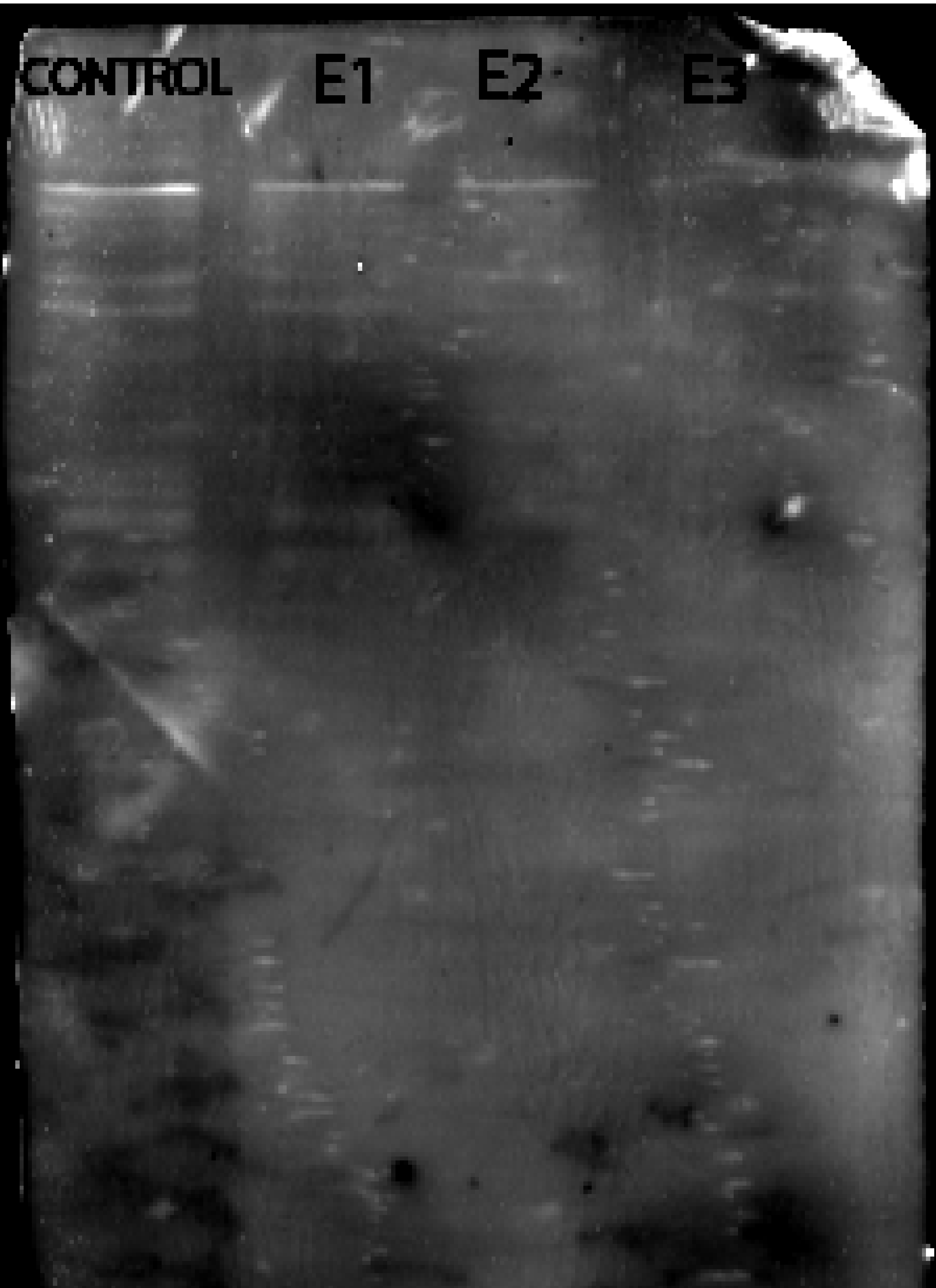
CONTROL
E1
E2
E3

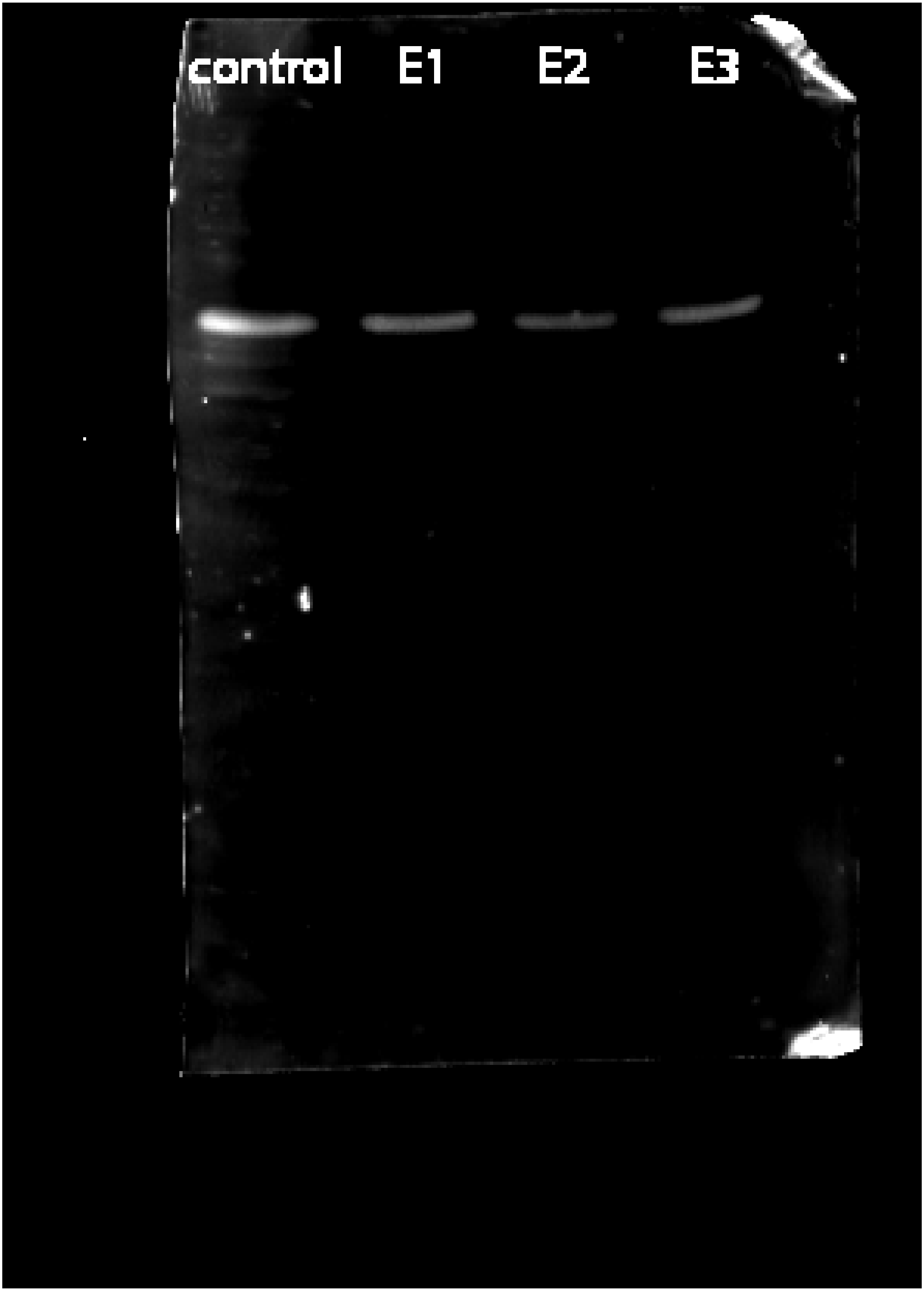
control
E1
E2
E3

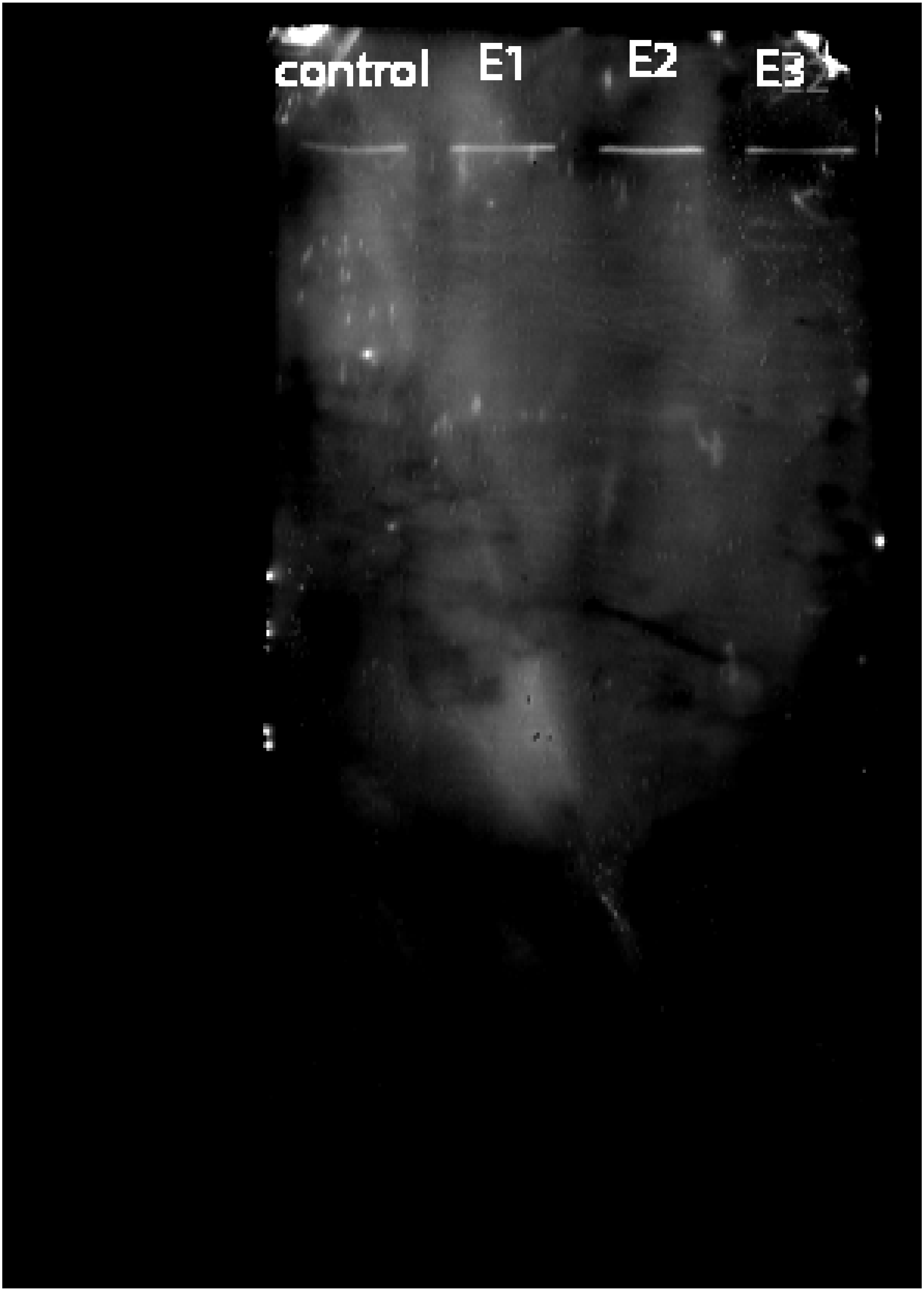
control
E1
E2
E3

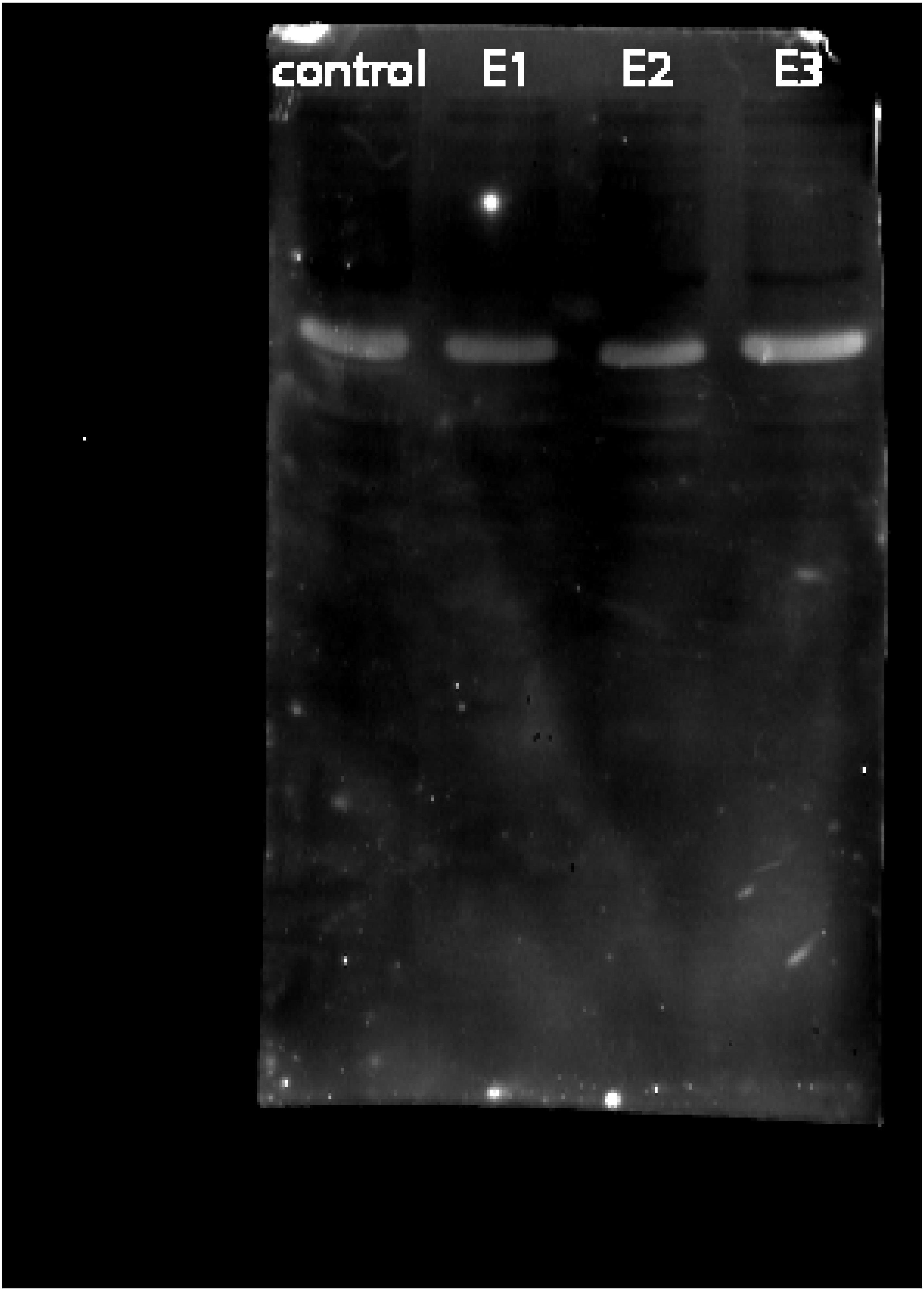
control
E1
E2
E3

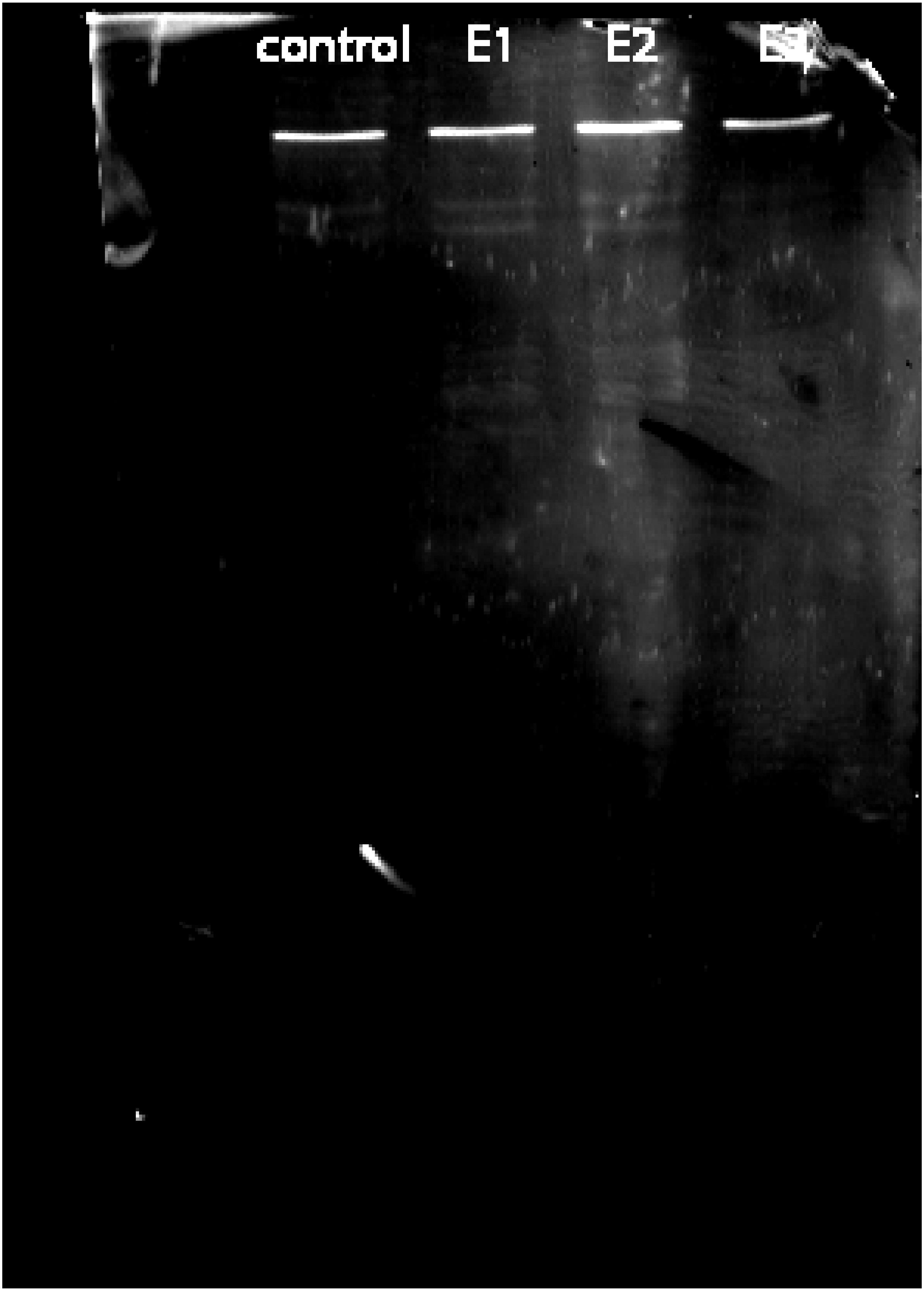

control
E1
E2

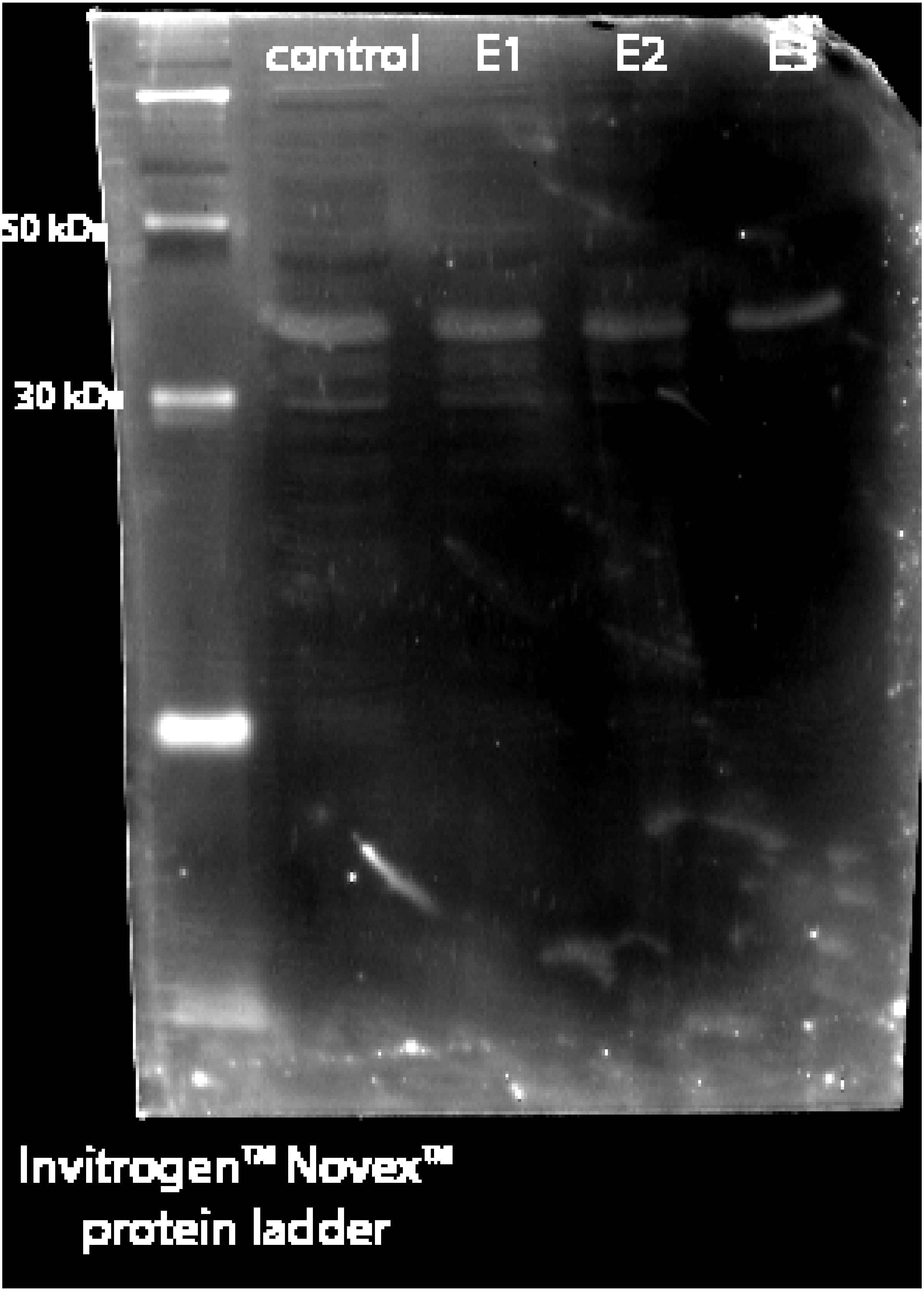
control
E1
E2
E3
50 kD
30 kD
Invitrogen™ Novex™
protein ladder

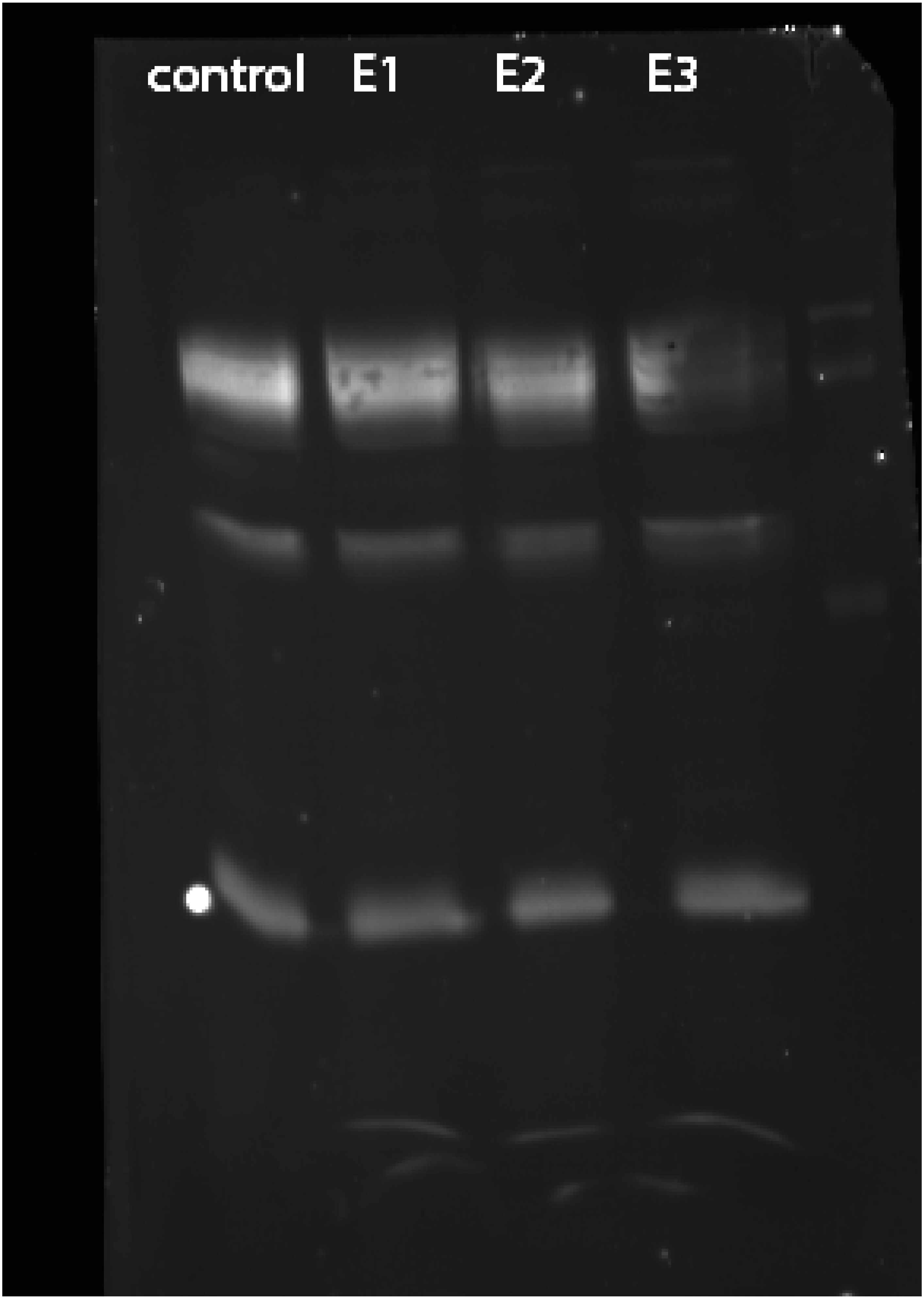
control
E1
E2
E3

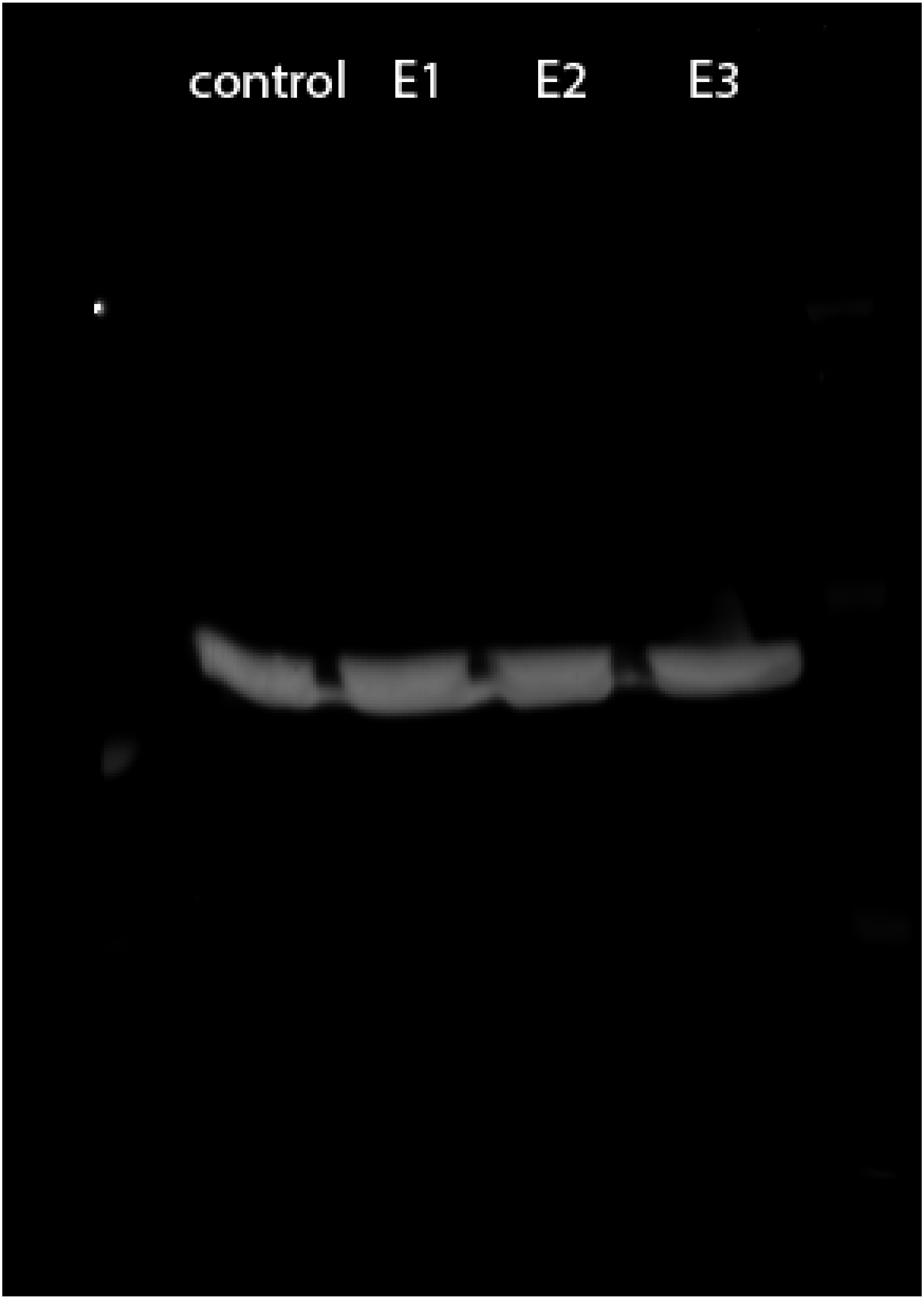
control E1 E2 E3

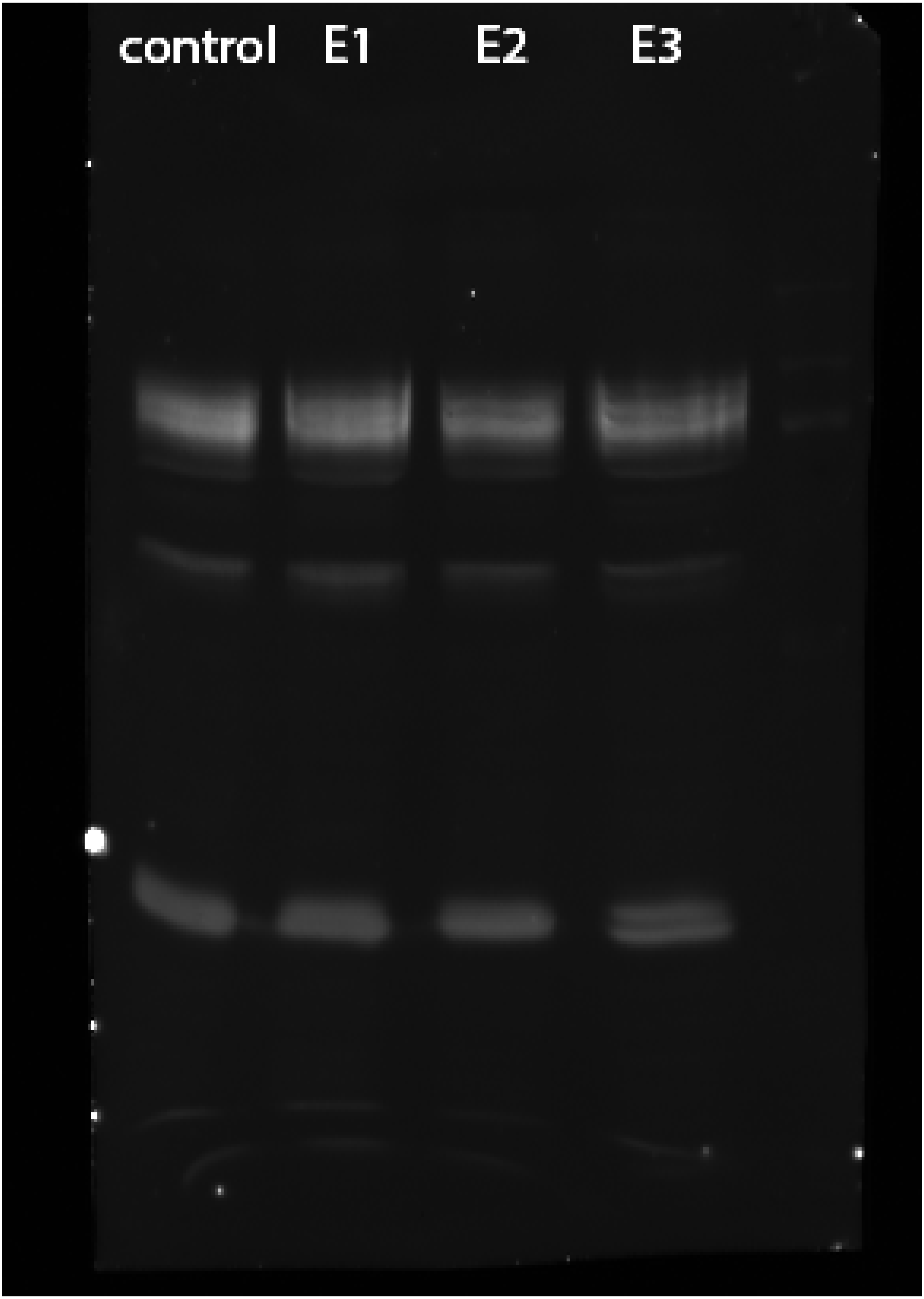
control
E1
E2
E3

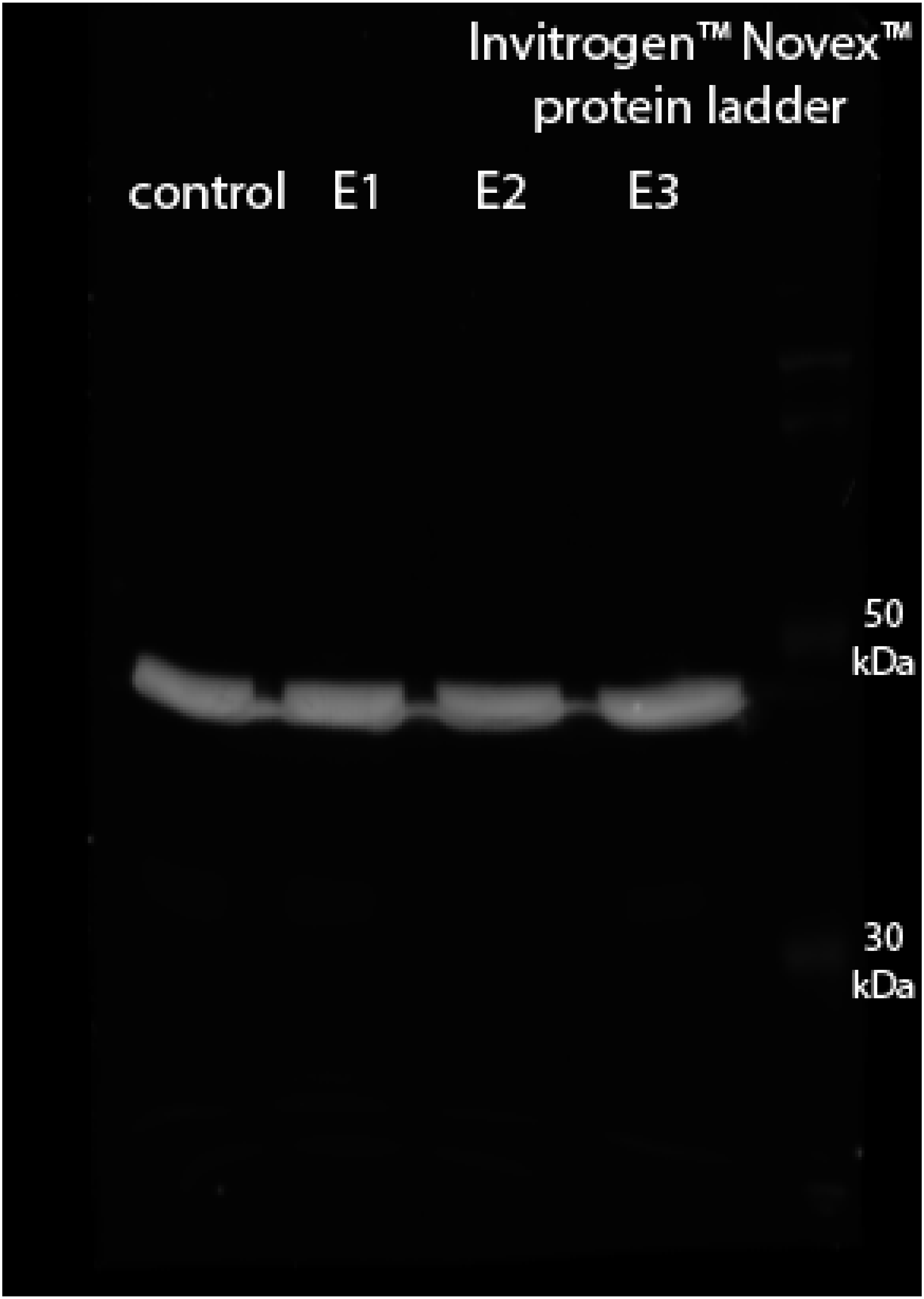
Invitrogen™ Novex™
protein ladder
control
E1
E2
E3
50
kDa
30
kDa

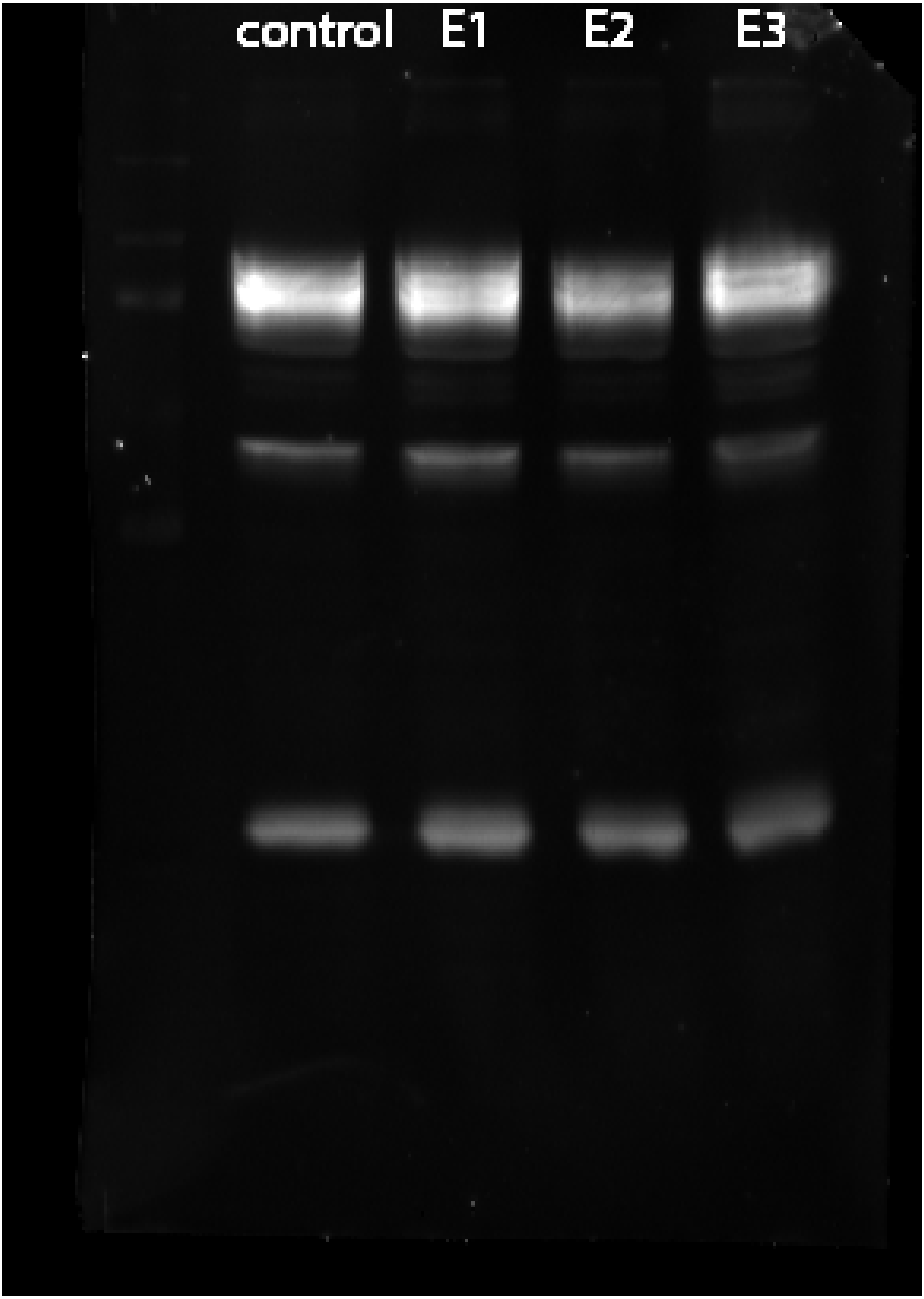
control
E1
E2
E3

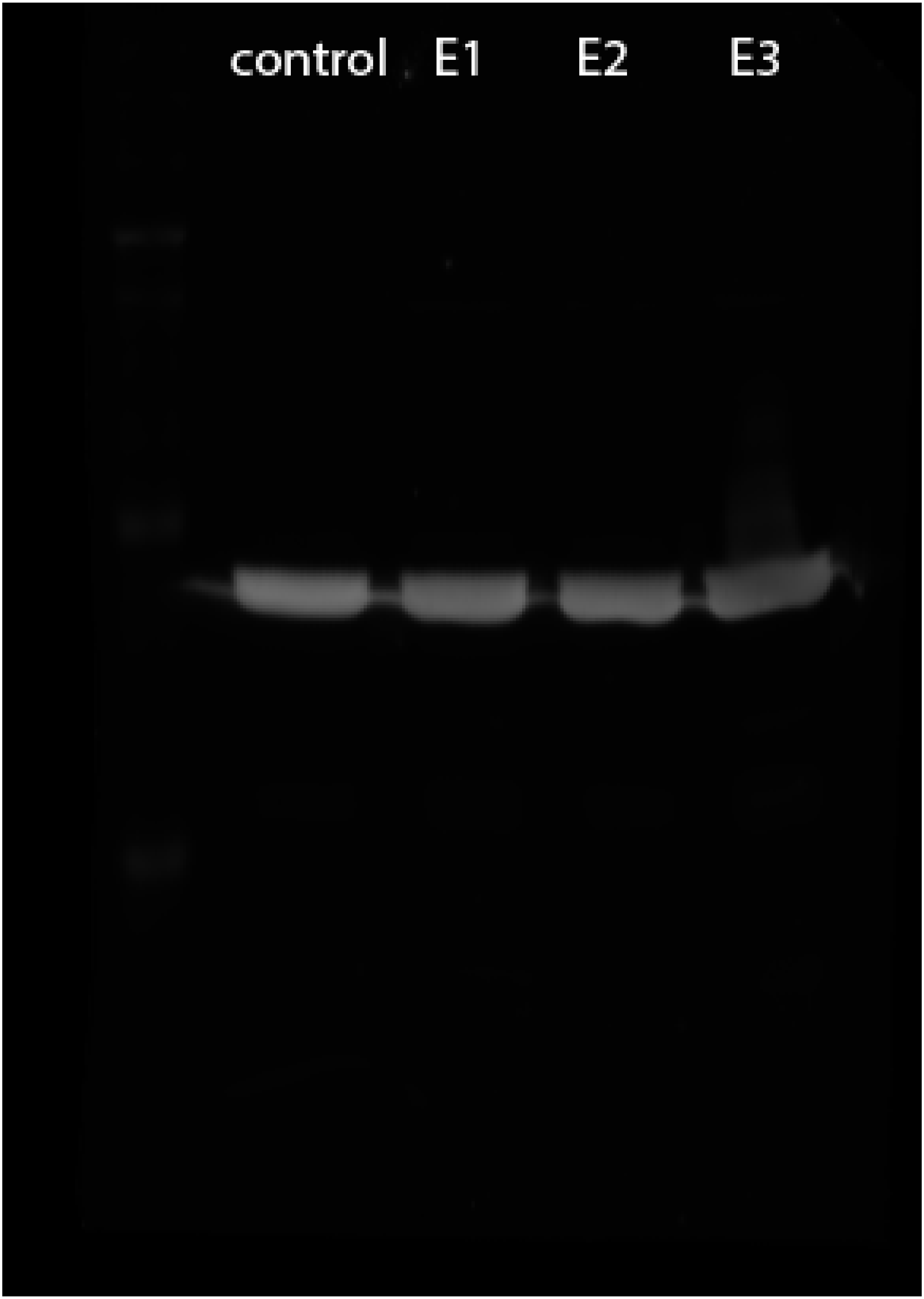

control
E1
E2
E3

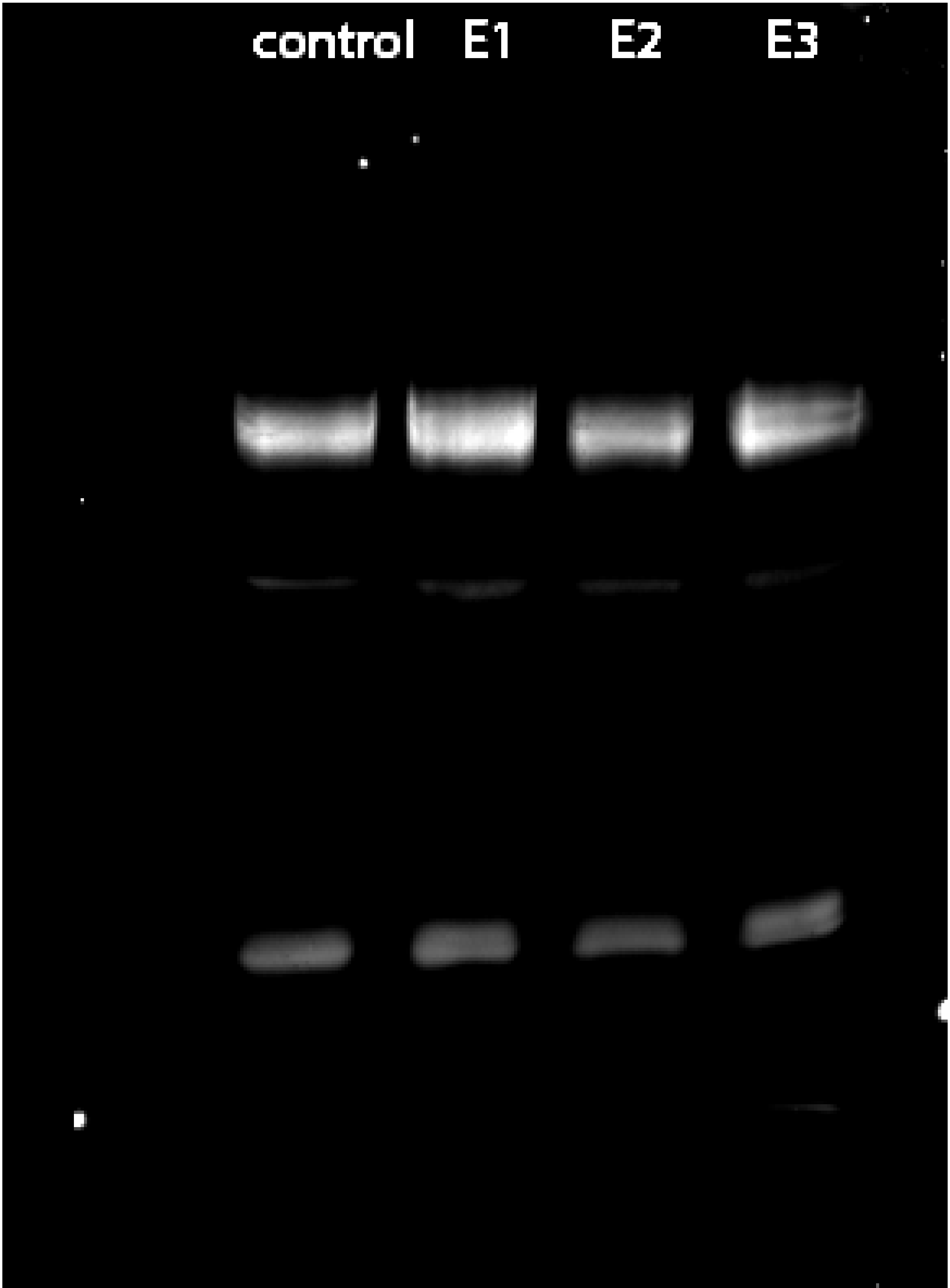
control
E1
E2
E3

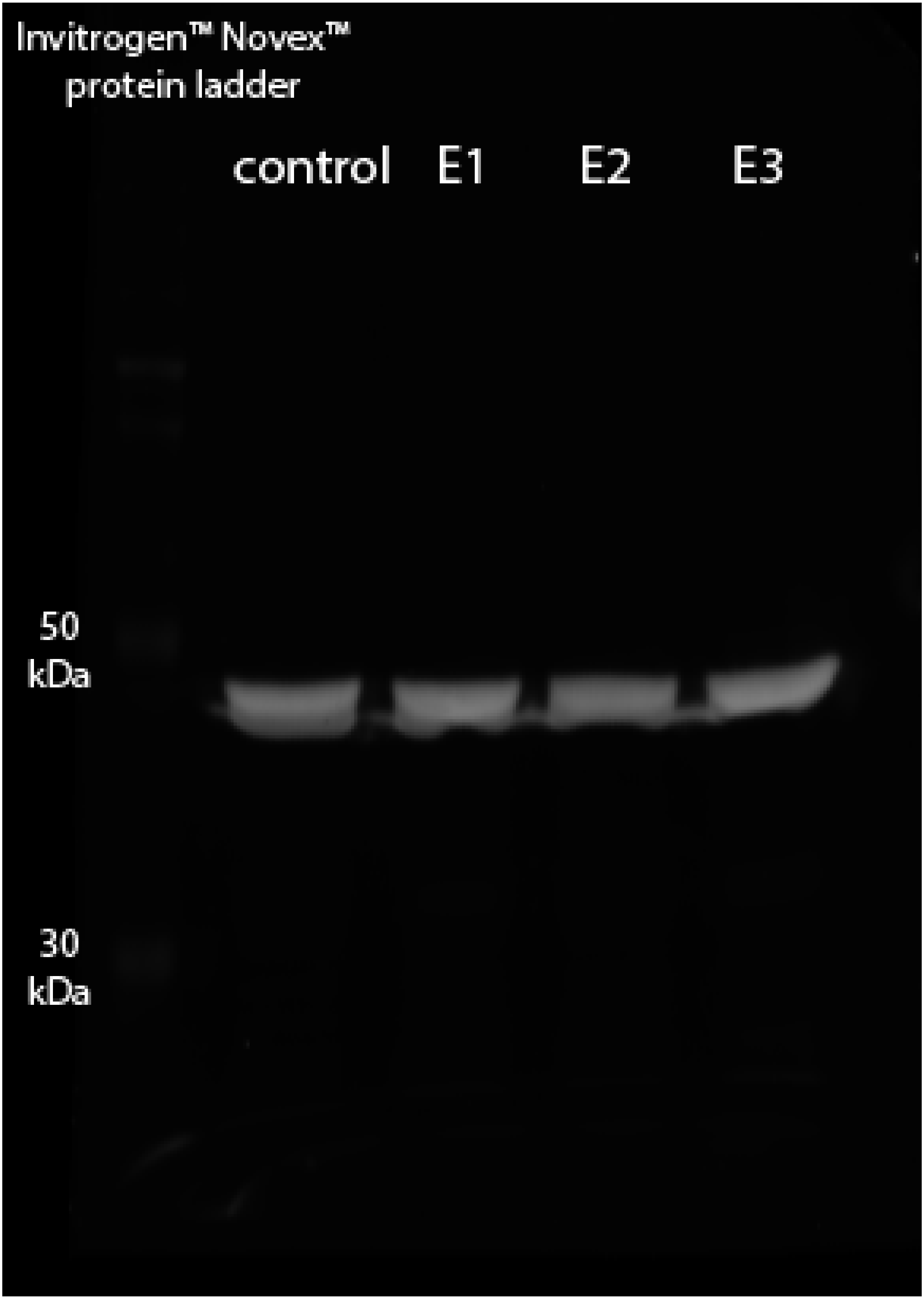
Invitrogen™ Novex™
protein ladder
control
E1
E2
E3
50
kDa
30
kDa

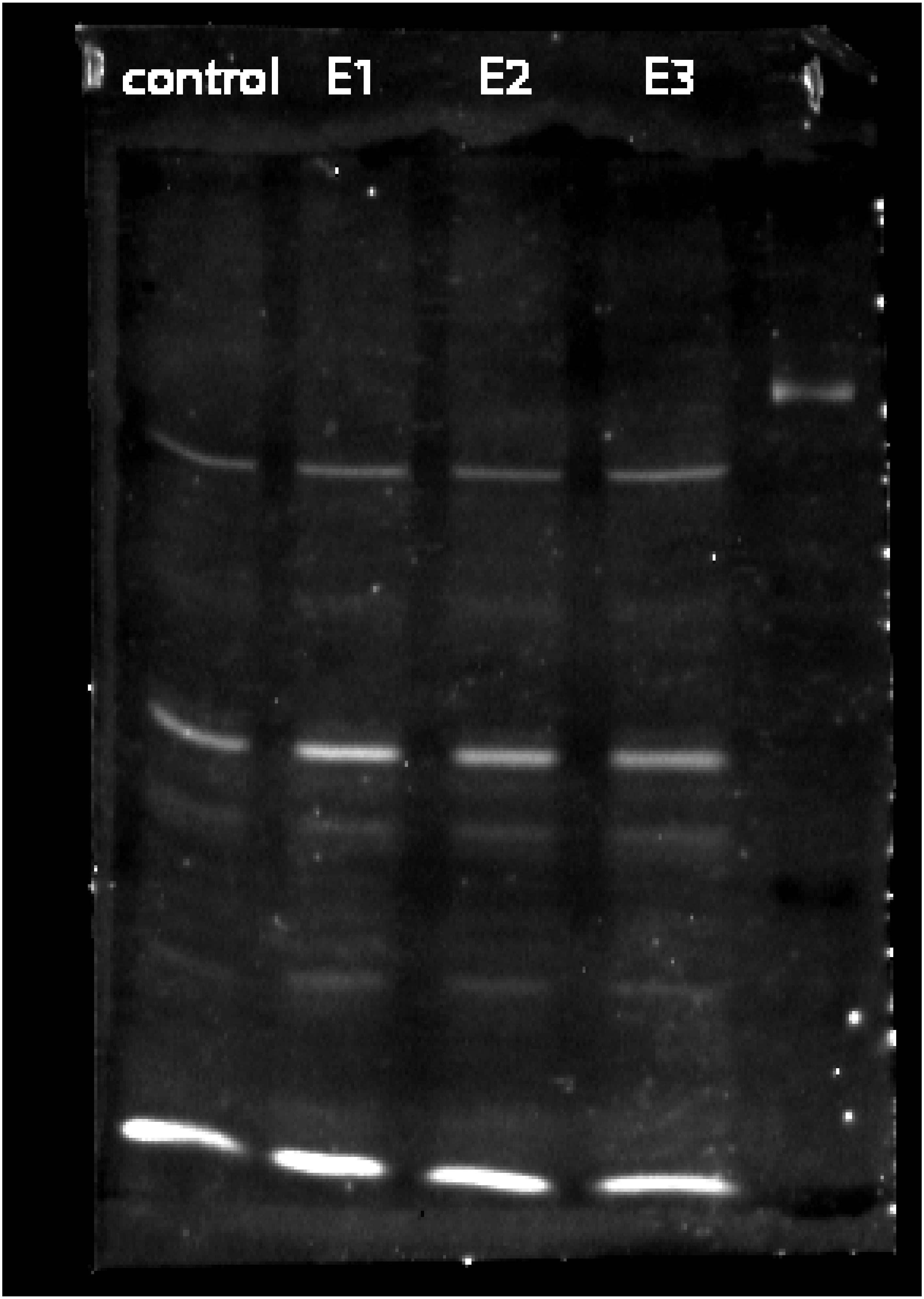
control
E1
E2
E3

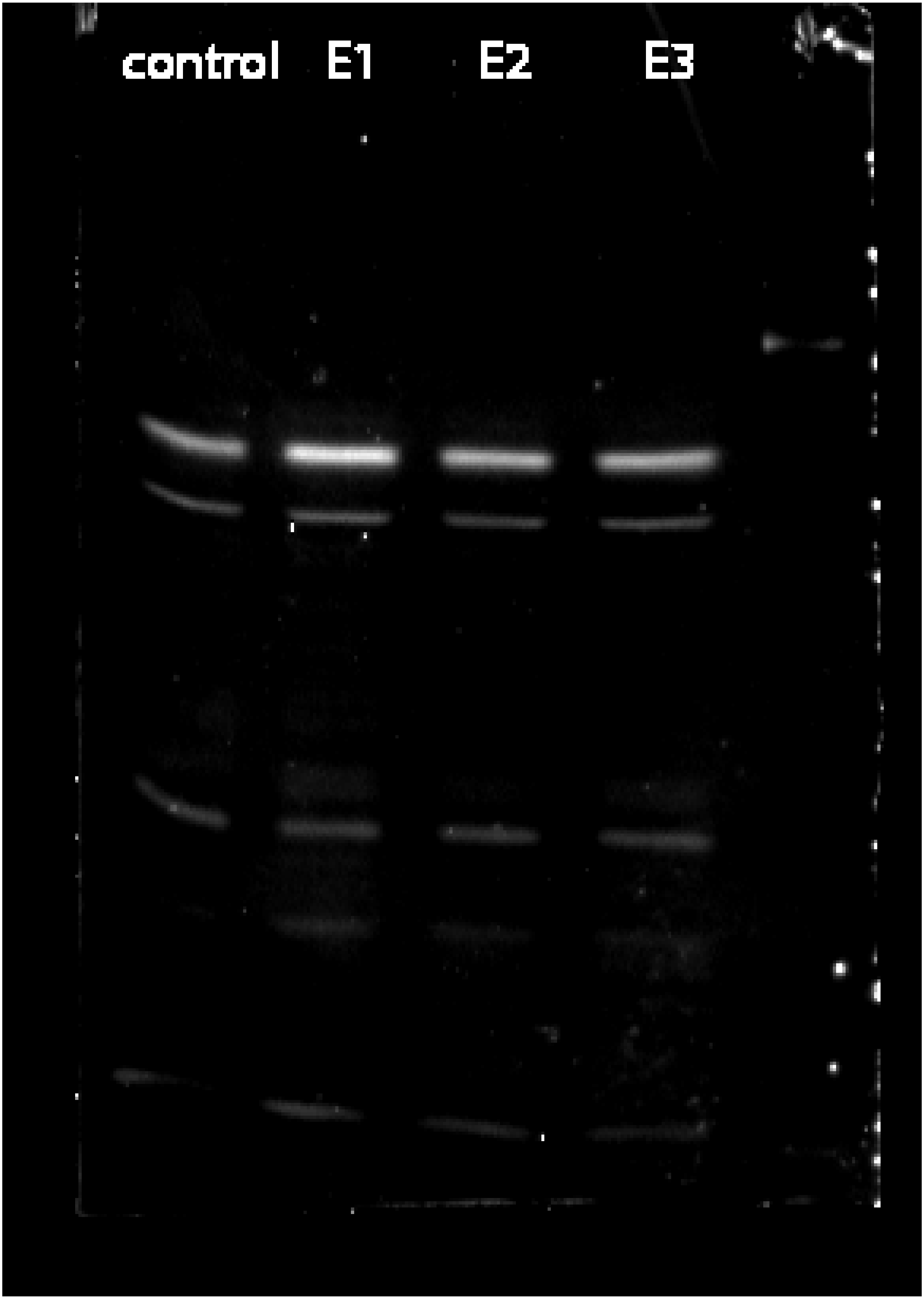
control
E1
E2
E3

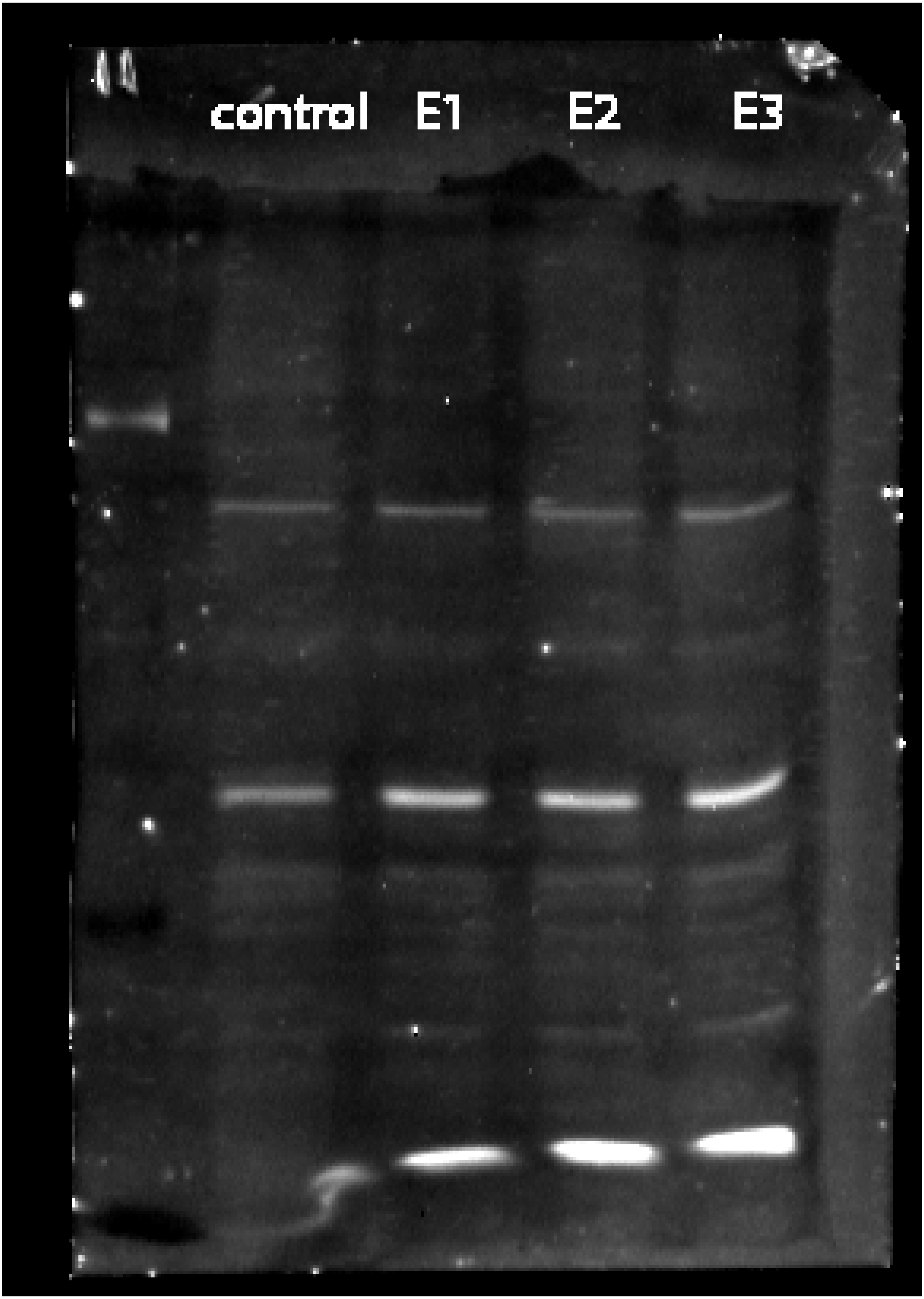
control
E1
E2
E3

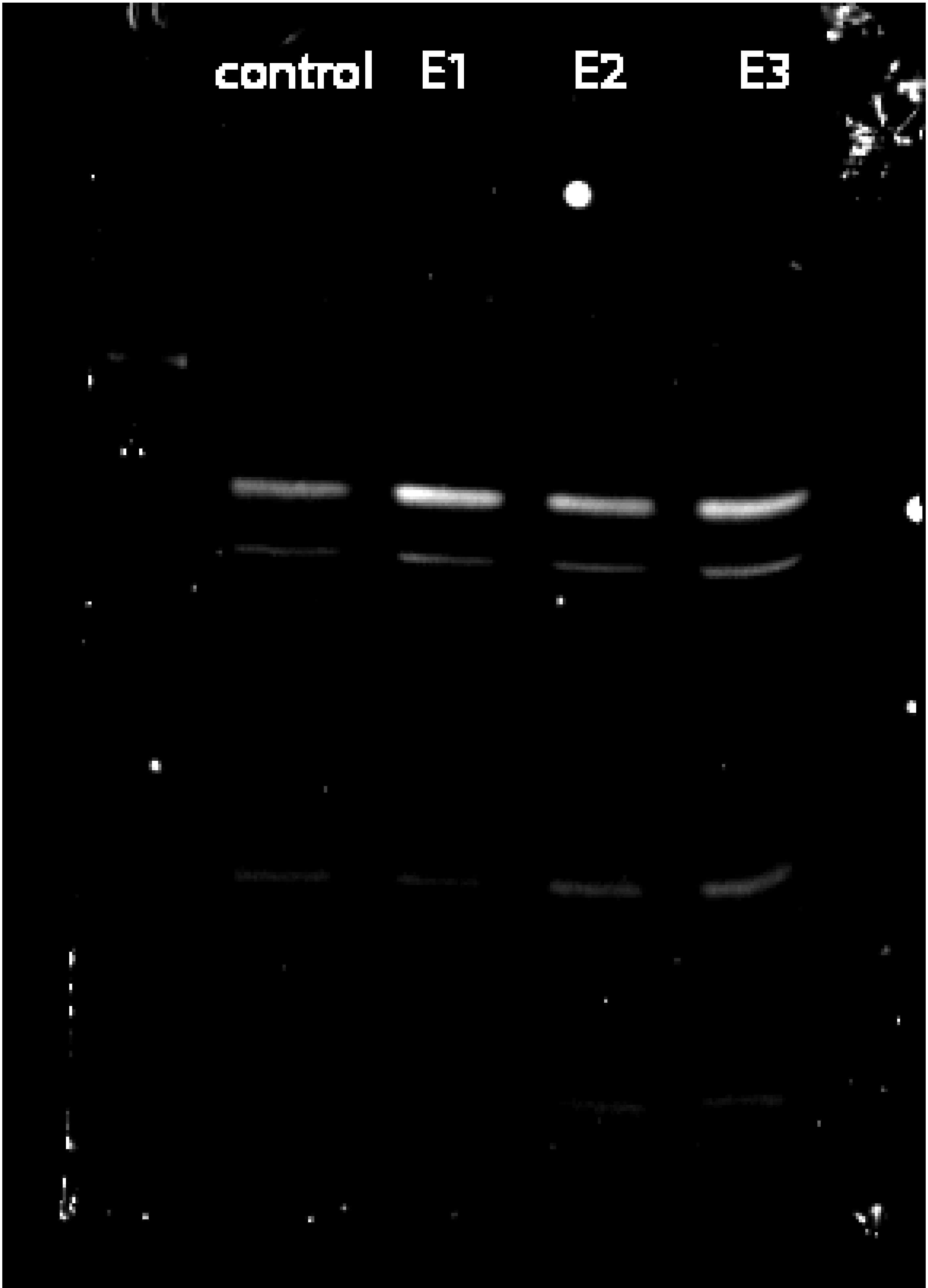
control
E1
E2
E3

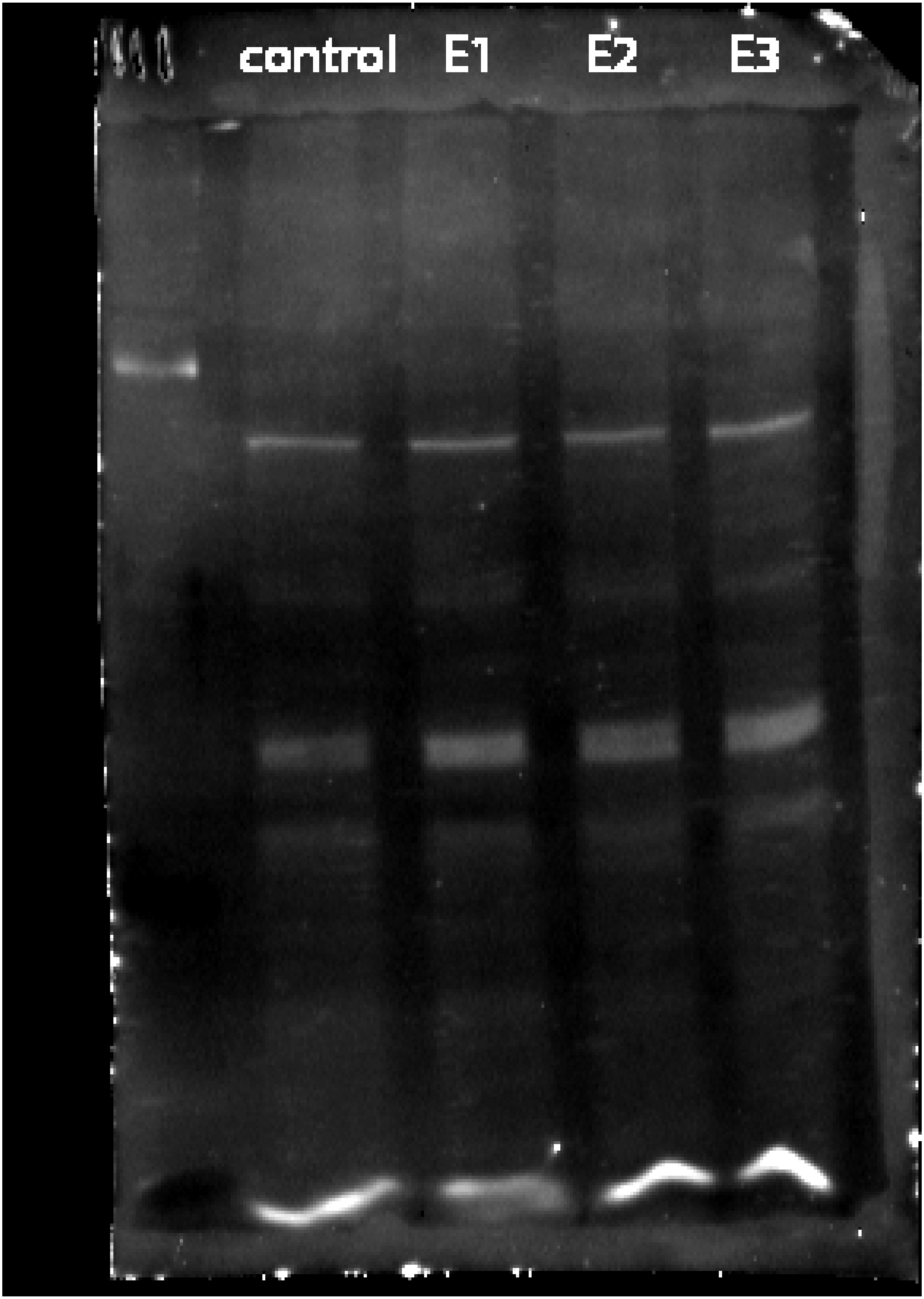
control
E1
E2
E3

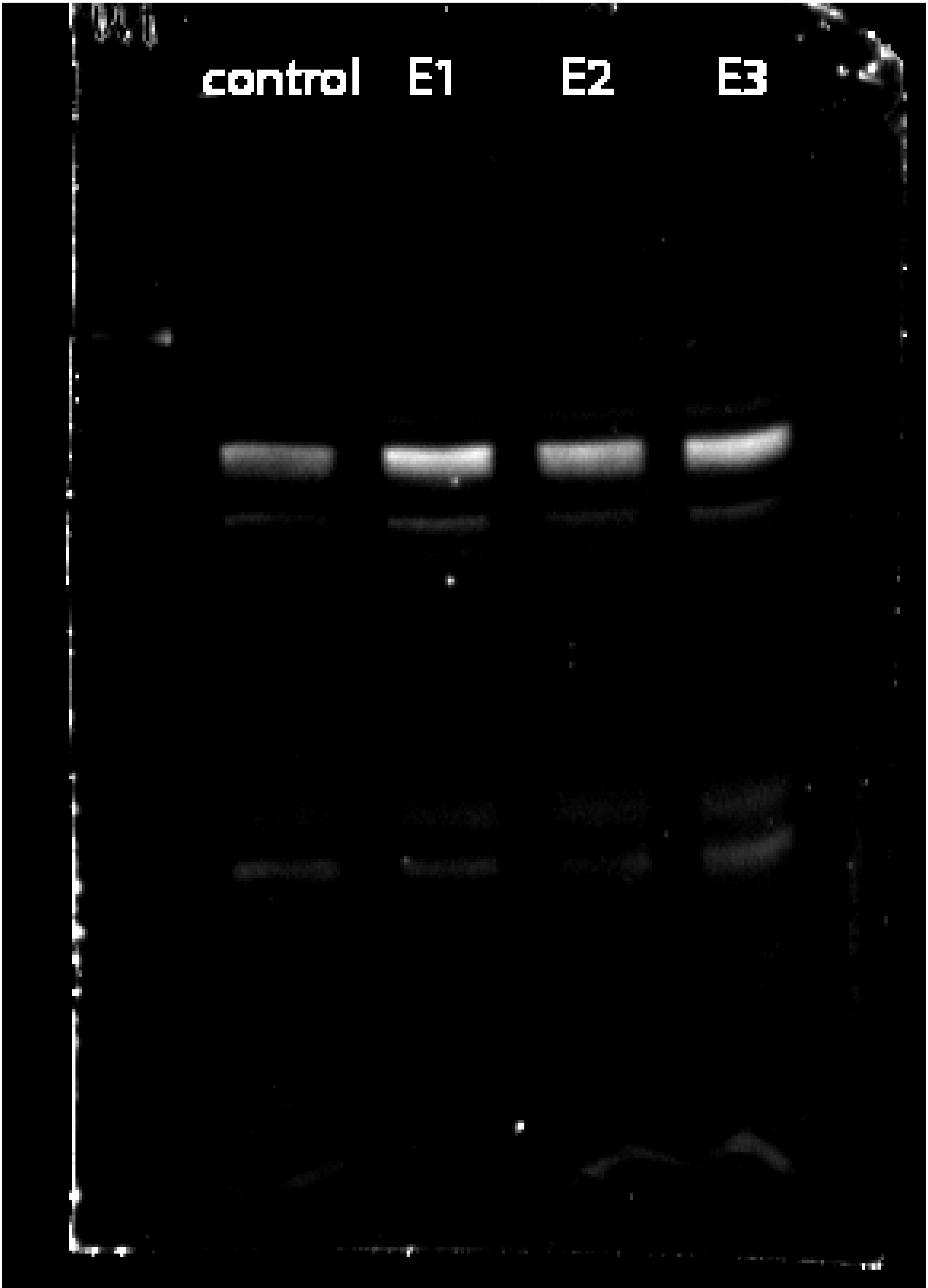
control
E1
E2
E3

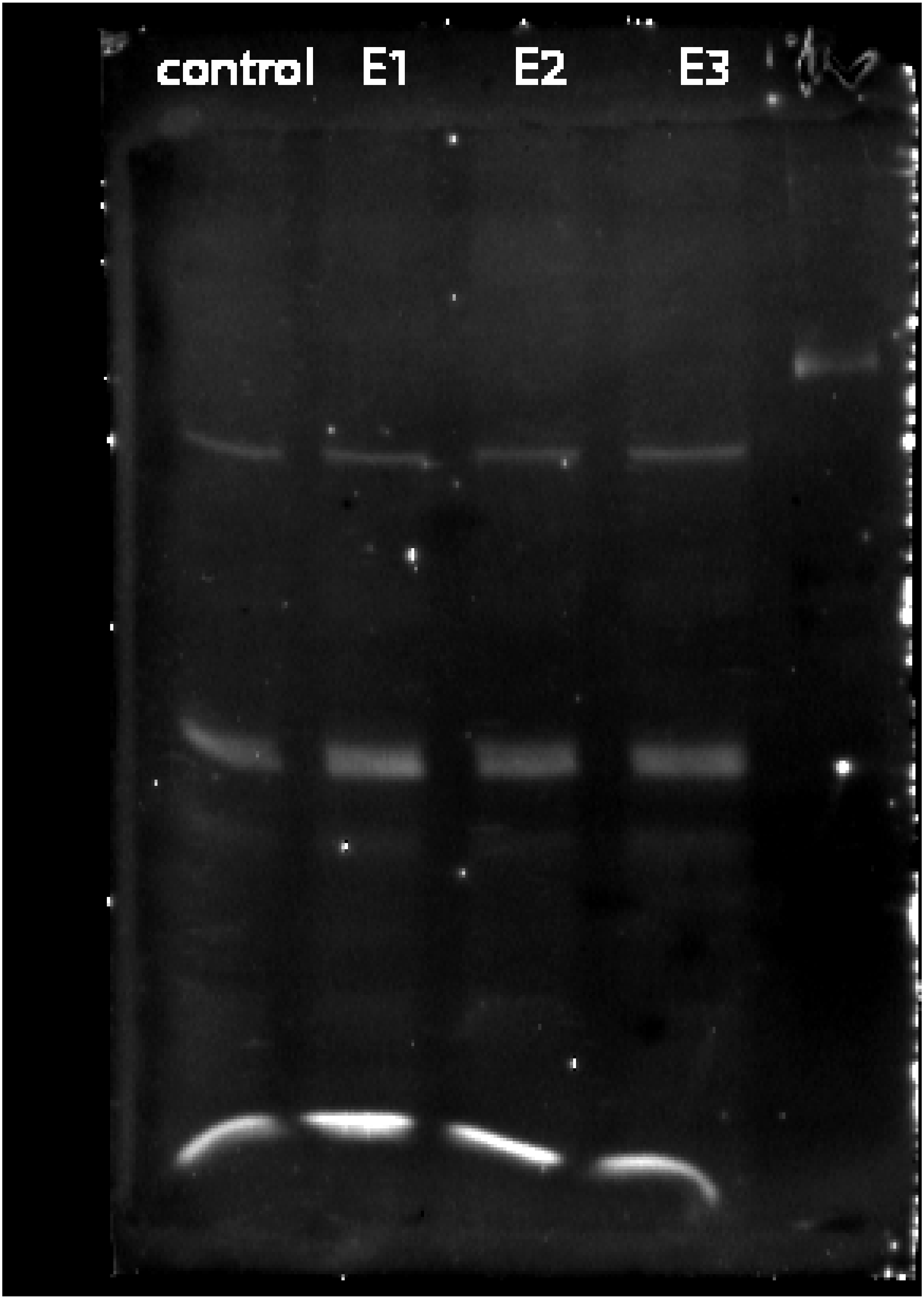
control
E1
E2
E3

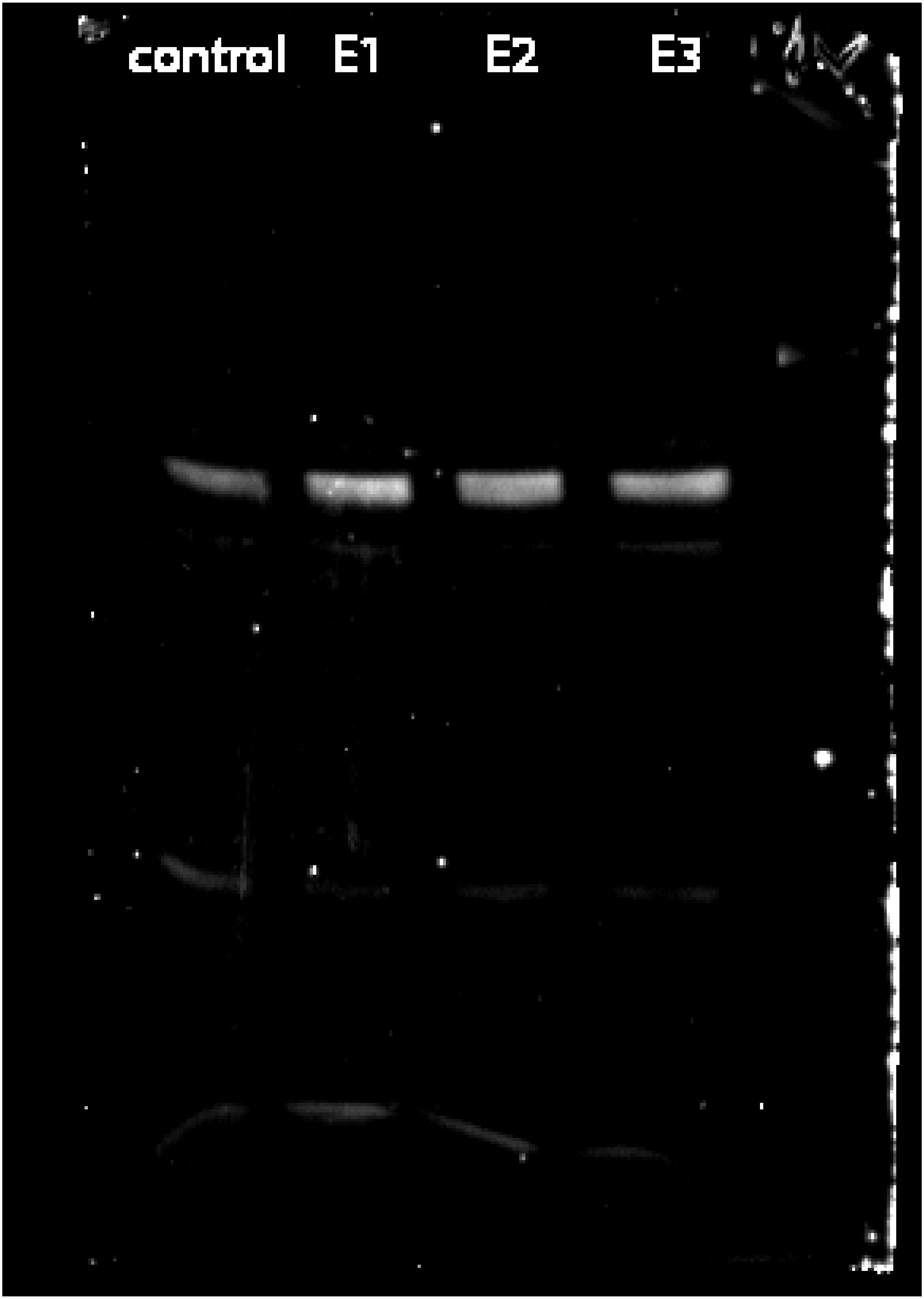
control
E1
E2
E3

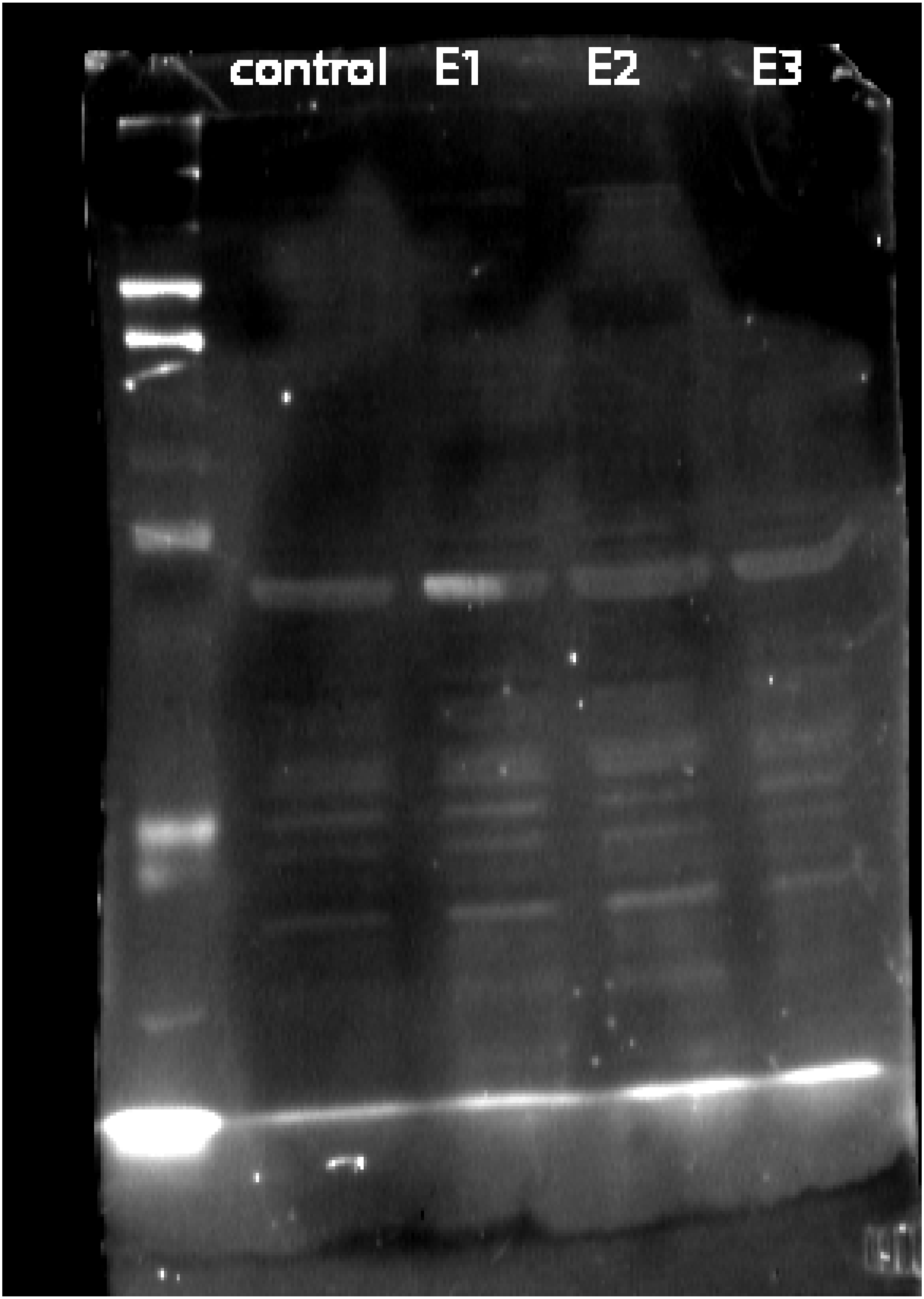
control
E1
E2
E3

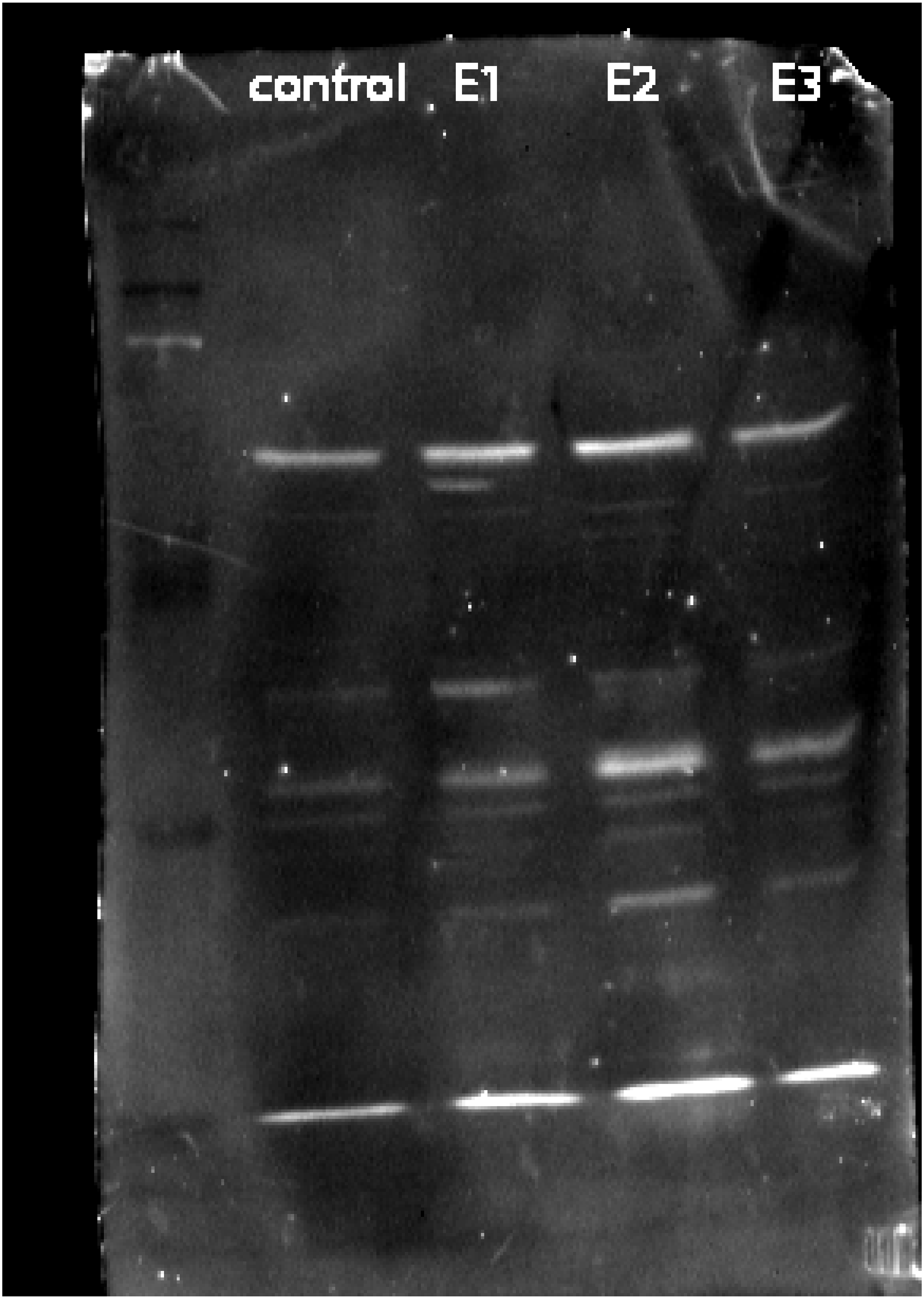
control
E1
E2
E3

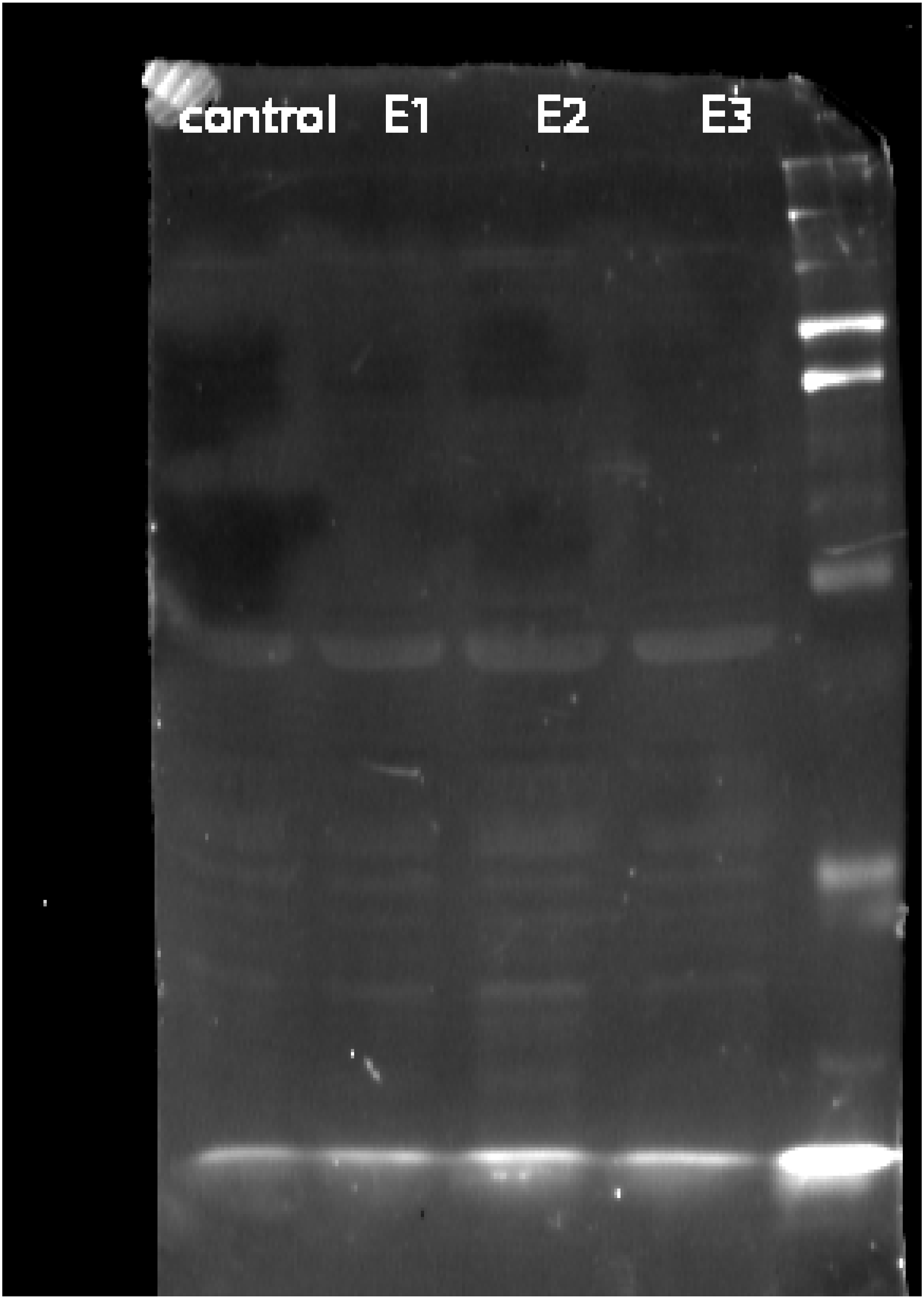
control
E1
E2
E3

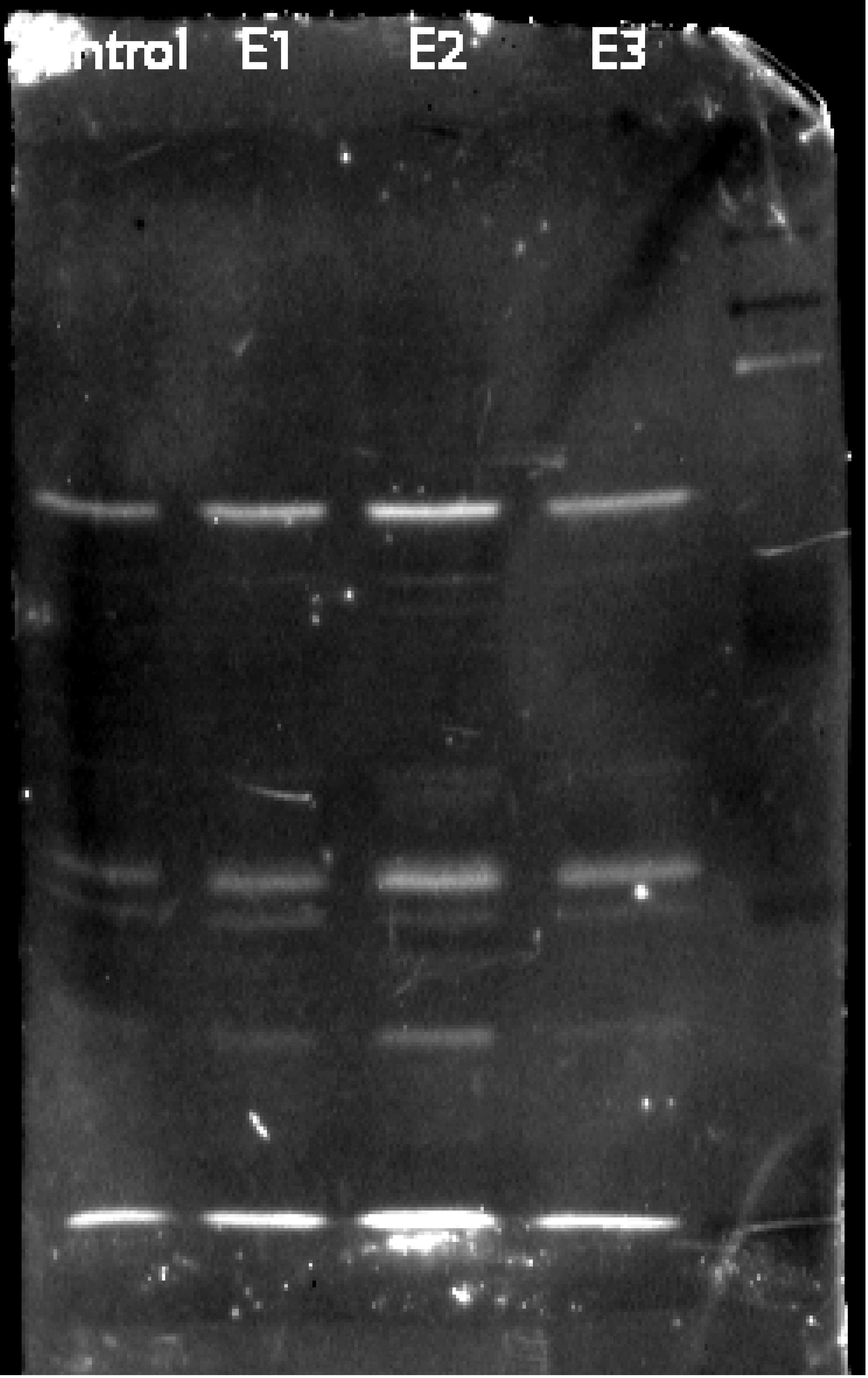
E1
E2
E3

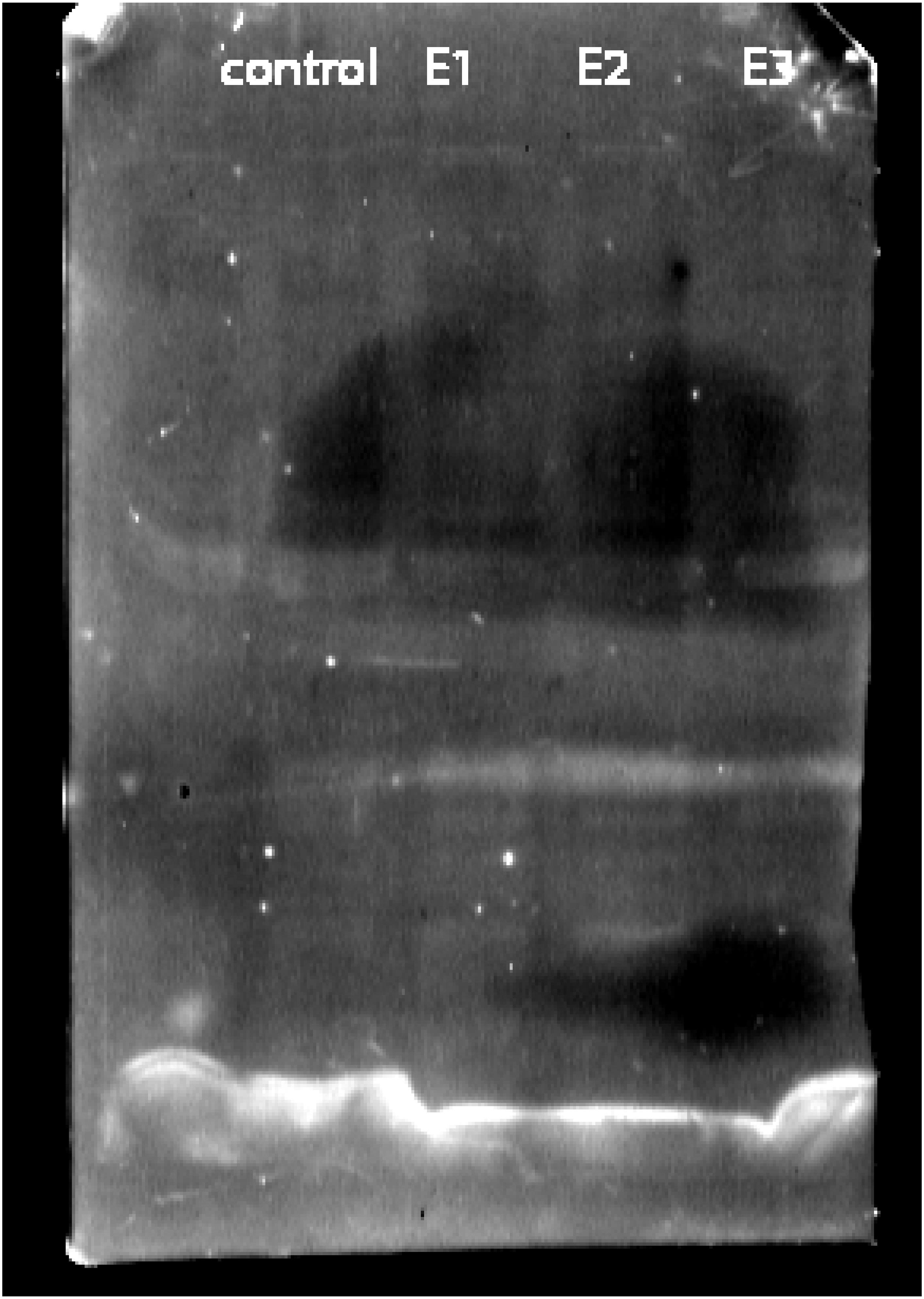
control
E1
E2
E3

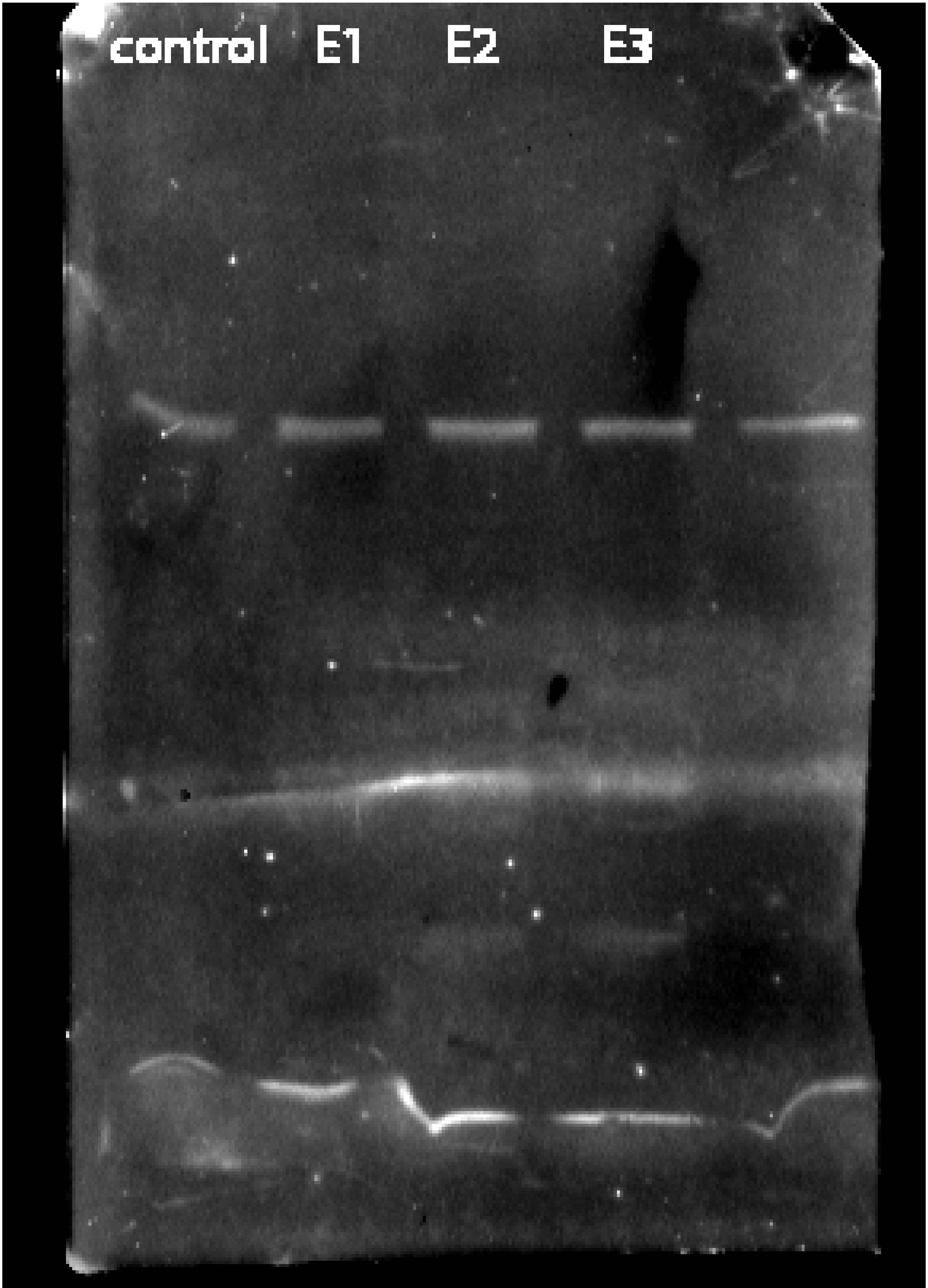
control
E1
E2
E3

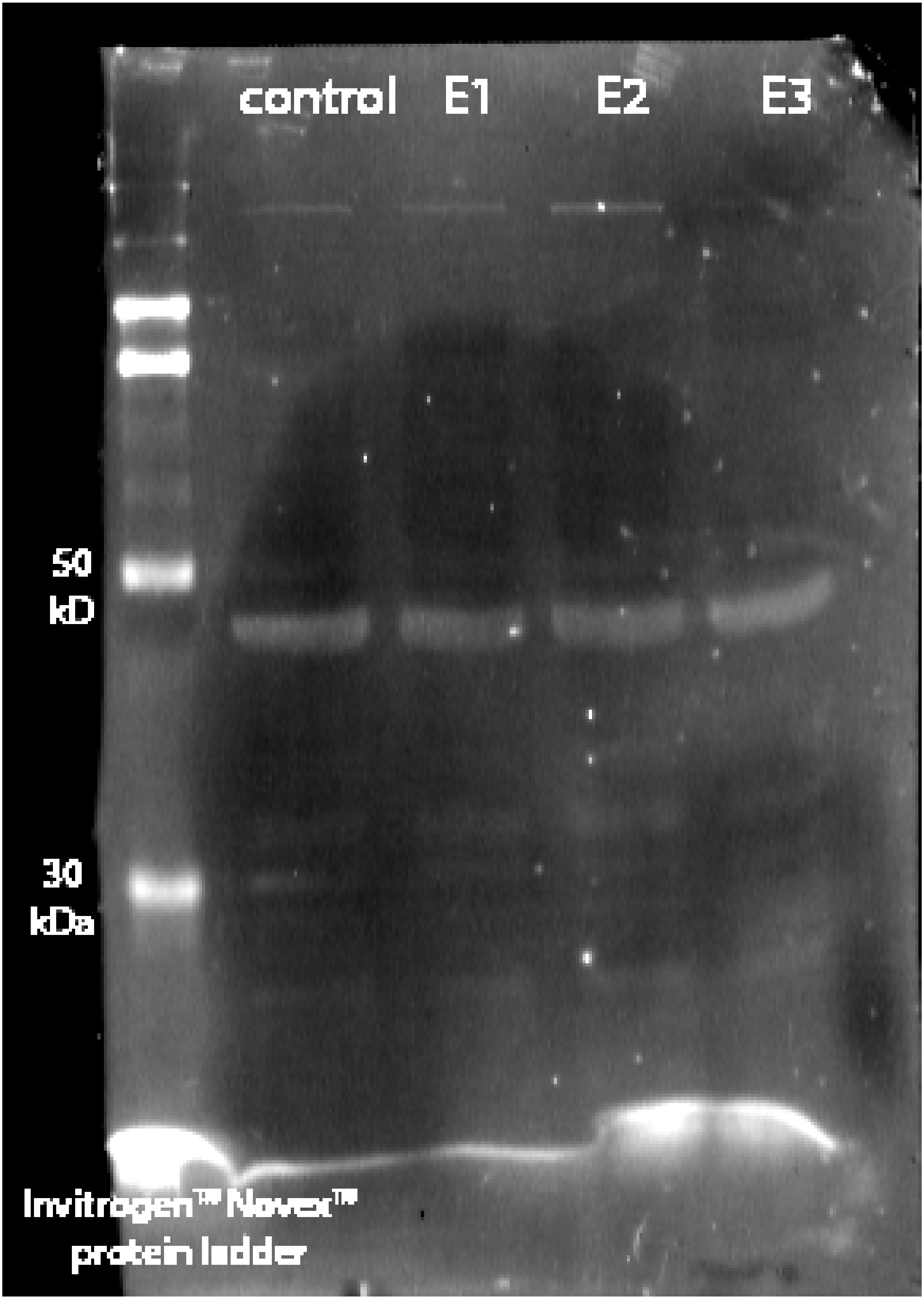
control
E1
E2
E3
50
kD
30
kDa
Invitrogen™ Novex™
protein ladder

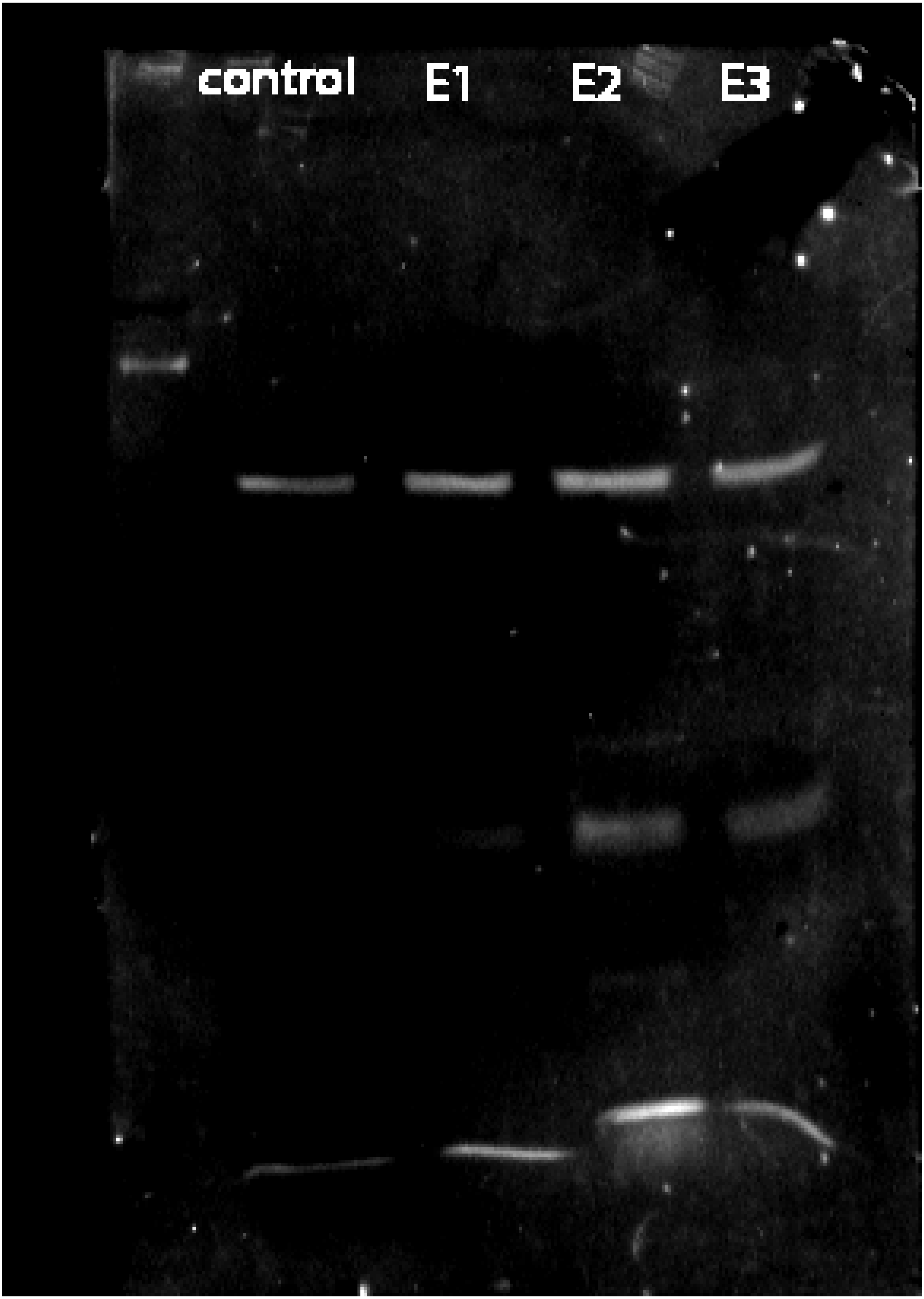
control
E1
E2
E3

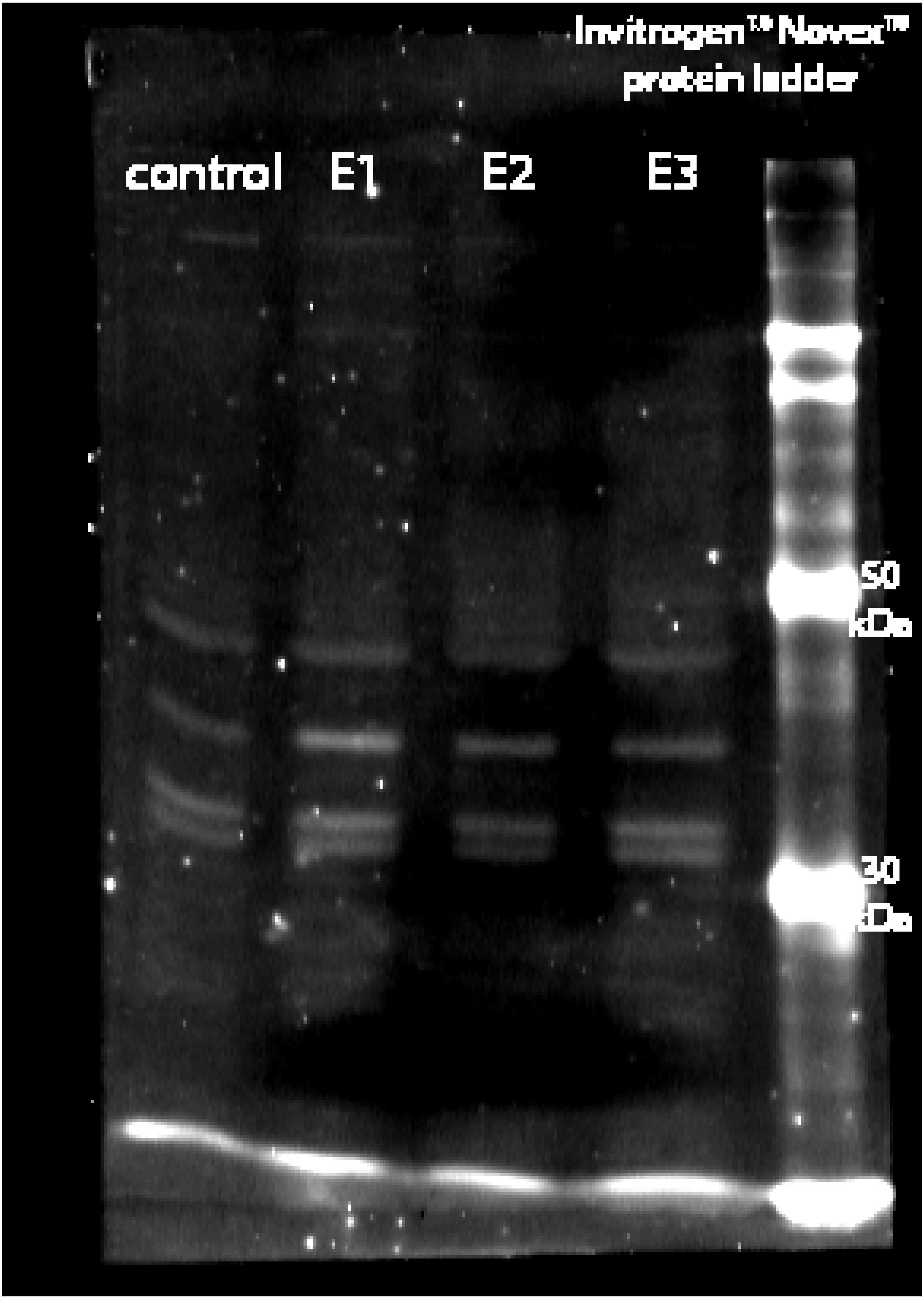
Invitrogen™ Novex™
protein ladder
control
E1
E2
E3
50
kDa
30
kDa

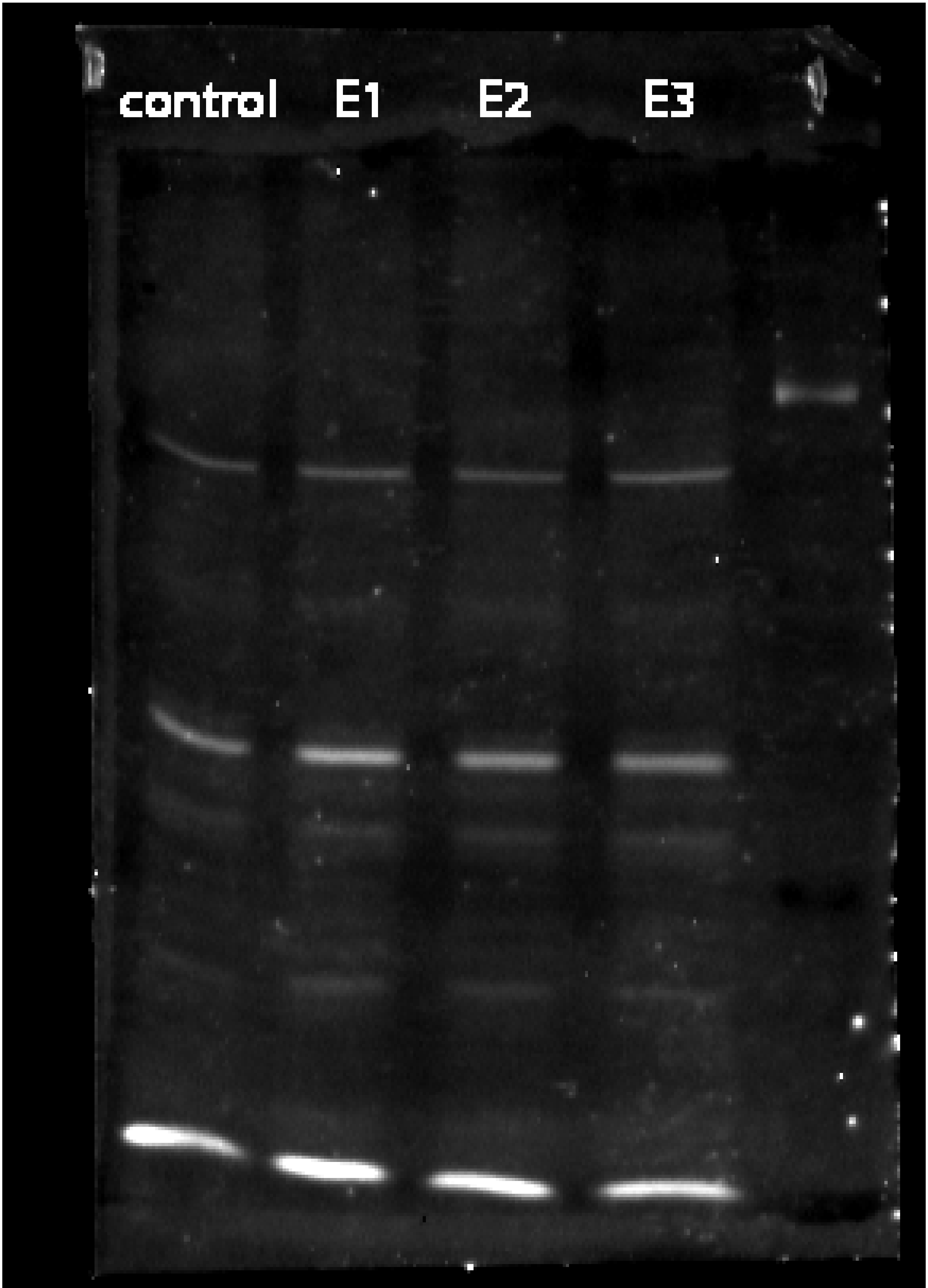
control
E1
E2
E3

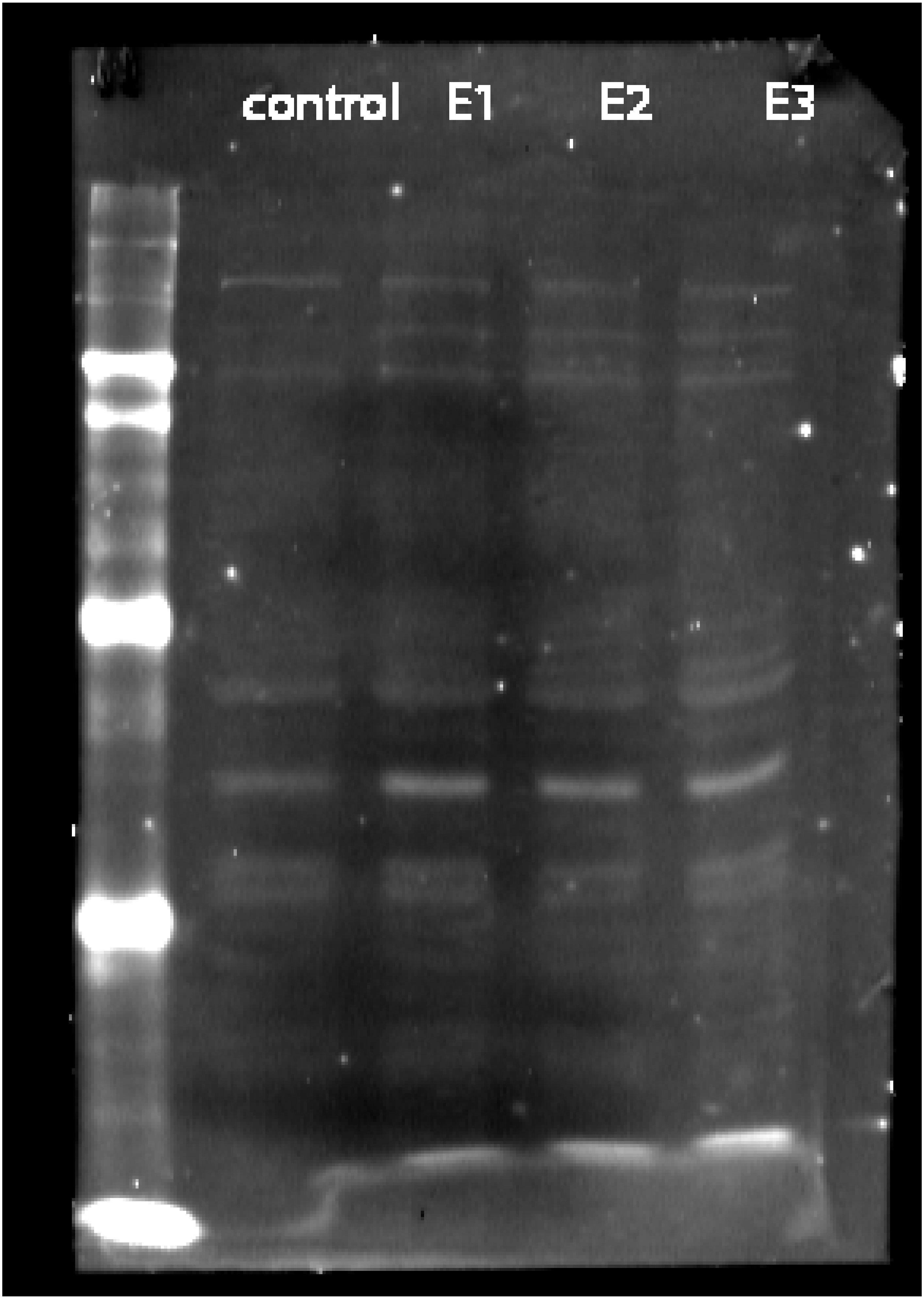
control
E1
E2
E3

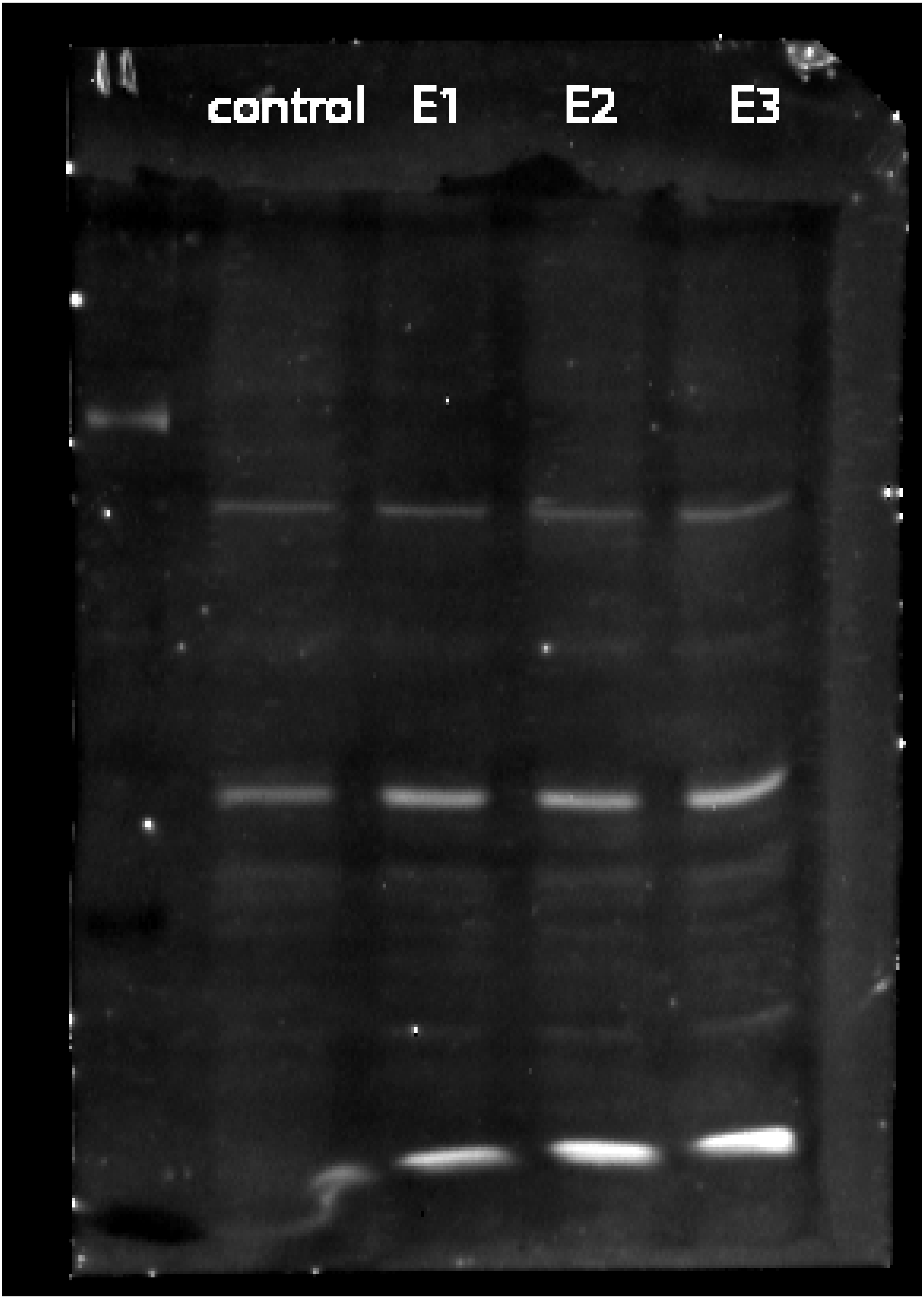
control
E1
E2
E3

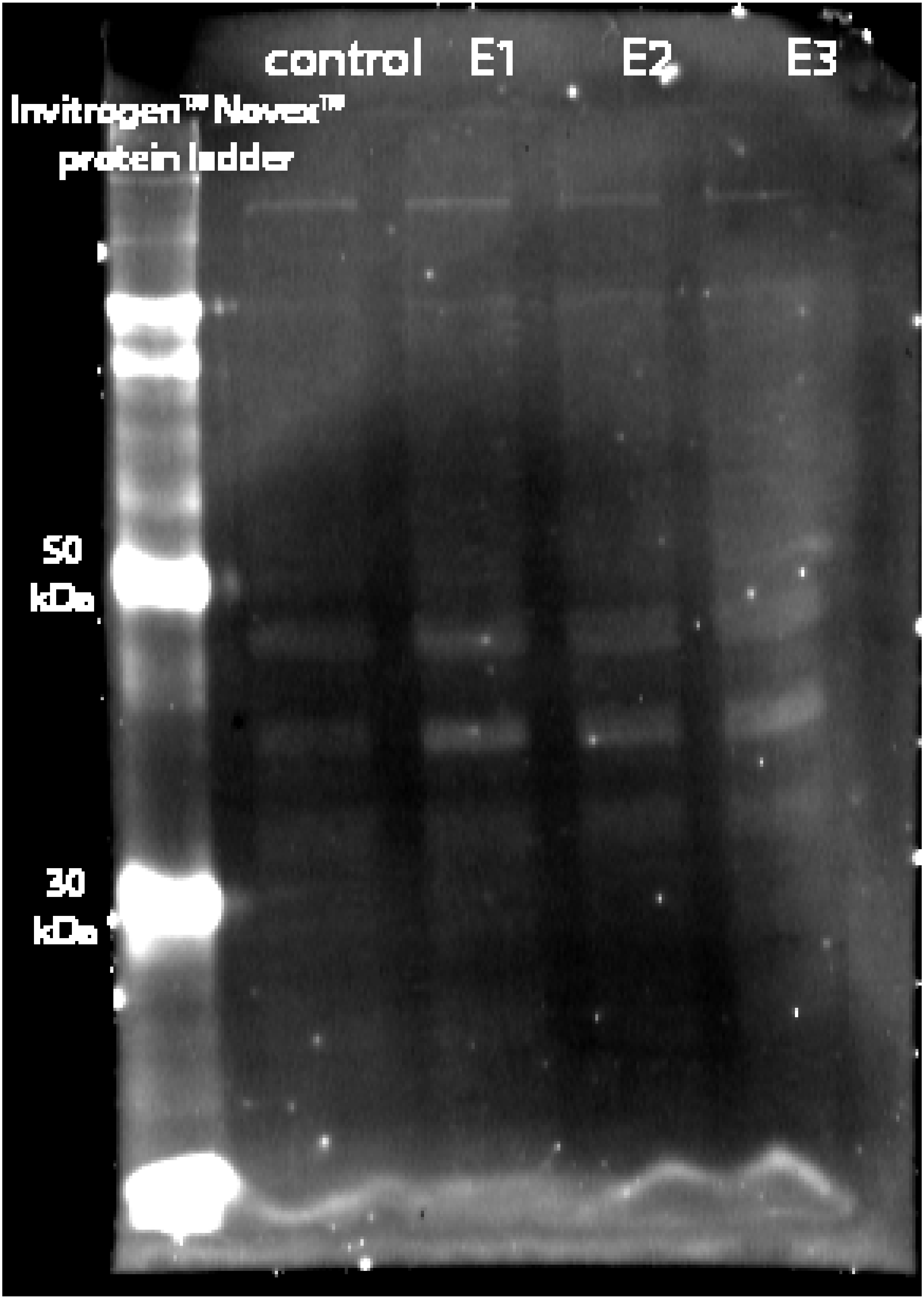
control
E1
E2
E3
Invitrogen™ Novex™
protein ladder
50
kDa
30
kDa

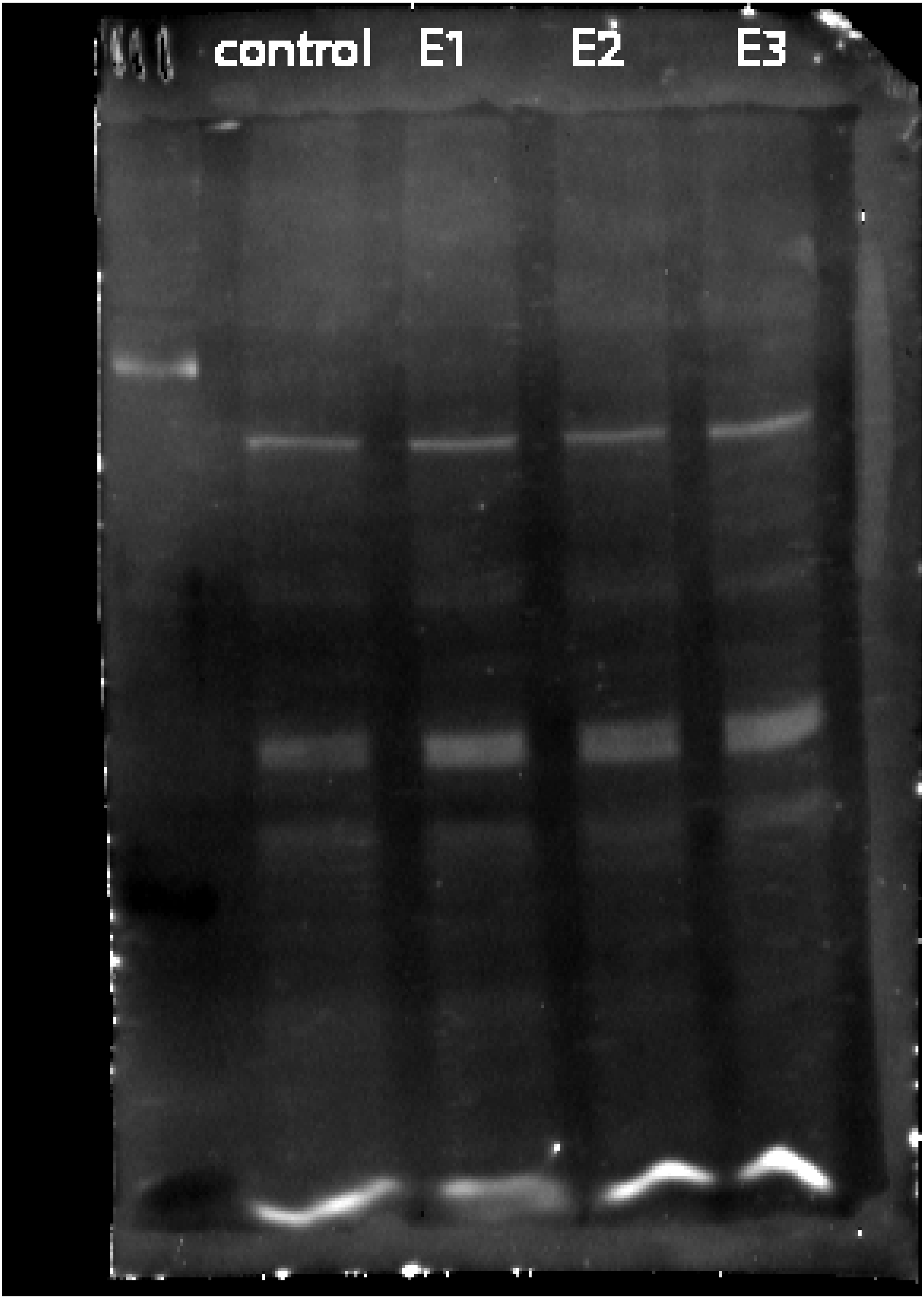
control
E1
E2
E3

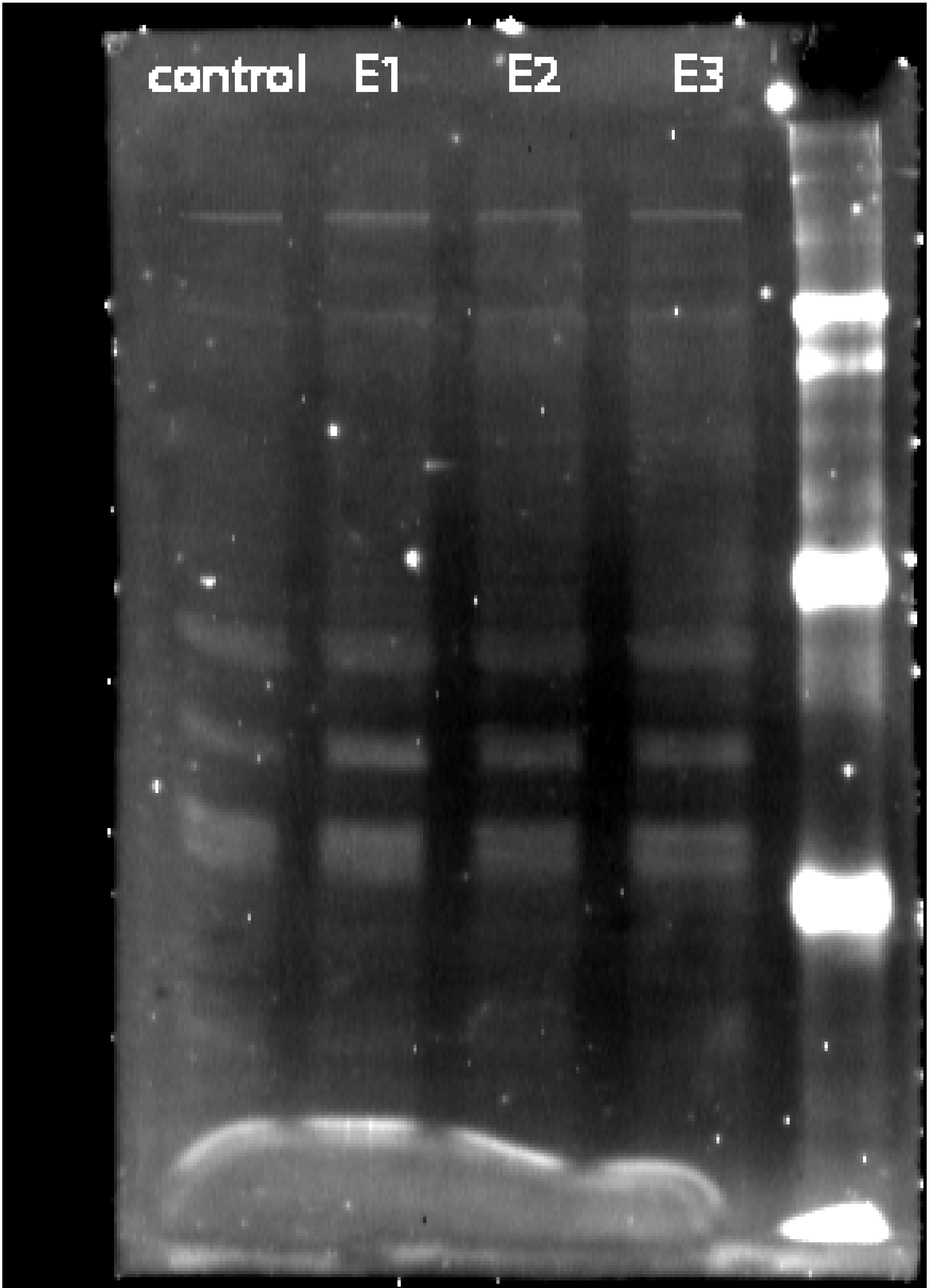
control
E1
E2
E3

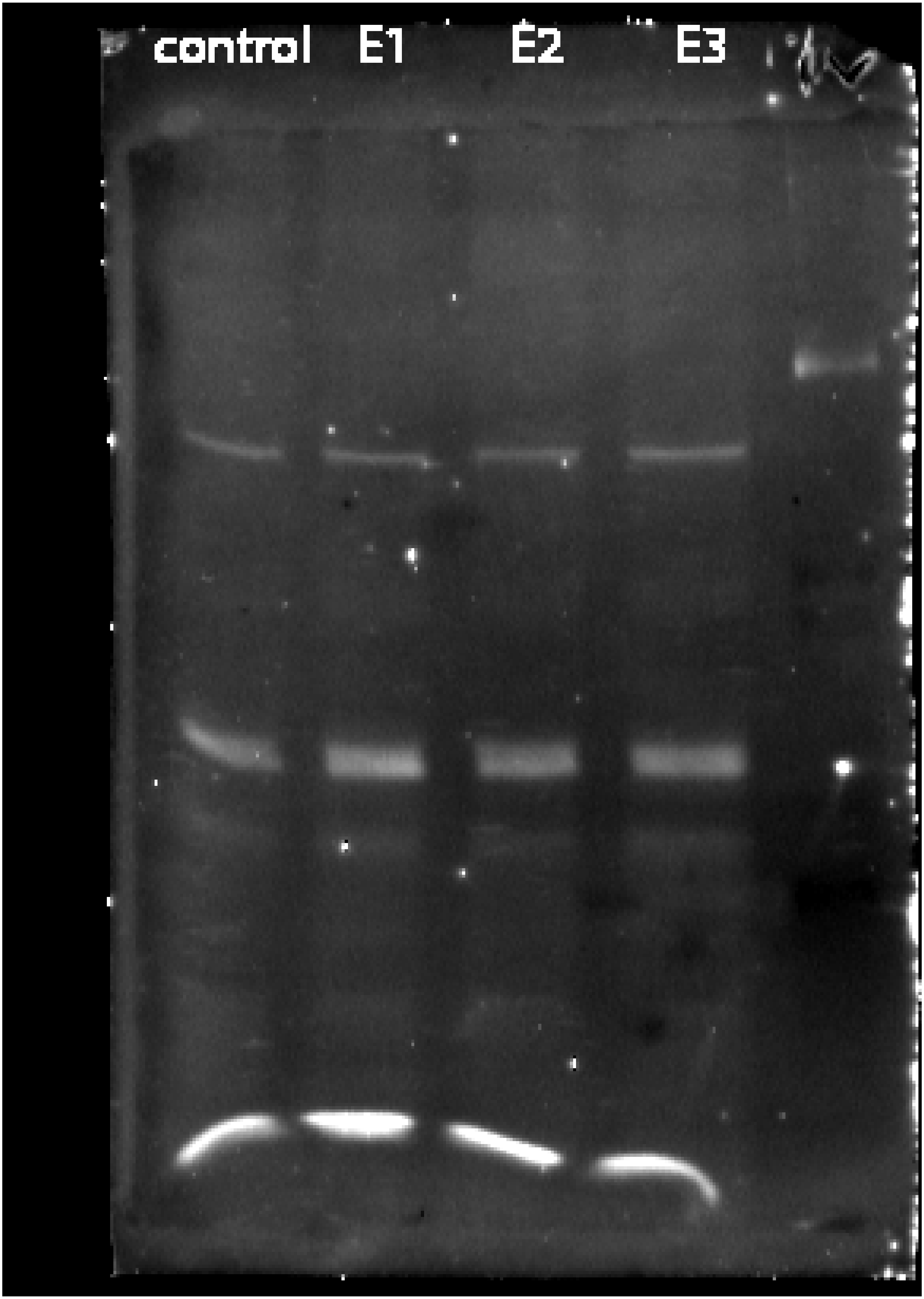
control
E1
E2
E3
