# Supplementary material for: Cannabis sativa extracts inhibit LDL oxidation and the formation of foam cells in vitro, acting as potential multi-step inhibitors of atherosclerosis development
Source: PLoS One. 2024 Dec 20;19(12):e0310777. doi: 10.1371/journal.pone.0310777 (PMC11661628; doi:10.1371/journal.pone.0310777)
Supplement: S1 Table — Effect of cannabis extracts and phytocannabinoids on LDL oxidation. (PDF) [file pone.0310777.s012.pdf]

Table S1. Effect of cannabis extracts and phytocannabinoids on LDL oxidation

Sample	CA μg^{-1}	CI_{50} ($\mu g\ mL^{-1}$)	Ref
**E1**	4.9 ± 0.1	2.2 (1.9-2.4)	
**E2**	4.0 ± 0.1	4.9 (4.2-6.9)	
**E3**	3.7 ± 0.1	8.0 (5.6-10.2)	(1)
**THCA**	1.6 ± 0.5	NE[a]	(1)
**THC**	21 ± 2	0.33 (0.32-0.37)	(1)
**CBDA**	9.4 ± 0.8	NE	(1)
**CBD**	4.8 ± 0.1	NE	(1)

[a]NE, not effective

Reference

(1). Musetti B, Gonzalez-Ramos H, Gonzalez M, Bahnson EM, Varela J, Thomson L. Cannabis sativa extracts protect LDL from Cu(2+)-mediated oxidation. J Cannabis Res. 2020;2.
