# Supplementary material for: Cannabis sativa extracts inhibit LDL oxidation and the formation of foam cells in vitro, acting as potential multi-step inhibitors of atherosclerosis development
Source: PLoS One. 2024 Dec 20;19(12):e0310777. doi: 10.1371/journal.pone.0310777 (PMC11661628; doi:10.1371/journal.pone.0310777)
Supplement: S2 Table — Binding affinities of cannabinoid receptors ligands assayed. (PDF) [file pone.0310777.s013.pdf]

Table S2. Binding affinities of cannabinoid receptors ligands assayed.

			Ki or IC_{50} (nM)								Ref
**ligand**	**receptor**		**CB1**	**CB2**	**TRPV1**	**TRPV2**	**TRPV3**	**TRPV4**	**TRPA1**	**GPR55**	
THC	CB1 - CB2	agonist	6-41	0.4-42	NA (too high)	650	9500	8500	230	8-8.9	[1–5]
THCA	CB1 - CB2	agonist	1292	1650	NA (too high)	18400	NA	3400	2700		[1,4]
CBD	CB1 - CB2	agonist	4350 >30000	2860 >30000	1000	1250	3700	800	110	354 Antagonist	[3–6]
CBDA	CB1 - CB2	agonist	626	813	19700	NA	NA	7600	5300		[2,4,7]
AEA	CB1 - CB2	agonist	31–252	27–581	270					18.4 10000	[2,3,5,8,9]
AM251	CB1	antagonist	3-8	124	NA 10000				Agonist	39- 10000	[5,10–16]
AM630	CB2	antagonist	5152	31	NA 10000				Agonist	~1500	[14,15,17,18]
Capsaicin	TRPV-1	agonist			94.8						[21]
AMG9810	TRPV-1	antagonist			1800 33						[19] [20]
ML184 = CID2440433	GPR55	agonist	>32000	>32000						260	[22–24]
CID16020046	GPR55	antagonist								63	[25]
GSK1016790A	TRPV4	agonist						5			[27]
HC067047	TRPV4	antagonist			>5000	>5000	>5000	17- 133	Weak activator >3000		[26,27]

- NA, No activity

References

[1] Husni, A.S.; McCurdy, C.R.; Radwan, M.M.; Ahmed, S.A.; Slade, D.; Ross, S.A.; ElSohly, M.A.; Cutler, S.J. Evaluation of Phytocannabinoids from High Potency Cannabis Sativa Using In Vitro Bioassays to Determine Structure-Activity Relationships for Cannabinoid Receptor 1 and Cannabinoid Receptor 2. *Med Chem Res*, **2014**, *23*, 4295–4300.

[2] Musetti, B.; Bahnson, E.M.; Thomson, L. Chapter 14 - Cannabinoids in Inflammation and Atherosclerosis. In: *Medicinal Usage of Cannabis and Cannabinoids*; Preedy, V.R.; Patel, V.B.; Martin, C.R., Eds.; Academic Press, **2023**; pp. 159–169.

[3] Showalter, V.M.; Compton, D.R.; Martin, B.R.; Abood, M.E. Evaluation of Binding in a Transfected Cell Line Expressing a Peripheral Cannabinoid Receptor (CB2): Identification of Cannabinoid Receptor Subtype Selective Ligands. *J Pharmacol Exp Ther*, **1996**, *278*, 989–999.

[4] De Petrocellis, L.; Ligresti, A.; Moriello, A.S.; Allarà, M.; Bisogno, T.; Petrosino, S.; Stott, C.G.; Di Marzo, V. Effects of Cannabinoids and Cannabinoid-Enriched Cannabis Extracts on TRP Channels and Endocannabinoid Metabolic Enzymes. *Br J Pharmacol*, **2011**, *163*, 1479–1494.

[5] Ryberg, E.; Larsson, N.; Sjögren, S.; Hjorth, S.; Hermansson, N.-O.; Leonova, J.; Elebring, T.; Nilsson, K.; Drmota, T.; Greasley, P.J. The Orphan Receptor GPR55 Is a Novel Cannabinoid Receptor. *Br. J. Pharmacol.*, **2007**, *152*, 1092–1101.

[6] Etemad, L.; Karimi, G.; Alavi, M.S.; Roohbakhsh, A. Pharmacological Effects of Cannabidiol by Transient Receptor Potential Channels. *Life Sciences*, **2022**, *300*, 120582.

[7] Navarro, G.; Varani, K.; Lillo, A.; Vincenzi, F.; Rivas-Santisteban, R.; Raïch, I.; Reyes-Resina, I.; Ferreiro-Vera, C.; Borea, P.A.; Sánchez de Medina, V.; Nadal, X.; Franco, R. Pharmacological Data of Cannabidiol- and Cannabigerol-Type Phytocannabinoids Acting on Cannabinoid CB1, CB2 and CB1/CB2 Heteromer Receptors. *Pharmacological Research*, **2020**, *159*, 104940.

[8] Mechoulam, R.; Peters, M.; Murillo-Rodriguez, E.; Hanus, L.O. Cannabidiol--Recent Advances. *Chem. Biodivers.*, **2007**, *4*, 1678–1692.

[9] Brown, A.J. Novel Cannabinoid Receptors. *Br J Pharmacol*, **2007**, *152*, 567–575.

[10] Kapur, A.; Zhao, P.; Sharir, H.; Bai, Y.; Caron, M.G.; Barak, L.S.; Abood, M.E. Atypical Responsiveness of the Orphan Receptor GPR55 to Cannabinoid Ligands. *J Biol Chem*, **2009**, *284*, 29817–29827.

[11] Henstridge, C.M.; Balenga, N.A.; Schröder, R.; Kargl, J.K.; Platzer, W.; Martini, L.; Arthur, S.; Penman, J.; Whistler, J.L.; Kostenis, E.; Waldhoer, M.; Irving, A.J. GPR55 Ligands Promote Receptor Coupling to Multiple Signalling Pathways. *Br J Pharmacol*, **2010**, *160*, 604–614.

[12] Sink, K.S.; McLaughlin, P.J.; Wood, J.A.T.; Brown, C.; Fan, P.; Vemuri, V.K.; Peng, Y.; Olszewska, T.; Thakur, G.A.; Makriyannis, A.; Parker, L.A.; Salamone, J.D. The Novel Cannabinoid CB1 Receptor Neutral Antagonist AM4113 Suppresses Food Intake and Food-Reinforced Behavior but Does Not Induce Signs of Nausea in Rats. *Neuropsychopharmacology*, **2008**, *33*, 946–955.

[13] Ross, R.A. Anandamide and Vanilloid TRPV1 Receptors. *Br. J. Pharmacol.*, **2003**, *140*, 790–801.

[14] Muller, C.; Morales, P.; Reggio, P.H. Cannabinoid Ligands Targeting TRP Channels. *Front Mol Neurosci*, **2019**, *11*, 487.

[15] Patil, M.; Patwardhan, A.; Salas, M.M.; Hargreaves, K.M.; Akopian, A.N. Cannabinoid Receptor Antagonists AM251 and AM630 Activate TRPA1 in Sensory Neurons. *Neuropharmacology*, **2011**, *61*, 778–788.

[16] Gatley, S.J.; Gifford, A.N.; Volkow, N.D.; Lan, R.; Makriyannis, A. 123I-Labeled AM251: A Radioiodinated Ligand Which Binds in Vivo to Mouse Brain Cannabinoid CB1 Receptors. *Eur J Pharmacol*, **1996**, *307*, 331–338.

[17] Ross, R.A.; Brockie, H.C.; Stevenson, L.A.; Murphy, V.L.; Templeton, F.; Makriyannis, A.; Pertwee, R.G. Agonist-Inverse Agonist Characterization at CB1 and CB2 Cannabinoid Receptors of L759633, L759656, and AM630. *Br J Pharmacol*, **1999**, *126*, 665–672.

[18] Hosohata, K.; Quock, R.M.; Hosohata, Y.; Burkey, T.H.; Makriyannis, A.; Consroe, P.; Roeske, W.R.; Yamamura, H.I. AM630 Is a Competitive Cannabinoid Receptor Antagonist in the Guinea Pig Brain. *Life Sci*, **1997**, *61*, PL115-118.

[19] Pearce, L.V.; Ann, J.; Blumberg, P.M.; Lee, J. Combination of a Rapidly Penetrating Agonist and a Slowly Penetrating Antagonist Affords Agonist Action of Limited Duration at the Cellular Level. *Biomol Ther (Seoul)*, **2019**, *27*, 435–441.

[20] Czikora, Á.; Lizanecz, E.; Bakó, P.; Rutkai, I.; Ruzsnavszky, F.; Magyar, J.; Pórszász, R.; Kark, T.; Facskó, A.; Papp, Z.; Édes, I.; Tóth, A. Structure-Activity Relationships of Vanilloid Receptor Agonists for Arteriolar TRPV1. *Br J Pharmacol*, **2012**, *165*, 1801–1812.

[21] Gavva, N.R.; Tamir, R.; Qu, Y.; Klionsky, L.; Zhang, T.J.; Immke, D.; Wang, J.; Zhu, D.; Vanderah, T.W.; Porreca, F.; Doherty, E.M.; Norman, M.H.; Wild, K.D.; Bannon, A.W.; Louis, J.-C.; Treanor, J.J.S. AMG 9810 [(E)-3-(4-t-Butylphenyl)-N-(2,3-Dihydrobenzo[b][1,4] Dioxin-6-Yl)Acrylamide], a Novel Vanilloid Receptor 1 (TRPV1) Antagonist with Antihyperalgesic Properties. *J Pharmacol Exp Ther*, **2005**, *313*, 474–484.

[22] Kotsikorou, E.; Madrigal, K.E.; Hurst, D.P.; Sharir, H.; Lynch, D.L.; Heynen-Genel, S.; Milan, L.B.; Chung, T.D.Y.; Seltzman, H.H.; Bai, Y.; Caron, M.G.; Barak, L.; Abood, M.E.; Reggio, P.H. IDENTIFICATION OF THE GPR55 AGONIST BINDING SITE USING A NOVEL SET OF HIGH POTENCY GPR55 SELECTIVE LIGANDS. *Biochemistry*, **2011**, *50*, 5633–5647.

[23] Heynen-Genel, S.; Dahl, R.; Shi, S.; Milan, L.; Hariharan, S.; Bravo, Y.; Sergienko, E.; Hedrick, M.; Dad, S.; Stonich, D.; Su, Y.; Vicchiarelli, M.; Mangravita-Novo, A.; Smith, L.H.; Chung, T.D.; Sharir, H.; Barak, L.S.; Abood, M.E. Screening for Selective Ligands for GPR55 - Agonists. In: *Probe Reports from the NIH Molecular Libraries Program*; National Center for Biotechnology Information (US): Bethesda (MD), **2010**.

[24] Drmota, T.; Greasley, P.; Groblewski, T. Screening Assays for Cannabinoid-Ligand-Type Modulators of Gpr55. WO2004074844A1, September 2, **2004**.

[25] Brown, A.J.; Castellano-Pellicena, I.; Haslam, C.P.; Nichols, P.L.; Dowell, S.J. Structure-Activity Relationship of the GPR55 Antagonist, CID16020046. *Pharmacology*, **2018**, *102*, 324–331.

[26] Lawhorn, B.G.; Brnardic, E.J.; Behm, D.J. Recent Advances in TRPV4 Agonists and Antagonists. *Bioorganic & Medicinal Chemistry Letters*, **2020**, *30*, 127022.

[27] Everaerts, W.; Zhen, X.; Ghosh, D.; Vriens, J.; Gevaert, T.; Gilbert, J.P.; Hayward, N.J.; McNamara, C.R.; Xue, F.; Moran, M.M.; Strassmaier, T.; Uykal, E.; Owsianik, G.; Vennekens, R.; De Ridder, D.; Nilius, B.; Fanger, C.M.; Voets, T. Inhibition of the Cation Channel TRPV4 Improves Bladder Function in Mice and Rats with Cyclophosphamide-Induced Cystitis. *Proc Natl Acad Sci U S A*, **2010**, *107*, 19084–19089.
